# Effectiveness of nirmatrelvir/ritonavir and molnupiravir in non-hospitalized adults with COVID-19: systematic review and meta-analysis of observational studies

**DOI:** 10.1093/jac/dkae163

**Published:** 2024-05-31

**Authors:** Yonatan M Mesfin, Joseph E Blais, Kelemu Tilahun Kibret, Teketo Kassaw Tegegne, Benjamin J Cowling, Peng Wu

**Affiliations:** School of Public Health, LKS Faculty of Medicine, World Health Organization Collaborating Centre for Infectious Disease Epidemiology and Control, The University of Hong Kong, Hong Kong Special Administration Region, Hong Kong, China; Immunity & Global Health, Murdoch Children’s Research Institute (MCRI), Parkville, VIC, Australia; School of Public Health, LKS Faculty of Medicine, World Health Organization Collaborating Centre for Infectious Disease Epidemiology and Control, The University of Hong Kong, Hong Kong Special Administration Region, Hong Kong, China; Laboratory of Data Discovery for Health (D^2^4H), Hong Kong Science and Technology Park, New Territories, Hong Kong Special Administration Region, Hong Kong, China; Centre for Safe Medication Practice and Research, Department of Pharmacology and Pharmacy, LKS Faculty of Medicine, The University of Hong Kong, Hong Kong Special Administration Region, Hong Kong, China; Global Centre for Preventive Health and Nutrition, Deakin University, Geelong, VIC, Australia; Institute for Physical Activity and Nutrition, Deakin University, Geelong, VIC, Australia; School of Public Health, LKS Faculty of Medicine, World Health Organization Collaborating Centre for Infectious Disease Epidemiology and Control, The University of Hong Kong, Hong Kong Special Administration Region, Hong Kong, China; Laboratory of Data Discovery for Health (D^2^4H), Hong Kong Science and Technology Park, New Territories, Hong Kong Special Administration Region, Hong Kong, China; School of Public Health, LKS Faculty of Medicine, World Health Organization Collaborating Centre for Infectious Disease Epidemiology and Control, The University of Hong Kong, Hong Kong Special Administration Region, Hong Kong, China; Laboratory of Data Discovery for Health (D^2^4H), Hong Kong Science and Technology Park, New Territories, Hong Kong Special Administration Region, Hong Kong, China

## Abstract

**Objective:**

To determine the effectiveness of nirmatrelvir/ritonavir and molnupiravir among vaccinated and unvaccinated non-hospitalized adults with COVID-19.

**Methods:**

Observational studies of nirmatrelvir/ritonavir or molnupiravir compared to no antiviral drug treatment for COVID-19 in non-hospitalized adults with data on vaccination status were included. We searched MEDLINE, EMBASE, Scopus, Web of Science, WHO COVID-19 Research Database and *medRxiv* for reports published between 1 January 2022 and 8 November 2023. The primary outcome was a composite of hospitalization or mortality up to 35 days after COVID-19 diagnosis. Risk of bias was assessed with ROBINS-I. Risk ratios (RR), hazard ratios (HR) and risk differences (RD) were separately estimated using random-effects models.

**Results:**

We included 30 cohort studies on adults treated with nirmatrelvir/ritonavir (*n* = 462 279) and molnupiravir (*n* = 48 008). Nirmatrelvir/ritonavir probably reduced the composite outcome (RR 0.62, 95%CI 0.55–0.70; *I*^2 ^= 0%; moderate certainty) with no evidence of effect modification by vaccination status (RR *P*_subgroup _= 0.47). In five studies, RD estimates against the composite outcome for nirmatrelvir/ritonavir were 1.21% (95%CI 0.57% to 1.84%) in vaccinated and 1.72% (95%CI 0.59% to 2.85%) in unvaccinated subgroups.

Molnupiravir may slightly reduce the composite outcome (RR 0.75, 95%CI 0.67–0.85; *I*^2 ^= 32%; low certainty). Evidence of effect modification by vaccination status was inconsistent among studies reporting different effect measures (RR *P*_subgroup _= 0.78; HR *P*_subgroup _= 0.08). In two studies, RD against the composite outcome for molnupiravir were −0.01% (95%CI −1.13% to 1.10%) in vaccinated and 1.73% (95%CI −2.08% to 5.53%) in unvaccinated subgroups.

**Conclusions:**

Among cohort studies of non-hospitalized adults with COVID-19, nirmatrelvir/ritonavir is effective against the composite outcome of severe COVID-19 independent of vaccination status. Further research and a reassessment of molnupiravir use among vaccinated adults are warranted.

**Registration:**

PROSPERO CRD42023429232.

## Introduction

Nirmatrelvir/ritonavir and molnupiravir are oral antiviral drugs that have facilitated early outpatient treatment of COVID-19. Emergency use authorization was granted for these drugs based on the results of pivotal placebo-controlled efficacy trials: Molnupiravir for oral treatment of COVID-19 in non-hospitalized patients (MOVe-OUT) and Evaluation of Protease Inhibition for COVID-19 in High-Risk patients (EPIC-HR).^1,2^ MOVe-OUT and EPIC-HR enrolled unvaccinated, younger adults, during the pre-Omicron era and demonstrated that both drugs reduced the risk of hospitalization or death at 4 weeks versus placebo.^1,2^

The applicability of this evidence to the current COVID-19 situation is highly uncertain.^3^ Population immunity, induced from vaccines, prior infection or both, has increased and coupled with emergence of the Omicron variants, has resulted in a lower risk of severe outcomes.^4,5^ Health systems and clinicians are now required to make treatment decisions considering the costs and potential harms, including antimicrobial resistance, of the available oral antiviral drugs in the context of SARS-CoV-2 endemicity.^6,7^

There is no robust evidence from randomized controlled trials (RCTs) to support the effectiveness of nirmatrelvir/ritonavir against hospitalization and death in adults vaccinated against COVID-19.^8,9^ The efficacy of nirmatrelvir/ritonavir in vaccinated adults in the EPIC-SR (standard risk) trial was also not confirmed because of the small sample size and low number of outcome events, resulting in imprecise estimates for the absolute risk difference (RD) versus placebo for the composite outcome of hospitalization and death (−1.29%, 95% CI −3.26 to 0.67).^10^ For molnupiravir, meta-analyses of RCTs have suggested that vaccination status is a potential effect modifier, although these results have low credibility.^8,11,12^ Furthermore, the Platform-Adaptive trial of Novel Antivirals for early treatment of COVID-19 in the Community (PANORAMIC) was the only study included in the vaccinated subgroup in these meta-analyses and it found no reduction in hospitalization and mortality with molnupiravir versus usual care.^13^

Evidence on the effects of oral antiviral drugs in RCTs has been extensively synthesized but a dearth of evidence on vaccinated subgroups persists.^8,9,11,12,14^ In response to this evidence gap, numerous observational studies have investigated the effectiveness of oral antiviral drugs for COVID-19 in the Omicron era, but the best available evidence from these observational studies has not been systematically appraised. Early systematic reviews of observational studies of nirmatrelvir/ritonavir have several limitations as they did not explicitly include studies that accounted for patient-level vaccination status and their search results are outdated (searches last performed in January 2023).^15,16^ Furthermore, there are no published systematic reviews of observational studies of molnupiravir. Whether there is a difference in the effectiveness of oral antiviral drugs between vaccinated and unvaccinated patients is essential information that can support decisions made by clinicians, guideline developers and regulators.^14^ This study therefore aimed to determine the effectiveness of nirmatrelvir/ritonavir and molnupiravir versus no oral antiviral treatment in reducing severe clinical outcomes among non-hospitalized adults stratified by their COVID-19 vaccination status, as assessed in observational studies.

## Methods

We registered the review protocol before conducting the initial literature search (PROSPERO CRD42023429232). This study-level systematic review of observational studies assessing the comparative effectiveness of nirmatrelvir/ritonavir or molnupiravir in non-hospitalized adults with COVID-19 was managed in Covidence (Veritas Health Innovation, Melbourne, Australia) and reported in accordance with the Preferred Reporting Items for Systematic reviews and Meta-Analyses (PRISMA) 2020 recommendations.^17^

### Literature search

We searched for published and preprint reports published between 1 January 2022 to 23 May 2023 using MEDLINE via OVID, EMBASE via OVID, Scopus, Web of Science, the WHO COVID-19 Research Database and *medRxiv*. An updated search was performed for studies published up to 8 November 2023. The search strategy was based on the defined patient, intervention, and outcome terms including ‘COVID-19’, ‘SARS-CoV-2’, ‘molnupiravir’, ‘Lagevrio’, ‘nirmatrelvir’, ‘Paxlovid’ and ‘hospitalization’ and ‘mortality’. As an example, the search strategy for MEDLINE is provided in Table S1 (available as Supplementary data at *JAC* Online).

### Eligibility criteria

We included observational studies with either a cohort or case-control design that enrolled non-hospitalized adults and assessed treatment initiation with nirmatrelvir/ritonavir or molnupiravir versus no antiviral treatment. Studies meeting the following criteria were included: most of the participants ≥18 years of age; nirmatrelvir/ritonavir and/or molnupiravir were the intervention with the treatment initiation period being specified after a positive test result for SARS-CoV2, the onset of COVID-19 symptoms or the date of diagnosis of COVID-19; defined the comparator as no treatment with the oral antiviral drug of interest; assessed mortality (all-cause or COVID-19 related) or hospitalization (all-cause or COVID-19 related) up to 35 days after the index date as the clinical outcome and accounted for the individual-level vaccine status of study participants whether through the study design or in the statistical analysis. We included both published and unpublished reports of original research in English. Studies were excluded that exclusively enrolled paediatric patients (<18 years of age), hospitalized patients or patients residing in long-term care or nursing homes, did not clearly report or did not have patient-level COVID-19 vaccination information, did not measure the target clinical outcomes, selected other drugs as comparators or had no comparator group, or selected a time point for outcome measurement beyond 35 days.

### Outcomes

The primary outcomes of interest were the composite outcome of severe COVID-19 (typically defined as hospitalization or death), hospitalization (all-cause or COVID-19 related) and mortality (all-cause or COVID-19 related) as defined in the original studies. To ensure a similar time point for outcome assessment, studies could assess time to event using survival analysis or assess the probability (risk or odds) of the outcome up to day 35 after the index date. To ensure outcomes were assessed at a similar time, and to avoid discarding any informative studies, we selected day 35 after the index date as the latest time point for measuring the outcomes of interest. We considered both relative and absolute risks of the composite severe outcome for patients treated with nirmatrelvir/ritonavir and molnupiravir in comparison with no treatment as reported in the original studies. If studies provided data on all-cause outcomes (e.g. hospitalization for any reason) and COVID-19 related outcomes (e.g. hospitalization specifically due to COVID-19), we included the all-cause outcomes in the quantitative synthesis since there is a lower risk of outcome misclassification bias. A detailed description of the individual study outcome definitions and their grouping in the meta-analyses is listed in Table S2.

### Study selection

Search results were imported to Covidence with duplicates removed automatically by the software. One author (Y.M.M.) screened titles and abstracts for potential relevance and assessed the full text for eligibility, and a second author (J.E.B.) independently verified the assessment and reasons for exclusion. Any discrepancies were resolved through discussion until consensus was achieved. The list of studies that were excluded after full text review and the reasons for exclusion can be found in Table S3.

### Data extraction

One author (Y.M.M. or J.E.B.) extracted data on the key study, patient, intervention, comparator, analytic, outcome and time variables (Tables S2 and S4). The sample size, number of events and type of outcome effect measure [risk ratio (RR), odds ratio (OR), hazard ratio (HR), or RD], the point estimate and the upper and lower bounds of the 95% CI for the overall study population and for each subgroup of interest were extracted for each outcome. A second reviewer (Y.M.M., J.E.B., T.K.T. or K.T.K.) independently checked the extracted data for accuracy. Given the variation in vaccination status definitions used in the original studies, when effect estimates among vaccinated subgroups were reported as more than two levels, we extracted estimates for the lowest level of COVID-19 vaccination exposure (e.g. 0 doses, 0–1 dose) and the boosted level (e.g.  ≥ 3 doses), and assigned them to unvaccinated and vaccinated subgroups, respectively. We applied a similar approach to age group classifications using the original age grouping to redefine the two age groups, 18–64 and ≥65 years, in the analysis (Table S2). There was insufficient information across studies to perform subgroup analyses according to time elapsed since the last COVID-19 vaccine dose.

### Risk of bias assessment

We used the ROBINS-I tool to assess the risk of bias in each observational study, specifically confounding bias, selection bias and information bias across six domains.^18^ Domain and study-level risk of bias were categorized as ‘low risk’, ‘moderate risk’, ‘serious risk’ or ‘critical risk’ of bias. For each study, one reviewer (J.E.B. or T.K.T.) assessed the risk of bias and a second reviewer verified the assessments (Y.M.M. or J.E.B.). Before the risk of bias assessment, we pre-specified the ideal target trial and made a list of the most important confounding domains that should be measured and controlled for in the study. Studies judged to be at critical risk of bias using ROBINS-I were excluded from the meta-analyses.^18,19^ Domain- and study-level risks of bias were visualized using the ROBVIS tool.^20^

### Synthesis methods

For each oral antiviral drug, we pooled the relative and absolute effect measures if ≥2 homogeneous studies were available for the outcome. For all analyses, we combined studies reporting a composite outcome of severe COVID-19, hospitalization or mortality, regardless of the outcome being all-causes or COVID-19-related. We pooled the adjusted effect measures for dichotomous outcomes (RR and OR) or time-to-event outcomes (HR) as reported in the eligible studies. For studies that reported HRs, effect estimates were pooled in separate meta-analysis models. For studies that reported ORs, prior to meta-analysis we converted adjusted ORs and their corresponding 95% CI to RRs (95% CI) using the formula described by Grant.^21^ In the formula, we used the median control group risks from the eligible studies that reported the absolute risks for each outcome (i.e. composite outcome = 3.7%, hospitalization = 3.1%, mortality = 1.1%).

In our primary analysis, RRs and RDs were estimated separately for nirmatrelvir/ritonavir and molnupiravir in the overall study populations where vaccination status was usually considered as a confounder variable. For relative effects only, we then performed *a priori* stratified analyses by vaccination status (vaccinated, unvaccinated) and age group (18–64,  ≥ 65 years) as defined in Table S2 using the within study subgroups when available. Where possible, we separately pooled RRs from HRs for each outcome in stratified analyses to examine consistency between studies reporting outcomes as dichotomous (OR and RR) and time-to-event (HR) effect measures. Additional pre-specified subgroup analyses by overall study-level risk of bias could not be conducted given the limited number of available studies included in each analysis. We performed a sensitivity analysis to separately combine studies according to their relative effect measure (OR, RR or HR), to assess consistency with estimates from our main analytic approach.

Given the anticipated between-study heterogeneity, we pooled relative or absolute estimates of each outcome using a random-effects model with inverse variance method. The restricted maximum likelihood estimator was used to calculate the heterogeneity variance estimator *τ*^2^.^22^ Between-study heterogeneity was quantified using the *I*^2^ statistic, which indicates the percentage of the variability in effect estimates that is due to between-study heterogeneity rather than sampling error.^23^ We tested for differences between subgroups using the *Q*-test, with a null hypothesis of no difference between effect sizes among studies across subgroups. RDs were calculated by subtracting the adjusted absolute risk of the outcome in the in oral antiviral drug group from the risk in the control group, with positive RDs indicating an associated reduction in the outcome with oral antiviral treatment. Number needed to treat (NNT) to prevent one outcome event was calculated as 1/RD. If a meta-analysis on an oral antiviral drug in relation to an outcome included 10 or more studies, funnel plots were generated, with the Egger’s and Begg and Mazumdar’s tests to assess for small study effects (funnel plot asymmetry) potentially caused by reporting bias.^24^ All analyses were performed using the *meta* package (v.6.5-0) in R version 4.3.2 (R Foundation for Statistical Computing, Vienna, Austria).

### Certainty assessment

We used the Grading of Recommendations Assessment, Development and Evaluation (GRADE) working group methodology to evaluate the certainty of evidence and created separate summary of findings tables for nirmatrelvir/ritonavir and molnupiravir.^25^

## Results

**Figure 1.**
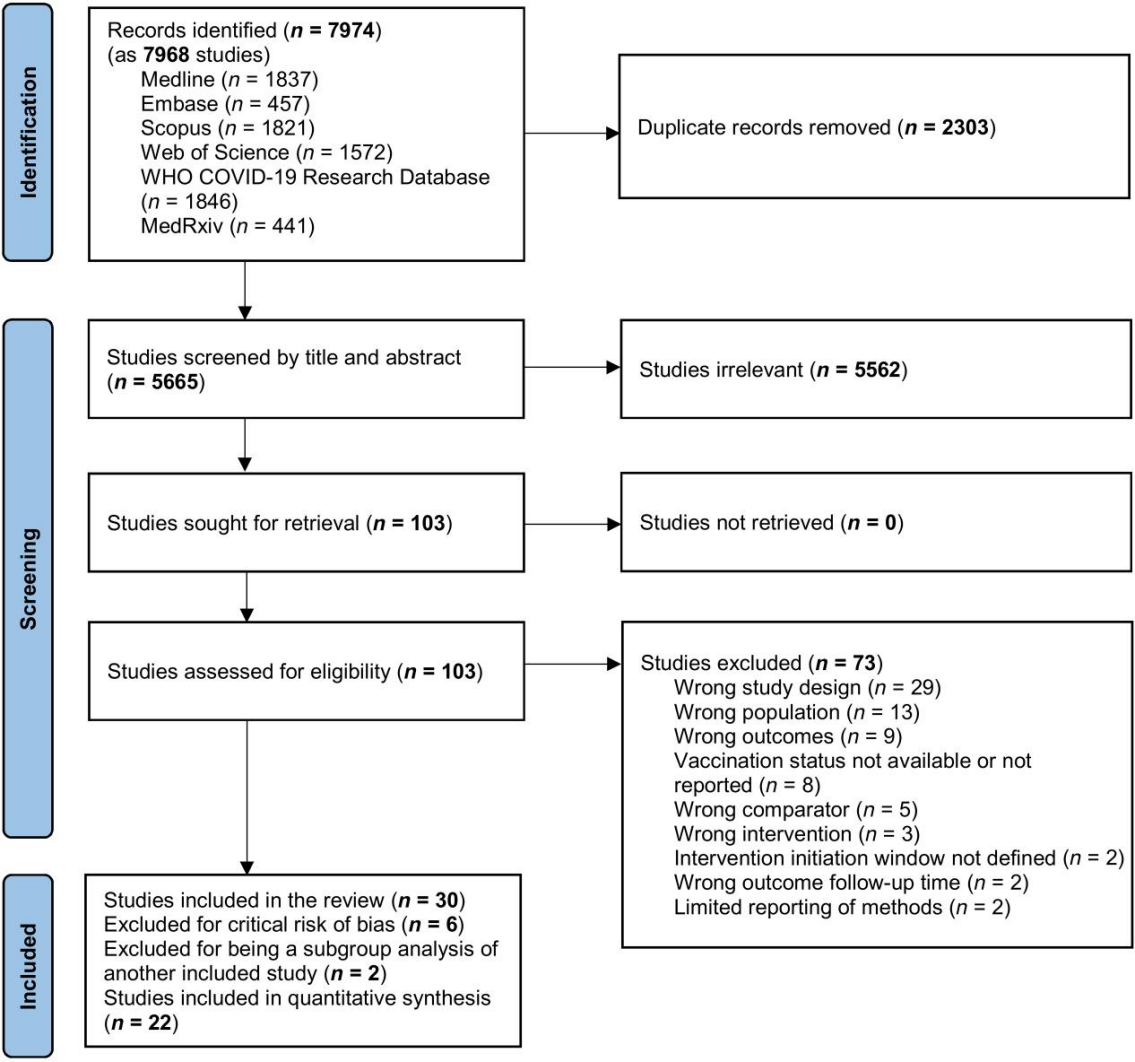
PRISMA flow diagram of studies in the systematic review of oral antiviral treatment of adult outpatients with COVID-19. This figure appears in colour in the online version of *JAC* and in black and white in the print version of *JAC*.

After screening titles and abstracts, 5562 studies were deemed irrelevant and were excluded (Figure 1). 103 studies were assessed in full text and 73 studies were excluded for not meeting the eligibility criteria (Table S3). We included a total of 30 cohort studies in the systematic review. After excluding six studies for critical risk of bias and two studies for being subgroups of other included studies, 22 cohort studies were included in the meta-analyses.^26–55^

The eligible cohort studies enrolled a total of 2 537 674 individuals (oral antivirals = 510 287; untreated controls = 2 027 387) of which 462 279 (91%) received nirmatrelvir/ritonavir and 48 008 (9%) received molnupiravir (Table S4). Studies reported on the effectiveness of nirmatrelvir/ritonavir (*n* = 17), molnupiravir (*n* = 5) or both oral antiviral drugs (*n* = 8) and were conducted across 12 geographical regions: USA (*n* = 10), Israel (*n* = 2), Greece (*n* = 2), Hong Kong (*n* = 3), England (*n* = 1), Wales (*n* = 1), Canada (*n* = 2), Australia (*n* = 1), Malaysia (*n* = 1), Singapore (*n* = 1), Italy (*n* = 1) and Serbia (*n* = 1). Four studies used data from a multinational database: TriNetX research Network.^35,36,40,53^

Studies using data from the Veterans Health Administration, Canada, Greece, Hong Kong, Australia, Singapore and Israel either exclusively enrolled or included mostly older adults.^27,29,30,32,38,42–47,50–52,54,55^ One study (Lewnard *et al.*) targeted patients ≥12 years but only included 11 065 individuals (nirmatrelvir/ritonavir = 11 and control = 11 054) 12–19 years of age from a total population of 133,426, and the outcomes were not specifically reported for adults ≥18 years.^39^ The WHO living guideline on COVID-19 therapeutics (v.14) has suggested that the estimated absolute risk of hospitalization be defined as 6% for patients at high risk and at 3% for patients at moderate risk.^8^ Where reported in the included studies, the average risk in the control groups exceeded the WHO ‘high risk’ threshold in only three studies, all in the subgroup of unvaccinated adults [Butt *et al.* (nirmatrelvir),^29^ Schwartz *et al*.,^47^ and Bajema *et al.* (molnupiravir)^27^; Table S2], suggesting that individuals in the included studies were predominantly at moderate or high risk of hospitalization.

Nearly all studies identified individuals during calendar periods of Omicron predominance. Fourteen studies specifically included patients with COVID-19 who had at least one documented risk factor related to progression to severe COVID-19 disease,^27,28,30–32,34,37,43,44,46,48,49,54,55^ while the remaining studies enrolled patients treated with oral antiviral drugs regardless of potential risk factors. One study (Kwok *et al.*^38^) and two studies (Ganatra *et al.*^36^ and Faust *et al.*^35^) exclusively enrolled unvaccinated and vaccinated patients, respectively.^35,36,38^ The remaining studies included patients regardless of vaccination status and reported overall effectiveness data for the entire study population or separately for vaccinated and unvaccinated subgroups.

### Risk of bias assessment

Overall study-level risk of bias tended to be serious or critical, primarily due to risk from confounding, selection bias and reporting bias (Figure S1a). Across the 30 included cohort studies, the risk of bias was found to be low in both the classification of interventions and the measurement of outcomes, whereas nearly all studies reported no information to assess bias due to deviations from the intended interventions. Six studies were judged to be at a critical risk of bias for not controlling for important confounders, controlling for post-intervention variables, excluding a large amount of follow-up time, or having the confounding variables only recorded for the intervention group (Table S5).^37,38,40,45,46,53^ Sixteen studies were judged to be at a serious risk of bias primarily for not measuring or controlling for important confounding domains (e.g. concomitant prescription of interacting drugs, limitations in determining vaccination status).^26,28–31,34–36,39,41,42,44,48,50–52^ No studies were judged to be at a low risk, and among the remaining eight studies at a moderate risk of bias^27,32,33,43,47,49,54,55^ six were at a low risk of bias on four or more domains (Figure S1b).^27,32,43,49,54,55^ Given the limited number of studies for each oral antiviral drug and outcome comparison, we did not generate funnel plots for any of the meta-analyses.

### Effectiveness of nirmatrelvir/ritonavir and molnupiravir

**Figure 2.**
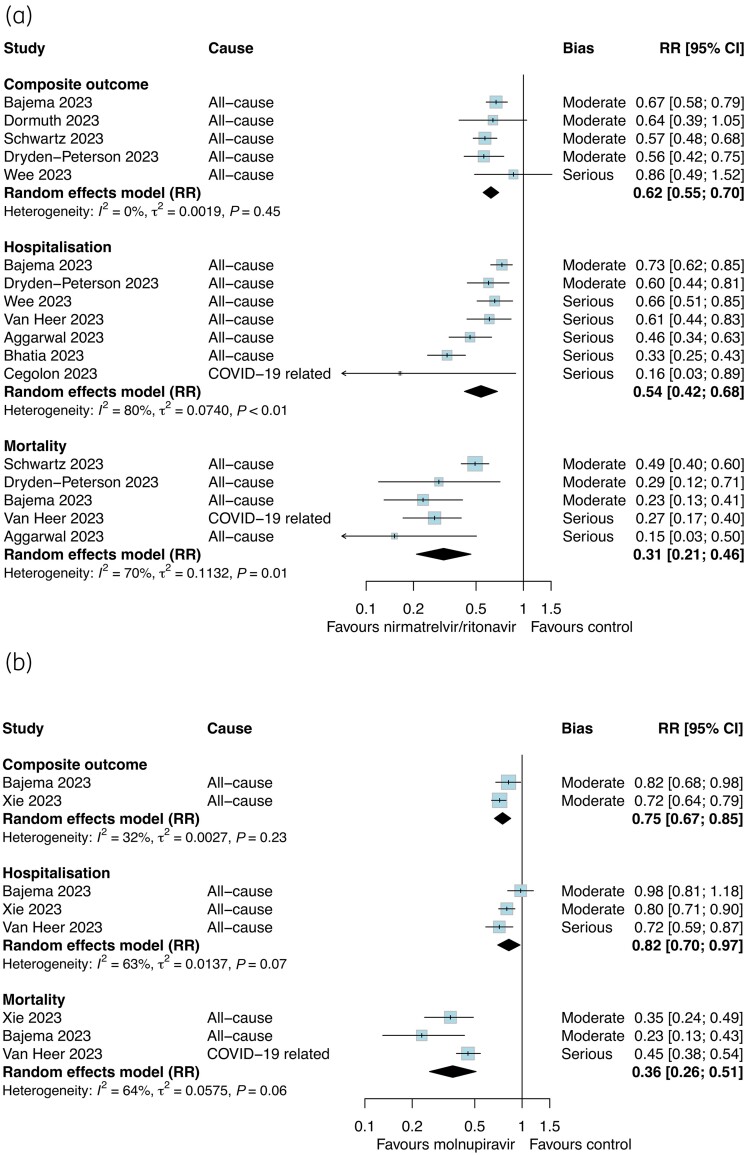
Relative effectiveness of nirmatrelvir/ritonavir (a) and molnupiravir (b) in the overall cohort populations (considered as a confounder) against the composite outcome, hospitalization and mortality. The size of the data markers (blue squares) reflects the weight of each study and its contribution to the pooled estimate. CI, confidence interval. This figure appears in colour in the online version of *JAC* and in black and white in the print version of *JAC*.

**Figure 3.**
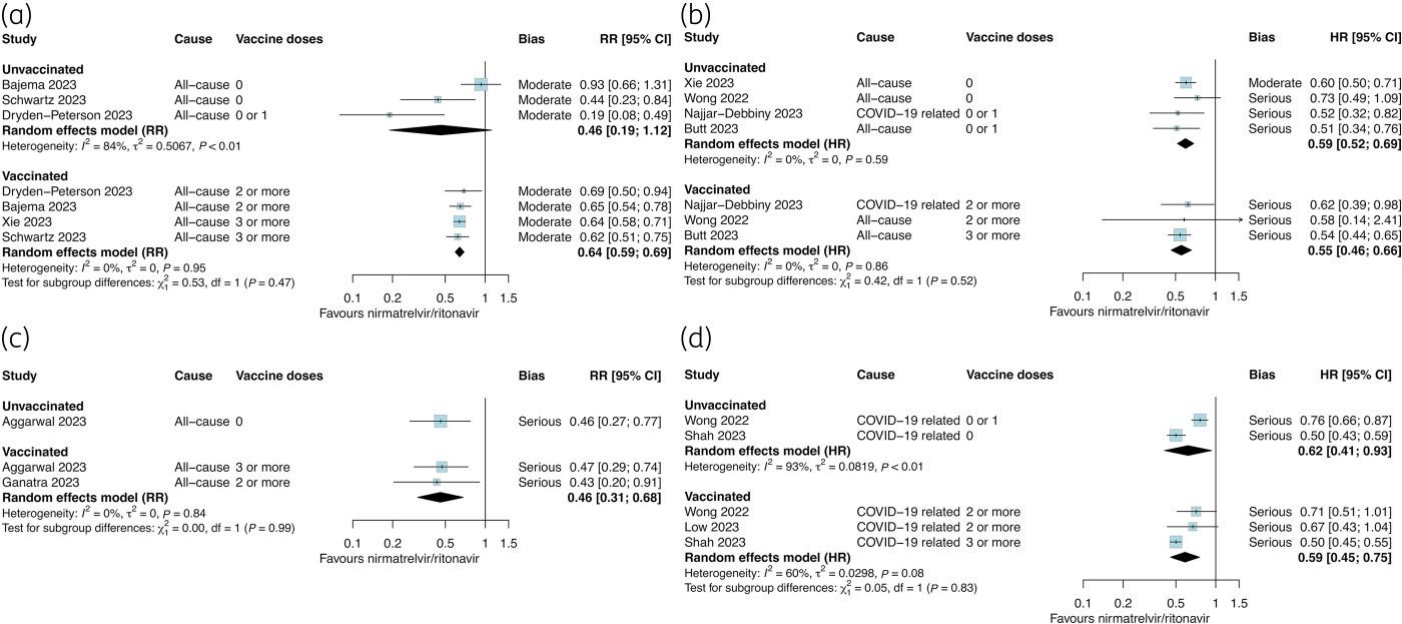
Relative effectiveness of nirmatrelvir/ritonavir among vaccinated and unvaccinated subgroups against (a) composite outcome (RR), (b) composite outcome (HR), (c) hospitalization (RR) and (d) hospitalization (HR). The size of the data markers (blue squares) reflects the weight of each study and its contribution to the pooled estimate. CI, confidence interval. This figure appears in colour in the online version of *JAC* and in black and white in the print version of *JAC*.

Table 1 presents the summary of findings for nirmatrelvir/ritonavir and the estimated absolute effects for each outcome. Overall, we had moderate certainty that nirmatrelvir/ritonavir probably reduced the composite outcome (RR 0.62, 95% CI 0.55–0.70; *I*^2 ^= 0%) and mortality (RR 0.31, 95% CI 0.21–0.44; *I*^2 ^= 63%) and low certainty that it slightly reduced hospitalization (RR 0.54, 95% CI 0.42–0.68; *I*^2 ^= 80%; Figure 2a). When stratified by vaccination status, there were consistent associations and limited evidence of effect modification for the composite outcome (Figure 3a and 3b) and hospitalization (Figure 3c and 3d). There were insufficient studies with the same effect measure that reported the outcome of mortality by vaccination status. Two studies (Wong *et al*.^52^ and Schwartz *et al*.^47^) could not be pooled in meta-analysis but reported that nirmatrelvir/ritonavir was associated within reductions in mortality in vaccinated and unvaccinated subgroups.^47,52^

**Table 1.** Summary of findings table for nirmatrelvir/ritonavir

Nirmatrelvir/ritonavir compared to no antiviral drug for adults with COVID-19						
Patient or population: adults with COVID-19 Setting: non-hospitalized (outpatients) Intervention: nirmatrelvir/ritonavir Comparison: no antiviral drug						
Outcome Number of participants (studies)	Relative effect (95% CI)	Anticipated absolute effects (95% CI)			Certainty	What happens
		No antiviral drug	Nirmatrelvir/ritonavir	Difference		
Composite outcome follow-up: range 28 to 30 days Number of of participants: 781 038 (10 non-randomized studies)	RR 0.62 (0.55 to 0.70)	Overall			⨁⨁⨁○ Moderate^a^	Nirmatrelvir/ritonavir probably results in a reduction in composite outcome overall and among vaccinated and unvaccinated subgroups.
		3.7%	2.3% (2 to 2.6)	1.4% fewer (1.7 fewer to 1.1 fewer)		
		Vaccinated				
		3.3%	2.0% (1.8 to 2.3)	1.3% fewer (1.5 fewer to 1 fewer)		
		Unvaccinated				
		5.2%	3.2% (2.9 to 3.6)	2.0% fewer (2.3 fewer to 1.6 fewer)		
Hospitalization follow-up: range 14 days to 35 days Number of of participants: 1 474 514 (10 non-randomized studies)	RR 0.54 (0.42 to 0.68)	Overall			⨁⨁○○ Low^b^	Nirmatrelvir/ritonavir may result in a slight reduction in hospitalization overall and among vaccinated and unvaccinated subgroups.
		3.1%	1.7% (1.3 to 2.1)	1.4% fewer (1.8 fewer to 1 fewer)		
		Vaccinated				
		3.1%	1.7% (1.3 to 2.1)	1.4% fewer (1.8 fewer to 1 fewer)		
		Unvaccinated				
		5.0%	2.7% (2.1 to 3.4)	2.3% fewer (2.9 fewer to 1.6 fewer)		
Mortality follow-up: range 28 days to 30 days Number of participants: 336 518 (6 non-randomized studies)	RR 0.31 (0.21 to 0.46)	Overall			⨁⨁⨁○ Moderate^c^	Nirmatrelvir/ritonavir probably results in a reduction in mortality overall, but evidence of benefit among vaccinated and unvaccinated subgroups is more limited (2 studies, not pooled).
		1.1%	0.3% (0.2 to 0.5)	0.8% fewer (0.9 fewer to 0.6 fewer)		
		Vaccinated				
		0.8%	0.2% (0.2 to 0.4)	0.6% fewer (0.6 fewer to 0.4 fewer)		
		Unvaccinated				
		1.4%	0.4% (0.3 to 0.6)	1.0% fewer (1.1 fewer to 0.8 fewer)		
*The risk in the intervention group (and its 95% confidence interval) is based on the assumed risk in the comparison group and the relative effect of the intervention (and its 95% CI). CI: confidence interval; RR: risk ratio						
GRADE Working Group grades of evidence High certainty: we are very confident that the true effect lies close to that of the estimate of the effect. Moderate certainty: we are moderately confident in the effect estimate: the true effect is likely to be close to the estimate of the effect, but there is a possibility that it is substantially different. Low certainty: our confidence in the effect estimate is limited: the true effect may be substantially different from the estimate of the effect. Very low certainty: we have very little confidence in the effect estimate: the true effect is likely to be substantially different from the estimate of effect.						

^a^Six studies were judged to be at serious risk of bias.

^b^Eight studies were judged to be at serious risk of bias.

^c^Three studies were judged to be at serious risk of bias.

**Figure 4.**
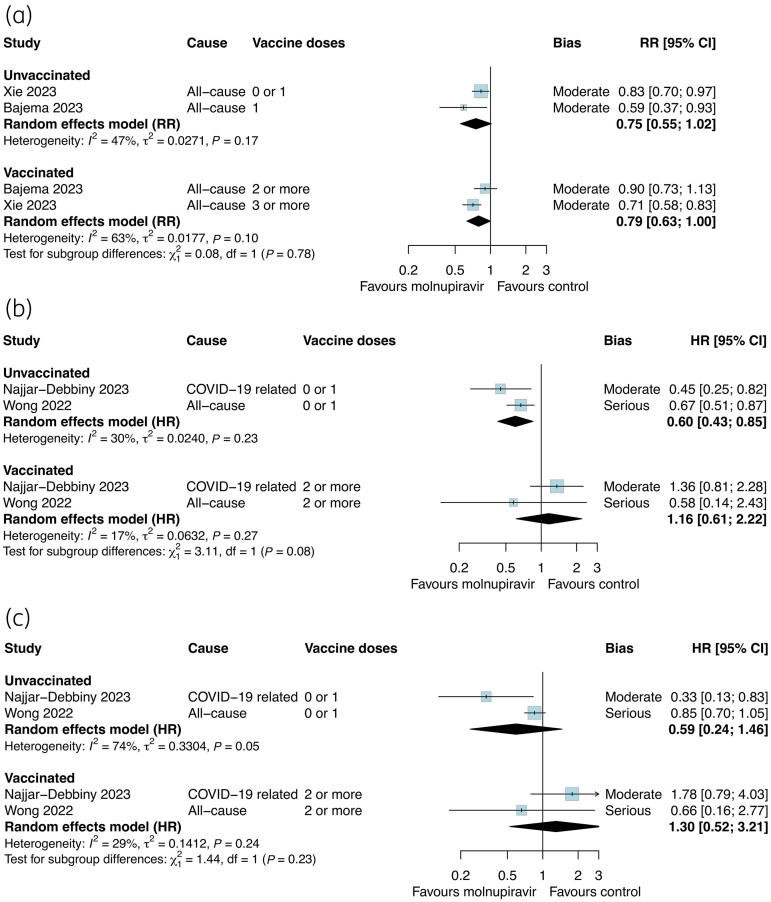
Relative effectiveness of molnupiravir among vaccinated and unvaccinated subgroups against (a) composite outcome (RR), (b) composite outcome (HR) and (c) mortality (HR). The size of the data markers (blue squares) reflects the weight of each study and its contribution to the pooled estimate. CI, confidence interval. This figure appears in colour in the online version of *JAC* and in black and white in the print version of *JAC*.

Table 2 presents the summary of findings for molnupiravir and the estimated absolute effects for each outcome. Molnupiravir may have slightly reduced the composite outcome (RR 0.75, 95% CI 0.67–0.85; *I*^2 ^= 32%; low certainty) but probably has little effect on hospitalization (RR 0.82, 95% CI 0.70–0.97; *I*^2 ^= 63%; moderate certainty; Figure 2b). We had very low certainty about the effect of molnupiravir on mortality (RR 0.36, 95% CI 0.26–0.51; *I*^2 ^= 64%). There was no strong evidence of effect modification by vaccination status for the composite outcome, but this varied between studies reporting RR (*P*_subgroup _= 0.78, Figure 4a) and those reporting HR (*P*_subgroup _= 0.08, Figure 4b). In two studies, molnupiravir was not associated with a reduction in mortality among vaccinated or unvaccinated adults (*P*_subgroup _= 0.23, Figure 4c) although the estimates were imprecise.

**Table 2.** Summary of findings table for molnupiravir

Molnupiravir compared to no antiviral drug for adults with COVID-19						
Patient or population: adults with COVID-19 Setting: non-hospitalized (outpatients) Intervention: molnupiravir Comparison: no antiviral drug						
Outcome Number of participants (studies)	Relative effect (95% CI)	Anticipated absolute effects (95% CI)			Certainty	What happens
		No antiviral drug	Molnupiravir	Difference		
Composite outcome follow-up: range 28 days to 30 days Number of participants: 226 523 (6 non-randomized studies)	RR 0.75 (0.67 to 0.85)	Overall			⨁⨁○○ Low^a,b^	Molnupiravir may result in a slight reduction in composite outcome overall and in unvaccinated adults, and may not reduce the composite outcome in vaccinated adults.
		3.7%	2.8% (2.5 to 3.1)	0.9% fewer (1.2 fewer to 0.6 fewer)		
		Vaccinated				
		3.3%	2.5% (2.2 to 2.8)	0.8% fewer (1.1 fewer to 0.5 fewer)		
		Unvaccinated				
		5.2%	3.9% (3.5 to 4.4)	1.3% fewer (1.7 fewer to 0.8 fewer)		
Hospitalization follow-up: range 28 days to 35 days Number of participants: 173 533 (4 non-randomized studies)	RR 0.82 (0.70 to 0.97)	Overall			⨁⨁⨁○ Moderate^a^	Molnupiravir probably results in little to no difference in hospitalization.
		3.1%	2.5% (2.2 to 3)	0.6% fewer (0.9 fewer to 0.1 fewer)		
		Vaccinated				
		3.1%	2.5% (2.2 to 3)	0.6% fewer (0.9 fewer to 0.1 fewer)		
		Unvaccinated				
		5.0%	4.1% (3.5 to 4.9)	0.9% fewer (1.5 fewer to 0.2 fewer)		
Mortality follow-up: range 28 days to 35 days Number of participants: 186 228 (5 non-randomized studies)	RR 0.36 (0.26 to 0.51)	Overall			⨁○○○ Very low^a,c^	The evidence is very uncertain about the effect of molnupiravir on mortality.
		1.1%	0.4% (0.3 to 0.6)	0.7% fewer (0.8 fewer to 0.5 fewer)		
		Vaccinated				
		0.8%	0.3% (0.2 to 0.4)	0.5% fewer (0.6 fewer to 0.4 fewer)		
		Unvaccinated				
		1.4%	0.5% (0.4 to 0.7)	0.9% fewer (1 fewer to 0.7 fewer)		
*The risk in the intervention group (and its 95% confidence interval) is based on the assumed risk in the comparison group and the relative effect of the intervention (and its 95% CI). CI: confidence interval; RR: risk ratio						
GRADE Working Group grades of evidence High certainty: we are very confident that the true effect lies close to that of the estimate of the effect. Moderate certainty: we are moderately confident in the effect estimate: the true effect is likely to be close to the estimate of the effect, but there is a possibility that it is substantially different. Low certainty: our confidence in the effect estimate is limited: the true effect may be substantially different from the estimate of the effect. Very low certainty: we have very little confidence in the effect estimate: the true effect is likely to be substantially different from the estimate of effect.						

^a^Two studies were judged to be at serious risk of bias.

^b^Confidence intervals for the estimates of absolute risk (overall and by vaccination status) include both potential benefit and harm.

^c^Confidence intervals for the estimates of absolute and relative risk (by vaccination status) include significant benefit and harm.

**Figure 5.**
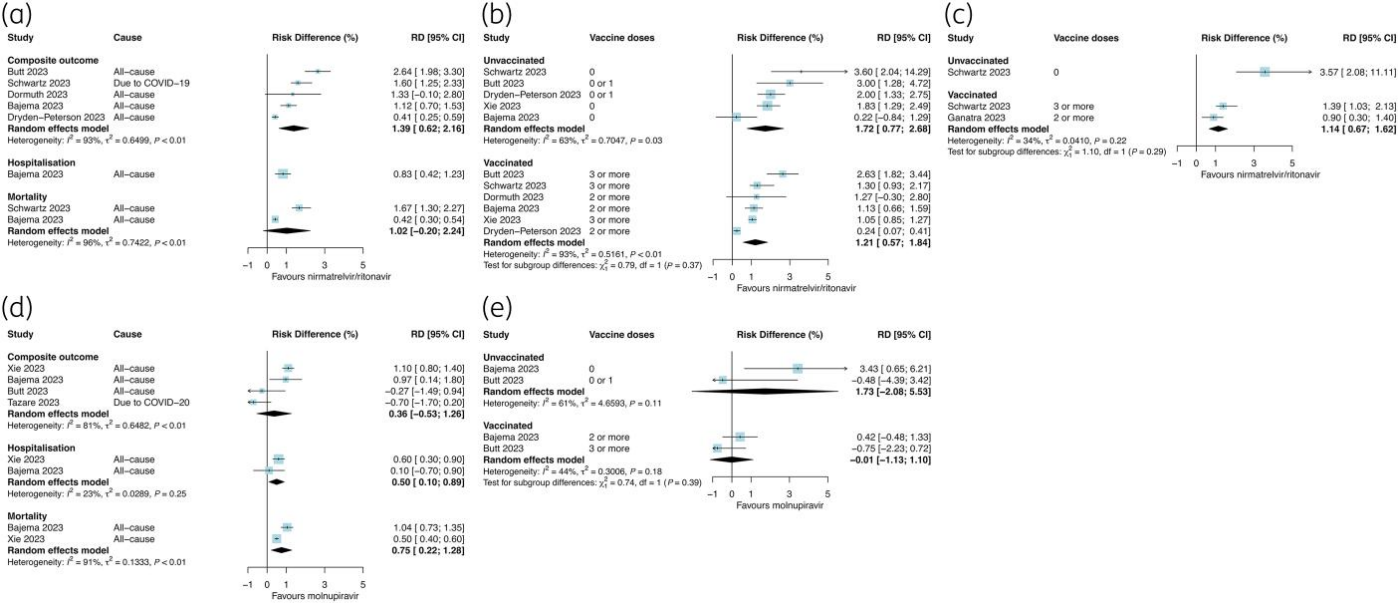
Absolute RD for nirmatrelvir/ritonavir for all outcomes among adults overall (a); composite outcome (b) and mortality (c) stratified by vaccination status. Absolute RD for molnupiravir for all outcomes among adults overall (d) and for the composite outcome stratified by vaccination status (e). The size of the data markers (blue squares) reflects the weight of each study and its contribution to the pooled estimate. CI, confidence interval. This figure appears in colour in the online version of *JAC* and in black and white in the print version of *JAC*.

Nirmatrelvir/ritonavir was associated with an overall lower absolute risk of the composite outcome (RD 1.39%, 95% CI 0.62%–2.16%; *I*^2 ^= 93%, five studies; Figure 5a) with an estimated NNT of 72 (95% CI 46–161). Only one study assessed nirmatrelvir/ritonavir against hospitalization while there was no associated reduction in all-cause mortality in two studies (RD 1.02%, 95% CI −0.20%–2.24%; *I*^2 ^= 96%; Figure 5a). RDs against the composite outcome by vaccination status were similar, but numerically lower, for unvaccinated (RD 1.72%, 95% CI 0.77%–2.68%; *I*^2 ^= 63%; 5 studies) and vaccinated subgroups (RD 1.21%, 95% CI 0.57%–1.84%; *I*^2 ^= 93%, six studies; *P*_subgroup _= 0.37; Figure 5b), but a high degree of heterogeneity was noted across studies, particularly the vaccinated subgroups that probably varied by baseline risk. Corresponding NNTs for the composite outcome at 28–35 days were 58 (95% CI 37–130) for unvaccinated and 83 (95% CI 55–176) for vaccinated adults. In two studies, the RD for nirmatrelvir/ritonavir treatment against mortality among vaccinated adults was 1.14% (95% CI 0.67%–1.62%; *I*^2 ^= 34%; NNT 88, 95% CI 62–150) and in one study it was 3.57% (95% CI 2.08–11.11; NNT 28, 95%CI 9–48; Figure 5c) among unvaccinated adults.

Molnupiravir was not associated with the composite outcome (RD 0.36%, 95% CI −0.53% to 1.26%; *I*^2 ^= 81%; four studies; Figure 5d) in the overall study populations. In two studies, molnupiravir was associated with absolute reductions in hospitalization (RD 0.50%, 95%CI 0.10%–0.89%; *I*^2 ^= 23%; NNT 200, 95% CI 112–1000) and mortality (RD 0.75%, 95% CI 0.22%–1.28%; *I*^2 ^= 91%; NNT 133, 95% CI 78–455; Figure 5d). There was no evidence of effect modification by vaccination status for molnupiravir against the composite outcome (Figure 5e).

### Subgroup and sensitivity analysis

Nirmatrelvir/ritonavir was associated with similar reductions in the composite outcome and hospitalization by age (Figure S2). Only one study (Schwartz *et al*.^47^) reported the effectiveness of nirmatrelvir/ritonavir against mortality among younger adults and there was some evidence of effect modification for mortality. For molnupiravir, there was strong evidence of effect modification by age for studies reporting HR (*P*_subgroup _< 0.01) but not RR (*P*_subgroup _= 0.46) for the composite outcome. Similarly for mortality, there was strong evidence of effect modification by age (*P*_subgroup _< 0.01) in two studies reporting HR (Figure S3). Pooled estimates suggested a potential increase in mortality with molnupiravir treatment among adults aged 18–64 years (HR 3.01, 95% CI 1.24–7.33; *I*^2 ^= 0%). In the sensitivity analysis by study-level effect measure, the relative effects of nirmatrelvir/ritonavir and molnupiravir when pooled separately as HR, OR, or RR were largely similar for each effect measure (Table S6).

## Discussion

This systematic review and meta-analysis of observational studies evaluated the effectiveness of oral antiviral drugs against hospitalization and mortality up to 35 days after infection with SARS-CoV-2 Omicron subvariants in non-hospitalized adults with known COVID-19 vaccination status. Most included studies were judged to be at moderate or serious risk of bias, although after careful combining of similar studies, the pooled estimates were largely consistent with generally low to moderate statistical heterogeneity. The evidence included in this systematic review suggests that the relative effectiveness of nirmatrelvir/ritonavir and molnupiravir is independent of vaccination status.

This is the first systematic review to rigorously synthesize the best available observational evidence for the effectiveness of both nirmatrelvir/ritonavir and molnupiravir and to quantitatively synthesize relative and absolute risks by vaccination status and age group. Because most of the studies included vaccinated adults in the Omicron era, this review provides complementary, precise and highly direct evidence that nirmatrelvir/ritonavir is likely effective in an important patient population that was largely excluded from pivotal placebo-controlled RCTs. The included evidence on the relative and absolute effects for the most important clinical outcomes, and control group risks, can also be invaluable to clinicians and guideline developers to communicate the range of potential benefits of treatment with these oral antiviral drugs when used in populations encountered in typical clinical practice.

Limitations of this study include study-level risk of bias, baseline (clinical) heterogeneity, a limited number of studies reporting estimates for subgroups by vaccination status and age group, and reporting bias. To address the study-level risk of bias, we applied the approach recommended by Cochrane and used the ROBINS-I tool to assess risk of bias and only included estimates from the most robust studies in our meta-analyses. There was a modest degree of baseline heterogeneity amongst the included studies largely as a result of definitions for patient eligibility criteria, vaccination status and outcomes, which we mitigated by clearly defining our research question and pre-specifying inclusion criteria that explicitly considered patient-level vaccination status and time from COVID-19 diagnosis to treatment initiation. Given the low number of studies included in our subgroup analyses, these should be carefully interpreted as there is a possibility for false positive findings, especially for molnupiravir, as a result of low statistical power. Although we conducted an extensive search for unpublished studies in specialized databases and performed an updated literature search before submission, this review may also suffer from reporting biases such as selective outcome reporting, time-lag bias and delays in study publication.^56^ The limited number of studies and lack of beneficial associations for molnupiravir in both randomized and non-randomized studies suggests other unpublished observational studies with non-statistically significant results may exist.^57^

Data from RCTs have provided limited evidence on the magnitude of relative and absolute effectiveness of nirmatrelvir/ritonavir and some evidence for molnupiravir among vaccinated adults. In our study, the magnitude of relative effectiveness of nirmatrelvir/ritonavir and molnupiravir against hospitalization and mortality were attenuated when compared to the results of previous RCTs in unvaccinated populations. The WHO living guideline on COVID-19 therapeutics used data from the EPIC-HR and EPIC-SR trials to estimate the effects of nirmatrelvir/ritonavir among adult outpatients with non-severe COVID-19 and found much larger reductions in mortality (OR 0.04, 95% CI 0.00–0.67) and admission to hospital (OR 0.15, 95% CI 0.06–0.36).^8^ Furthermore, the estimates for molnupiravir were informed from 12 RCTS, and indicated a reduction for mortality (OR 0.21, 95% CI 0.06–0.59) and admission to hospital (OR 0.64, 95% CI 0.39–0.93).^8^ The attenuation in the observed associations in observational studies compared with RCTs could possibly be explained by the longer time to treatment initiation in typical clinical practice (range of 0 to 10 days in the included studies), residual bias from the healthy user effect, since in most studies patients who were prescribed oral antivirals also had higher vaccination rates, suggesting a greater access to care and willingness to seek medical intervention and the heterogeneity in outcome definitions for hospitalization and mortality used across the included studies. However, our pooled RDs for nirmatrelvir/ritonavir against the composite outcome in vaccinated adults are comparable to those reported in the EPIC-SR trial for the high-risk subgroup, defined as vaccinated participants with ≥1 risk factor for severe COVID-19 (1.21% versus 1.29% in EPIC-SR).^10^ RDs for molnupiravir against the composite outcome included the null value indicating potential for benefit or harm, aligning with the PANORAMIC trial that showed no effect of molnupiravir against all-cause hospitalization or mortality among highly vaccinated community-dwelling adults.^13^

Given limitations in the external validity of the existing RCTs on SARS-CoV-2 oral antiviral drugs, observational studies provided real-world evidence of effectiveness in diverse populations and geographical areas. National regulators can use the results of this systematic review to inform their current marketing authorizations based on the totality of the randomized and non-randomized evidence. This is particularly relevant for molnupiravir, as our results corroborate those reported in PANORAMIC. Furthermore, we identified a signal of potential harm with molnupiravir and mortality in vaccinated or younger adults, a finding that requires further research and a reassessment of molnupiravir’s authorization in these subgroups.^58^ The highly anticipated results of the nirmatrelvir/ritonavir arm of PANORAMIC, expected to be released in 2024, will confirm or refute the magnitude of relative effectiveness of nirmatrelvir/ritonavir in our systematic review.^59^ If our estimates align with the findings of the nirmatrelvir/ritonavir arm of PANORAMIC, this would add further evidence that well conducted observational studies may provide valid estimates of treatment effectiveness when the observational study is explicitly designed to emulate a target randomized trial.^60^

### Conclusions

Among non-hospitalized adults with COVID-19, low to moderate certainty evidence from cohort studies supports the effectiveness of nirmatrelvir/ritonavir regardless of vaccination status. Molnupiravir has inconsistent evidence of effectiveness, notably among vaccinated adults, although the available evidence is of very low to moderate certainty.

## Supplementary Material

dkae163_Supplementary_Data

## Data Availability

The data extracted from the included studies and used in the analysis are included in the text and accompanying Supplementary material. Analytic code is available from the corresponding author.

## Supplementary data

Figures S1 to S3 and Tables S1 to S6 are available as Supplementary data at *JAC* Online.
