# Supplementary material for: Effectiveness of nirmatrelvir/ritonavir and molnupiravir in non-hospitalized adults with COVID-19: systematic review and meta-analysis of observational studies
Source: J Antimicrob Chemother. 2024 May 31;79(9):2119–31. doi: 10.1093/jac/dkae163 (PMC11368430; doi:10.1093/jac/dkae163)
Supplement: dkae163_Supplementary_Data [file dkae163_supplementary_data.docx]

**Supplementary Material**

**Effectiveness of nirmatrelvir/ritonavir and molnupiravir for COVID-19 in non-hospitalised adults: systematic review and meta-analysis of observational studies**

**Authors:** Yonatan M Mesfin, Joseph E Blais, Kelemu Tilahun Kibret, Teketo Kassaw Tegegne, Benjamin J Cowling, Peng Wu

**Table S1.** Example of database search strategy for MEDLINE via OVID

Database: Ovid MEDLINE(R) and In-Process, In-Data-Review & Other Non-Indexed Citations 1946 to May 23, 2023

| **#** | **Searches** | **Results** |
| --- | --- | --- |
| 1 | COVID-19.mp. or COVID-19/ [mp=title, book title, abstract, original title, name of substance word, subject heading word, floating sub-heading word, keyword heading word, organism supplementary concept word, protocol supplementary concept word, rare disease supplementary concept word, unique identifier, synonyms, population supplementary concept word, anatomy supplementary concept word] | 330452 |
| 2 | Coronavirus Infections/ or "covid 19 patients".mp. or SARS-CoV-2/ | 180270 |
| 3 | "mild COVID-19".mp. | 1390 |
| 4 | "moderate COVID-19".mp. | 1159 |
| 5 | "coronavirus disease 2019".mp. | 55878 |
| 6 | "antiviral drugs".mp. or Antiviral Agents/ | 103246 |
| 7 | COVID-19 Drug Treatment/ or "COVID-19 treatment".mp. | 12881 |
| 8 | "COVID-19 therapeutics".mp. | 285 |
| 9 | molnupiravir.mp. | 475 |
| 10 | Lagevrio.mp. | 21 |
| 11 | nirmatrelvir.mp. or Ritonavir/ | 5551 |
| 12 | Paxlovid.mp. | 277 |
| 13 | 1 or 2 or 3 or 4 or 5 | 338089 |
| 14 | 6 or 7 or 8 or 9 or 10 or 11 or 12 | 116327 |
| 15 | 13 and 14 | 18225 |
| 16 | Hospitali?ation.mp. or Hospitali?ation/ | 271899 |
| 17 | "Hospital Mortality".mp. or Hospital Mortality/ | 74850 |
| 18 | mortality.mp. or Mortality/ | 1371550 |
| 19 | Death/ or death.mp. | 955619 |
| 20 | "rate of hospitali?ation".mp. | 1791 |
| 21 | "composite disease progression".mp. | 8 |
| 22 | effectiveness.mp. | 573893 |
| 23 | "mechanical ventilation".mp. or Respiration, Artificial/ | 91793 |
| 24 | Critical Care/ or "intensive care admission".mp. | 61038 |
| 25 | "Oxygen Inhalation Therapy".mp. or Oxygen Inhalation Therapy/ | 16029 |
| 26 | "oxygen therapy".mp. | 14123 |
| 27 | 16 or 17 or 18 or 19 or 20 or 21 or 22 or 23 or 24 or 25 or 26 | 2878389 |
| 28 | 15 and 27 | 5590 |
| 29 | limit 28 to (humans and yr="2021 - 2023" and covid-19) | 3619 |
| 30 | limit 29 to (english language and humans and yr="2022 - 2023") | 1685 |

**Table S2.** Outcome definitions used in the original studies and the systematic review and meta-analysis, number of participants and events, and relative and absolute effect estimates for the overall study populations, by age groups, and by vaccination status. Control group risk refers to the cumulative risk during follow-up in the untreated (no oral antiviral) group. WHO 'High Risk' is defined as a ≥ 6% absolute control group risk of hospitalisation as per the WHO COVID-19 living guideline (Version 14).

| **Study** | **Outcome (original study)** | **Outcome definition (original study)** | **Outcome follow-up time (days)** | **Outcome (meta-analysis)** | **Age group (original study)** | **Age group (meta-analysis)** | **Vaccination status (original study)** | **Vaccination status (meta-analysis)** | **Sample size (intervention)** | **Number of events (intervention)** | **Sample size (control)** | **Number of events (control)** | **Relative effect measure** | **Relative effect estimate** | **Relative effect 95% lower bound** | **Relative effect 95% upper bound** | **RD (%)** | **RD (%) 95% lower bound** | **RD (%) 95% upper bound** | **Control group risk (%)** | **WHO 'High Risk'** | **Comment** |
| --- | --- | --- | --- | --- | --- | --- | --- | --- | --- | --- | --- | --- | --- | --- | --- | --- | --- | --- | --- | --- | --- | --- |
| **Nirmatrelvir/ritonavir** | | | | | | | | | | | | | | | | | | | |  |  |  |
| Aggarwal 2023 | All-cause hospitalisation | All-cause hospitalisation that occurred during the follow-up time after the observed or imputed SARS-CoV-2 positive test date | 28 | All-cause hospitalisation | ≥18 | ≥18 | Included as a matching and covariate variable | Considered as a confounder variable | 7168 | 61 | 9361 | 135 | OR | 0.45 | 0.33 | 0.62 | NR | NR | NR | NR | NA | Included in meta-analysis for hospitalisation outcome |
|  | COVID-19 related hospitalisation | Hospitalisation during follow-up time with any of the following: COVID-19 International Classification of Diseases-10 code (U07·1, J12·82, M35·81, Z20·822, or M35·89), administration of inpatient remdesivir, or use of any supplemental oxygen | 28 | NA | ≥18 | NA | Included as a matching and covariate variable | NA | 7168 | 47 | 9361 | 109 | OR | 0.40 | 0.28 | 0.57 | NR | NR | NR | NR | NA | Not included in meta-analysis |
|  | All-cause mortality | All-cause mortality during follow-up time | 28 | All-cause mortality | ≥18 | ≥18 | Included as a matching and covariate variable | Considered as a confounder variable | 7168 | 2 | 9361 | 15 | OR | 0.15 | 0.03 | 0.50 | NR | NR | NR | NR | NA |  |
|  | All-cause hospitalisation | All-cause hospitalisation that occurred during the follow-up time after the observed or imputed SARS-CoV-2 positive test date | 28 | All-cause hospitalisation | ≥18 | ≥18 | Unvaccinated group (0 dose) | Unvaccinated | 1460 | 21 | 2036 | 50 | OR | 0.46 | 0.27 | 0.77 | NR | NR | NR | NR | NA |  |
|  | All-cause hospitalisation | All-cause hospitalisation that occurred during the follow-up time after the observed or imputed SARS-CoV-2 positive test date | 28 | All-cause hospitalisation | ≥18 | ≥18 | First booster or more group (≥3 doses) | Vaccinated | 4354 | 28 | 5395 | 54 | OR | 0.47 | 0.29 | 0.74 | NR | NR | NR | NR | NA |  |
|  | All-cause hospitalisation | All-cause hospitalisation that occurred during the follow-up time after the observed or imputed SARS-CoV-2 positive test date | 28 | All-cause hospitalisation | <65 | 18-64 | Included as a matching and covariate variable | Considered as a confounder variable | 4870 | 32 | 406 | 71 | OR | 0.53 | 0.34 | 0.84 | NR | NR | NR | NR | NA |  |
|  | All-cause hospitalisation | All-cause hospitalisation that occurred during the follow-up time after the observed or imputed SARS-CoV-2 positive test date | 28 | All-cause hospitalisation | ≥65 | ≥65 | Included as a matching and covariate variable | Considered as a confounder variable | 2298 | 29 | 1955 | 64 | OR | 0.37 | 0.23 | 0.57 | NR | NR | NR | NR | NA |  |
| Bajema 2023 | Any hospitalisation or all-cause mortality | Any hospitalisation or all-cause mortality through day 30 after the index date | 30 | All-cause composite outcome | ≥18 | ≥18 | Included as a matching variable | Considered as a confounder variable | 9607 | 221 | 9607 | 328 | RR | 0.67 | 0.58 | 0.79 | 1.117 | 0.703 | 1.530 | 3.42 | No |  |
|  | All-cause hospitalisation | Any hospitalisation through day 30 after the index date | 30 | All-cause hospitalisation | ≥18 | ≥18 | Included as a matching variable | Considered as a confounder variable | 9607 | 212 | 9607 | 291 | RR | 0.73 | 0.62 | 0.85 | 0.825 | 0.423 | 1.227 | 3.03 | No |  |
|  | All-cause mortality | All-cause mortality through day 30 after the index date | 30 | All-cause mortality | ≥18 | ≥18 | Included as a matching variable | Considered as a confounder variable | 9607 | 12 | 9607 | 53 | RR | 0.23 | 0.13 | 0.41 | 0.422 | 0.300 | 0.545 | 0.55 | No |  |
|  | Any hospitalisation or all-cause mortality | Any hospitalisation or all-cause mortality through day 30 after the index date | 30 | All-cause composite outcome | <65 | 18-64 | Included as a matching variable | Considered as a confounder variable | 4481 | 35 | 4409 | 68 | RR | 0.52 | 0.27 | 0.99 | NR | NR | NR | 1.53 | No |  |
|  | Any hospitalisation or all-cause mortality | Any hospitalisation or all-cause mortality through day 30 after the index date | 30 | All-cause composite outcome | ≥65 | ≥65 | Included as a matching variable | Considered as a confounder variable | 5126 | 137 | 5198 | 199 | RR | 0.70 | 0.59 | 0.82 | NR | NR | NR | 3.82 | No |  |
|  | Any hospitalisation or all-cause mortality | Any hospitalisation or all-cause mortality through day 30 after the index date | 30 | All-cause composite outcome | ≥18 | ≥18 | Unvaccinated group (0 dose) | Unvaccinated | 1650 | 50 | 1721 | 52 | RR | 0.93 | 0.66 | 1.31 | 0.224 | -0.838 | 1.285 | 3.23 | No |  |
|  | Any hospitalisation or all-cause mortality | Any hospitalisation or all-cause mortality through day 30 after the index date | 30 | All-cause composite outcome | ≥18 | ≥18 | Primary series completed or boosted group | Vaccinated | 7607 | 157 | 7552 | 240 | RR | 0.65 | 0.54 | 0.78 | 1.127 | 0.665 | 1.589 | 3.18 | No |  |
| Bhatia 2023 | All-cause hospitalisation | Any hospitalisation at any point in time during the 28-day follow-up period | 28 | All-cause hospitalisation | ≥18 | ≥18 | Included as a covariate variable | Considered as a confounder variable | 104510 | NR | 306132 | NR | OR | 0.32 | 0.24 | 0.42 | NR | NR | NR | NR | NA |  |
| Butt 2023 | All-cause hospitalisation or death | Hospitalization or death within 30 days of COVID-19 diagnosis | 30 | All-cause composite outcome | ≥18 | ≥18 | Included as a matching variable | Considered as a confounder variable | 7615 | 243 | 7615 | 444 | HR | 0.54 | 0.46 | 0.63 | 2.640 | 1.980 | 3.300 | 5.87 | No | Using the estimated propensity score, the authors performed both inverse probability of treatment weighting and matching. Data were extracted for the propensity score matched analysis. |
|  | All-cause hospitalisation or death | Hospitalization or death within 30 days of COVID-19 diagnosis | 30 | All-cause composite outcome | ≥18 | ≥18 | Unvaccinated (0 or 1 dose) group | Unvaccinated | 1163 | 37 | 1149 | 906 | HR | 0.51 | 0.34 | 0.76 | 2.300 | 1.280 | 4.720 | 6.18 | Yes |  |
|  | All-cause hospitalisation or death | Hospitalization or death within 30 days of COVID-19 diagnosis | 30 | All-cause composite outcome | ≥18 | ≥18 | Received first booster or more group | Vaccinated | 4995 | 158 | 4988 | 1692 | HR | 0.54 | 0.44 | 0.65 | 2.631 | 1.820 | 3.440 | 5.79 | No |  |
|  | All-cause hospitalisation or death | Hospitalization or death within 30 days of COVID-19 diagnosis | 30 | All-cause composite outcome | ≤60 | 18 to 64 | Included as a matching variable | Considered as a confounder variable | 2511 | 44 | 2437 | 60 | HR | 0.71 | 0.48 | 1.05 | 0.710 | -0.090 | 1.510 | 2.46 | No |  |
|  | All-cause hospitalisation or death | Hospitalization or death within 30 days of COVID-19 diagnosis | 30 | All-cause composite outcome | >60 | ≥65 | Included as a matching variable | Considered as a confounder variable | 5104 | 199 | 5178 | 384 | HR | 0.51 | 0.43 | 0.61 | 3.520 | 2.630 | 4.410 | 7.42 | Yes |  |
| Cegolon 2023 | COVID-19 attributable hospitalisation | Hospitalisation but criteria were not defined in the study | 30 | COVID-19 related hospitalisation | ≥18 | ≥18 | Included as a covariate variable | Considered as a confounder variable | 102 | 2 | 111 | 8 | OR | 0.16 | 0.03 | 0.89 | NR | NR | NR | NR | NA | Number of events was too low to provide an estimate for molnupiravir |
| Dormuth 2023 | COVID-19–related emergency hospital visit or admission, or death from any cause | A COVID-19–related emergency hospital visit or admission, or death from any cause, within 28 days of cohort entry date. COVID-19–related emergency hospitalization was if it indicated COVID-19 infection (International Statistical Classification o fDiseases and Related Health Problems, Tenth Revision, codes U07.1 and U07.2) and subsequent admission to hospital. | 28 | All-cause composite outcome | ≥18 | ≥18 | Included as a covariate variable | Considered as a confounder variable | 1050 | 25 | 1050 | 39 | RR | 0.64 | 0.39 | 1.05 | 1.333 | -0.100 | 2.800 | CEV1: NR CEV2: 3.425 CEV3: 3.714 EXEL: 3.422 | No | Study participants were assigned to one of four cohorts based on their vulnerability to COVID-19 complications. Data were extracted and pooled for cohort 3 (CEV3), defined as individuals with high-risk conditions. Small cell restrictions did not permit reporting of estimates for unvaccinated individuals in the three CEV cohorts or the control group risk for CEV1. |
|  | COVID-19–related emergency hospital visit or admission, or death from any cause | A COVID-19–related emergency hospital visit or admission, or death from any cause, within 28 days of cohort entry date. COVID-19–related emergency hospitalization was if it indicated COVID-19 infection (International Statistical Classification of Diseases and Related Health Problems, Tenth Revision, codes U07.1 and U07.2) and subsequent admission to hospital. | 28 | All-cause composite outcome | ≥18 | ≥18 | Primary series completed or boostered group | Vaccinated | 975 | 24 | 992 | 37 | NR | NR | NR | NR | 1.268 | -0.300 | 2.800 | CEV1: NR CEV2: 3.426 CEV3: 3.730 EXEL: 3.393 | No |  |
| Dryden-Peterson 2023 | All-cause hospitalisation or death | Any hospitalisation between days 2 and 14 or any death between days 2 and 28 after COVID-19 diagnosis | 14 or 28 | All-cause composite outcome | ≥50 | ≥65 | Included as a covariate variable | Considered as a confounder variable | 11797 | 69 | 32248 | 310 | RR | 0.56 | 0.42 | 0.75 | 0.410 | 0.250 | 0.590 | 0.93 | No |  |
|  | All-cause hospitalisation | Any hospitalisation between days 2 and 14 after COVID-19 diagnosis | 14 | All-cause hospitalisation | ≥50 | ≥65 | Included as a covariate variable | Considered as a confounder variable | NR | NR | NR | NR | RR | 0.60 | 0.44 | 0.81 | NR | NR | NR | NR | NA |  |
|  | All-cause death | Any death between days 2 and 28 after COVID-19 diagnosis | 28 | All-cause mortality | ≥50 | ≥65 | Included as a covariate variable | Considered as a confounder variable | NR | NR | NR | NR | RR | 0.29 | 0.12 | 0.71 | NR | NR | NR | NR | NA |  |
|  | All-cause hospitalisation or death | Any hospitalisation between days 2 and 14 or any death between days 2 and 28 after COVID-19 diagnosis | 14 or 28 | All-cause composite outcome | ≥65 | ≥65 | Included as a covariate variable | Considered as a confounder variable | 6656 | 55 | 13079 | 194 | RR | 0.55 | 0.40 | 0.77 | 0.660 | 0.357 | 0.957 | 1.48 | No |  |
|  | All-cause hospitalisation or death | Any hospitalisation between days 2 and 14 or any death between days 2 and 28 after COVID-19 diagnosis | 14 or 28 | All-cause composite outcome | 50-64 | 18-64 | Included as a matching variable | Considered as a confounder variable | 5885 | 16 | 18931 | 93 | RR | 0.55 | 0.30 | 1.03 | 0.220 | 0.053 | 0.385 | 0.49 | No |  |
|  | All-cause hospitalisation or death | Any hospitalisation between days 2 and 14 or any death between days 2 and 28 after COVID-19 diagnosis | 14 or 28 | All-cause composite outcome | ≥50 | ≥65 | Primary series completed or boostered group | Vaccinated | 11859 | 63 | 28377 | 219 | RR | 0.69 | 0.50 | 0.94 | 0.240 | 0.075 | 0.406 | 0.77 | No |  |
|  | All-cause hospitalisation or death | Any hospitalisation between days 2 and 14 or any death between days 2 and 28 after COVID-19 diagnosis | 14 or 28 | All-cause composite outcome | ≥50 | ≥65 | Unvaccinated/partially vaccinated | Unvaccinated | 682 | 3 | 3633 | 90 | RR | 0.19 | 0.08 | 0.49 | 2.000 | 1.329 | 2.746 | 2.48 | No |  |
| Evans 2023 | All-cause admission to hospital or death | Any cause hospitalisation or death (if death occurred without prior admission) within 28 days of a positive COVID-19 test | 28 | All-cause composite outcome | ≥18 | ≥18 | Included as a covariate variable | Considered as a confounder variable | 602 | 17 | 4973 | 544 | HR | 0.59 | 0.36 | 0.97 | NR | NR | NR | NR | NA |  |
| Faust 2023 | All-cause hospitalisation | All-cause hospitalisation within 30 days of diagnosis of COVID-19 | 30 | NA | 18 to 50 | NA | Primary series completed or boostered group | NA | 2547 | 15 | 2547 | 43 | OR | 0.35 | 0.19 | 0.62 | 1.100 | 0.500 | 1.700 | 1.69 | No | Not pooled since it is a subgroup analysis of younger patients included in Ganatra 2023 |
|  | All-cause mortality | All-cause mortality within 30 days of diagnosis of COVID-19 | 30 | NA | 18 to 50 | NA | Primary series completed or boostered group | NA | 2547 | 0 | 2547 | 10 | OR | NR | NR | NR | 0.400 | 0.100 | 0.600 | 0.39 | No |  |
|  | All-cause emergency department visits, hospitalization, or death | All-cause emergency department visits, hospitalization, or death within 30 days of diagnosis of COVID-19 | 30 | NA | 18 to 50 | NA | Primary series completed or boostered group | NA | 2547 | 125 | 2547 | 179 | OR | 0.68 | 0.54 | 0.86 | 2.130 | 0.800 | 3.400 | 7.03 | Yes |  |
| Ganatra 2023 | All-cause hospitalisation | All-cause hospitalisation within 30 days of diagnosis of COVID-19 | 30 | All-cause hospitalisation | ≥18 | ≥18 | Primary series completed or boostered group | Vaccinated | 1130 | 10 | 1130 | 23 | OR | 0.43 | 0.20 | 0.91 | 1.200 | 0.200 | 2.100 | 2.00 | No |  |
|  | All-cause mortality | All-cause mortality within 30 days of diagnosis of COVID-19 | 30 | All-cause mortality | ≥18 | ≥18 | Primary series completed or boostered group | Vaccinated | 1130 | 0 | 1130 | 10 | OR | NR | NR | NR | 0.900 | 0.300 | 1.400 | 0.80 | No |  |
|  | All-cause emergency department visits, hospitalization, or death | All-cause emergency department visits, hospitalization, or death within 30 days of diagnosis of COVID-19 | 30 | NA | ≥18 | NA | Primary series completed or boostered group | NA | 1130 | 89 | 1130 | 163 | OR | 0.51 | 0.39 | 0.67 | 6.500 | 40.000 | 9.100 | 14.40 | Yes | Composite outcome primarily driven by emergency department visits, which had a control group risk of 12.5%. Therefore, the composite outcome was judeged to be sufficiently differerent from other studies so it was not pooled. |
| Kwok 2023 | COVID-19 related hospitalization | Defined by admission to an acute medical ward for management of COVID-19 infection (for more than 24 h) within 14 days of confirmed COVID-19 infection | 14 | NA | ≥18 | NA | Enrolled only unvaccinated patients | NA | 302 | 15 | 2387 | 228 | RR | 0.56 | 0.31 | 1.02 | NR | NR | NR | NR | NA | Not pooled since judged to be at critical risk of bias |
|  | COVID-19 related mortality | Defined as inpatient death during COVID-19 related hospitalization within 14 days of confirmed COVID-19 infection | 14 | NA | ≥18 | NA | Enrolled only unvaccinated patients | NA | NR | 4 | NR | 120 | RR | 0.37 | 0.14 | 1.01 | NR | NR | NR | NR | NA |  |
| Lewnard 2023 | All-cause hospital admission or death | Hospital admission or death from any cause within 30 days of the index positive SARS-CoV-2 test | 30 | All-cause composite outcome | ≥12 | ≥18 | Included as a matching variable | Considered as a confounder variable | 7274 | 46 | 126152 | 641 | HR | 0.20 | 0.06 | 0.66 | NR | NR | NR | NR | NA |  |
| Liu 2023 | All-cause hospitalisation | All-cause hospitalisation within follow-up time | 10-30 | NA | ≥18 | NA | Unvaccinated (0 or 1 dose) group | NA | NR | 209 | NR | 391 | HR | 0.53 | 0.45 | 0.63 | NR | NR | NR | NR | NA | Not pooled since judged to be at critical risk of bias |
|  | All-cause hospitalisation | All-cause hospitalisation within follow-up time | 10-30 | NA | ≥18 | NA | Primary series completed or boostered group | NA | NR | 62 | NR | 50 | HR | 1.27 | 0.87 | 1.85 | NR | NR | NR | NR | NA |  |
|  | All-cause mortality | All-cause mortality within follow-up time | 10-30 | NA | ≥18 | NA | Unvaccinated (0 or 1 dose) group | NA | NR | 10 | NR | 17 | HR | 0.17 | 0.05 | 0.61 | NR | NR | NR | NR | NA |  |
|  | All-cause hospitalisation or all-cause mortality | All-cause hospitalisation or all-cause mortality within follow-up time | 10-30 | NA | ≥18 | NA | Unvaccinated (0 or 1 dose) group | NA | NR | 209 | NR | 406 | HR | 0.51 | 0.44 | 0.61 | NR | NR | NR | NR | NA |  |
|  | All-cause hospitalisation or all-cause mortality | All-cause hospitalisation or all-cause mortality within follow-up time | 10-30 | NA | ≥18 | NA | Primary series completed or boostered group | NA | NR | 63 | NR | 50 | HR | 1.29 | 0.89 | 1.87 | NR | NR | NR | NR | NA |  |
| Low 2023 | COVID-19 related hospitalisation | COVID-19 related hospitalisation in Malaysia’s MySejahtera eCOVID system | 30 | COVID-19 related hospitalisation | ≥18 | ≥18 | Included as a matching variable | Considered as a confounder variable | 10483 | 37 | 10483 | 55 | HR | 0.67 | 0.44 | 1.02 | NR | NR | NR | NR | NA | Number of events was too low to estimate effects in the partially vaccinated subgroup |
|  | COVID-19 related hospitalisation | COVID-19 related hospitalisation in Malaysia’s MySejahtera eCOVID system | 30 | COVID-19 related hospitalisation | 18 to 40 | 18 to 64 | Included as a matching variable | Considered as a confounder variable | 3678 | 9 | 4022 | 27 | HR | 0.44 | 0.20 | 0.93 | NR | NR | NR | NR | NA |  |
|  | COVID-19 related hospitalisation | COVID-19 related hospitalisation in Malaysia’s MySejahtera eCOVID system | 30 | COVID-19 related hospitalisation | ≥60 | ≥65 | Included as a matching variable | Considered as a confounder variable | 3186 | 23 | 2714 | 22 | HR | 0.89 | 0.50 | 1.60 | NR | NR | NR | NR | NA |  |
|  | COVID-19 related hospitalisation | COVID-19 related hospitalisation in Malaysia’s MySejahtera eCOVID system | 30 | COVID-19 related hospitalisation | ≥18 | ≥18 | Included as a matching variable | Vaccinated | NR | NR | NR | NR | HR | 0.67 | 0.43 | 1.04 | NR | NR | NR | NR | NA |  |
| Lui 2023 | All-cause mortality or all-cause hospitalisation | All-cause mortality or all-cause hospitalisation at any time during follow-up | 30 | NA | ≥18 | NA | Included as a matching variable | NA | 793 | NR | 793 | NR | HR | 0.71 | 0.63 | 0.80 | NR | NR | NR | NR | NA | Not pooled because it is a subgroup analysis of patients with type 2 diabetes mellitus from a larger study (Wong 2023) |
|  | All-cause hospitalisation | All-cause hospitalisation at any time during follow-up | 30 | NA | ≥18 | NA | Included as a matching variable | NA | 793 | NR | 793 | NR | HR | 0.71 | 0.63 | 0.80 | NR | NR | NR | NR | NA |  |
|  | All-cause mortality | All-cause mortality at any time during follow-up | 30 | NA | ≥18 | NA | Included as a matching variable | NA | 793 | NR | 793 | NR | HR | 0.29 | 0.13 | 0.63 | NR | NR | NR | NR | NA |  |
|  | All-cause mortality or all-cause hospitalisation | All-cause mortality or all-cause hospitalisation at any time during follow-up | 30 | NA | ≥18 | NA | Unvaccinated (0 or 1 dose) group | NA | 321 | 143 | 321 | 204 | HR | 0.61 | 0.51 | 0.72 | NR | NR | NR | NR | NA |  |
| Najjar-Debbiny 2023 | Severe COVID-19 or COVID-19-specific mortality | Severe COVID-19 defined as an oxygen saturation <94% on room air, a ratio of arterial partial pressure of oxygen to fraction of inspired oxygen <300 mm Hg, or a respiratory rate >30 breaths/min. COVID-19 specific mortality was not defined. | 28 | COVID-19 related composite outcome | ≥18 | ≥18 | Included as a covariate variable | Considered as a confounder variable | 4737 | 39 | 175614 | 903 | HR | 0.54 | 0.39 | 0.75 | NR | NR | NR | NR | NA |  |
|  | Severe COVID-19 or COVID-19-specific mortality | Severe COVID-19 defined as an oxygen saturation <94% on room air, a ratio of arterial partial pressure of oxygen to fraction of inspired oxygen <300 mm Hg, or a respiratory rate >30 breaths/min. COVID-19 specific mortality was not defined. | 28 | COVID-19 related composite outcome | <60 | 18-64 | Included as a covariate variable | Considered as a confounder variable | 973 | NR | 102040 | NR | HR | 1.06 | 0.36 | 3.15 | NR | NR | NR | NR | NA |  |
|  | Severe COVID-19 or COVID-19-specific mortality | Severe COVID-19 defined as an oxygen saturation <94% on room air, a ratio of arterial partial pressure of oxygen to fraction of inspired oxygen <300 mm Hg, or a respiratory rate >30 breaths/min. COVID-19 specific mortality was not defined. | 28 | COVID-19 related composite outcome | ≥60 | ≥65 | Included as a covariate variable | Considered as a confounder variable | 3764 | NR | 73574 | NR | HR | 0.52 | 0.36 | 0.73 | NR | NR | NR | NR | NA |  |
|  | Severe COVID-19 or COVID-19-specific mortality | Severe COVID-19 defined as an oxygen saturation <94% on room air, a ratio of arterial partial pressure of oxygen to fraction of inspired oxygen <300 mm Hg, or a respiratory rate >30 breaths/min. COVID-19 specific mortality was not defined. | 28 | COVID-19 related composite outcome | ≥18 | ≥18 | Unvaccinated (0 or 1 dose) group | Unvaccinated | 1051 | NR | 43818 | NR | HR | 0.52 | 0.32 | 0.82 | NR | NR | NR | NR | NA |  |
|  | Severe COVID-19 or COVID-19-specific mortality | Severe COVID-19 defined as an oxygen saturation <94% on room air, a ratio of arterial partial pressure of oxygen to fraction of inspired oxygen <300 mm Hg, or a respiratory rate >30 breaths/min. COVID-19 specific mortality was not defined. | 28 | COVID-19 related composite outcome | ≥18 | ≥18 | Primary series completed or boostered group | Vaccinated | 3686 | NR | 131796 | NR | HR | 0.62 | 0.39 | 0.98 | NR | NR | NR | NR | NA |  |
| Paraskevis 2023 | Hospitalization for COVID-19 | Hospitalization for COVID-19 within 10 days after a positive SARS-CoV-2 test result with no ICU admission or clinical deterioration (intubation) | 10 | NA | ≥65 | NA | Included as a covariate variable | NA | 13861 | 297 | 13861 | 857 | OR | 0.31 | 0.27 | 0.36 | NR | NR | NR | NR | NA | Not pooled since judged to be at critical risk of bias |
| Petrakis 2023 | Hospitalisation or intubation or mortality | Composite of hospitalisation, intubation, death within 30 days after a positive SARS-CoV-2 test | 30 | NA | ≥18 | NA | Included as a covariate variable | NA | 200 | 3 | 200 | 120 | OR | 0.34 | 0.29 | 0.55 | NR | NR | NR | NR | NA | Not pooled since judged to be at critical risk of bias |
| Schwartz 2023 | All-cause death | Mortality 1–30 days after the index date | 30 | All-cause mortality | ≥18 | ≥18 | Included as a covariate variable | Considered as a confounder variable | 8876 | 142 | 168669 | 5566 | OR | 0.49 | 0.40 | 0.60 | 1.667 | 1.299 | 2.273 | 3.30 | No |  |
|  | Composite of hospital admission because of COVID-19 or all-cause death | Hospital admission because of COVID-19 or all-cause death that occurred 1–30 days after the index date. Local public health units for public health purposes defines hospital admissions related to COVID-19 for people who received treatment for COVID-19 while in hospital or if their length of stay was extended because of COVID-19. | 30 | All-cause composite outcome | ≥18 | ≥18 | Included as a covariate variable | Considered as a confounder variable | 8876 | 186 | 168669 | 6241 | OR | 0.56 | 0.47 | 0.67 | 1.600 | 1.250 | 2.330 | 3.71 | No |  |
|  | Composite of hospital admission because of COVID-19 or all-cause death | Hospital admission because of COVID-19 or all-cause death that occurred 1–30 days after the index date. Local public health units for public health purposes defines hospital admissions related to COVID-19 for people who received treatment for COVID-19 while in hospital or if their length of stay was extended because of COVID-19. | 30 | All-cause composite outcome | <70 | 18-64 | Included as a covariate variable | Considered as a confounder variable | 2443 | 7 | 129647 | 1037 | OR | 0.34 | 0.15 | 0.79 | NR | NR | NR | 0.80 | No |  |
|  | Composite of hospital admission because of COVID-19 or all-cause death | Hospital admission because of COVID-19 or all-cause death that occurred 1–30 days after the index date. Local public health units for public health purposes defines hospital admissions related to COVID-19 for people who received treatment for COVID-19 while in hospital or if their length of stay was extended because of COVID-19. | 30 | All-cause composite outcome | ≥70 | ≥65 | Included as a covariate variable | Considered as a confounder variable | 6433 | 180 | 39022 | 1951 | OR | 0.55 | 0.45 | 0.66 | NR | NR | NR | 5.00 | No |  |
|  | All-cause death | Mortality 1–30 days after the index date | 30 | All-cause mortality | <70 | 18-64 | Included as a covariate variable | Considered as a confounder variable | 2443 | 3 | 129647 | 778 | OR | 0.13 | 0.03 | 0.13 | 0.535 | 0.338 | 1.299 | 0.60 | No |  |
|  | All-cause death | Mortality 1–30 days after the index date | 30 | All-cause mortality | ≥70 | ≥65 | Included as a covariate variable | Considered as a confounder variable | 6433 | 142 | 39022 | 1756 | OR | 0.48 | 0.39 | 0.59 | 2.273 | 1.754 | 3.125 | 4.50 | No |  |
|  | Composite of hospital admission because of COVID-19 or all-cause death | Hospital admission because of COVID-19 or all-cause death that occurred 1–30 days after the index date. Local public health units for public health purposes defines hospital admissions related to COVID-19 for people who received treatment for COVID-19 while in hospital or if their length of stay was extended because of COVID-19. | 30 | All-cause composite outcome | ≥18 | ≥18 | Unvaccinated (0 dose) group | Unvaccinated | 467 | 14 | 10434 | 689 | OR | 0.44 | 0.23 | 0.84 | 3.600 | 2.040 | 14.286 | 6.60 | Yes |  |
|  | Composite of hospital admission because of COVID-19 or all-cause death | Hospital admission because of COVID-19 or all-cause death that occurred 1–30 days after the index date. Local public health units for public health purposes defines hospital admissions related to COVID-19 for people who received treatment for COVID-19 while in hospital or if their length of stay was extended because of COVID-19. | 30 | All-cause composite outcome | ≥18 | ≥18 | Received first booster or more group | Vaccinated | 7524 | 166 | 127906 | 4477 | OR | 0.62 | 0.51 | 0.75 | 1.300 | 0.926 | 2.174 | 3.50 | No |  |
|  | All-cause death | Mortality 1–30 days after the index date | 30 | All-cause mortality | ≥18 | ≥18 | Unvaccinated (0 dose) group | Unvaccinated | 467 | 9 | 10434 | 574 | OR | 0.34 | 0.16 | 0.74 | 3.571 | 2.083 | 11.111 | 5.50 | No |  |
|  | All-cause death | Mortality 1–30 days after the index date | 30 | All-cause mortality | ≥18 | ≥18 | Received first booster or more group | Vaccinated | 7524 | 128 | 127906 | 3965 | OR | 0.54 | 0.43 | 0.67 | 1.389 | 1.031 | 2.128 | 3.10 | No |  |
| Shah 2023 | COVID-19 hospitalization | Defined as overnight hospitalization during the 30 days after the date of diagnosis and having a COVID-19–specific diagnosis code (ICD-10 U07.1 or SNOMED-CT 840539006) associated with the admission | 30 | COVID-19 related hospitalisation | ≥18 | ≥18 | Included as a covariate variable | Considered as a confounder variable | 198927 | 948 | 500921 | 4366 | HR | 0.49 | 0.46 | 0.53 | NR | NR | NR | NR | NA |  |
|  | COVID-19 hospitalization | Defined as overnight hospitalization during the 30 days after the date of diagnosis and having a COVID-19–specific diagnosis code (ICD-10 U07.1 or SNOMED-CT 840539006) associated with the admission | 30 | COVID-19 related hospitalisation | ≥18 | ≥18 | Received first booster or more group | Vaccinated | 119324 | 512 | 209614 | 1753 | HR | 0.50 | 0.45 | 0.55 | NR | NR | NR | NR | NA |  |
|  | COVID-19 hospitalization | Defined as overnight hospitalization during the 30 days after the date of diagnosis and having a COVID-19–specific diagnosis code (ICD-10 U07.1 or SNOMED-CT 840539006) associated with the admission | 30 | COVID-19 related hospitalisation | ≥18 | ≥18 | Unvaccinated (0 dose) group | Unvaccinated | 30619 | 180 | 141931 | 1323 | HR | 0.50 | 0.43 | 0.59 | NR | NR | NR | NR | NA |  |
|  | COVID-19 hospitalization | Defined as overnight hospitalization during the 30 days after the date of diagnosis and having a COVID-19–specific diagnosis code (ICD-10 U07.1 or SNOMED-CT 840539006) associated with the admission | 30 | COVID-19 related hospitalisation | ≥65 | ≥65 | Included as a covariate variable | Considered as a confounder variable | 75378 | 672 | 132558 | 2736 | HR | 0.53 | 0.48 | 0.58 | NR | NR | NR | NR | NA |  |
|  | COVID-19 hospitalization | Defined as overnight hospitalization during the 30 days after the date of diagnosis and having a COVID-19–specific diagnosis code (ICD-10 U07.1 or SNOMED-CT 840539006) associated with the admission | 30 | COVID-19 related hospitalisation | 18 to 49 | 18-64 | Included as a covariate variable | Considered as a confounder variable | 56620 | 119 | 221089 | 775 | HR | 0.59 | 0.48 | 0.71 | NR | NR | NR | NR | NA |  |
|  | COVID-19 hospitalization | Defined as overnight hospitalization during the 30 days after the date of diagnosis and having a COVID-19–specific diagnosis code (ICD-10 U07.1 or SNOMED-CT 840539006) associated with the admission | 30 | COVID-19 related hospitalisation | ≥65 | ≥65 | First booster or more group | Vaccinated | NR | NR | NR | NR | HR | 0.51 | 0.46 | 0.57 | NR | NR | NR | NR | NA |  |
|  | COVID-19 hospitalization | Defined as overnight hospitalization during the 30 days after the date of diagnosis and having a COVID-19–specific diagnosis code (ICD-10 U07.1 or SNOMED-CT 840539006) associated with the admission | 30 | COVID-19 related hospitalisation | ≥65 | ≥65 | Unvaccinated (0 dose) group | Unvaccinated | NR | NR | NR | NR | HR | 0.58 | 0.47 | 0.72 | NR | NR | NR | NR | NA |  |
|  | COVID-19 hospitalization | Defined as overnight hospitalization during the 30 days after the date of diagnosis and having a COVID-19–specific diagnosis code (ICD-10 U07.1 or SNOMED-CT 840539006) associated with the admission | 30 | COVID-19 related hospitalisation | 18 to 49 | 18-64 | Received first booster or more group | Vaccinated | NR | NR | NR | NR | HR | 0.75 | 0.53 | 1.06 | NR | NR | NR | NR | NA |  |
|  | COVID-19 hospitalization | Defined as overnight hospitalization during the 30 days after the date of diagnosis and having a COVID-19–specific diagnosis code (ICD-10 U07.1 or SNOMED-CT 840539006) associated with the admission | 30 | COVID-19 related hospitalisation | 18 to 49 | 18-64 | Unvaccinated (0 dose) group | Unvaccinated | NR | NR | NR | NR | HR | 0.54 | 0.39 | 0.76 | NR | NR | NR | NR | NA |  |
| Van Heer 2023 | Hospitalisation due to any cause | Defined through a VICNISS flag in the case database, TREVI, which involved hospital clinicians reporting all COVID-19 cases admitted to hospital during their infectious period, defined as 7 days following an initial positive COVID-19 PCR or RAT, or assessed as infectious (‘activeCOVID-19’), regardless of the reason for admission | 35 | All-cause hospitalisation | ≥70 | ≥65 | Included as a covariate variable | Considered as a confounder variable | 4823 | 46 | 10637 | 185 | OR | 0.60 | 0.43 | 0.83 | NR | NR | NR | NR | NA |  |
|  | COVID-19 associated mortality | Defined as per the Victorian DH surveillance definition—COVID-19 listed as a primary or contributing cause of death on the medical death certificate, or a death within 35 days of diagnosis, excluding trauma/accidents and suicide | 35 | COVID-19 related mortality | ≥70 | ≥65 | Included as a covariate variable | Considered as a confounder variable | 5250 | 29 | 13721 | 462 | OR | 0.27 | 0.17 | 0.40 | NR | NR | NR | NR | NA |  |
| Wee 2023 | COVID-19-related hospitalization | Defined as all-cause hospitalisation occurring within 30 days from a positive COVID-19 result | 30 | All-cause hospitalisation | ≥60 | ≥65 | Included as a covariate variable | Considered as a confounder variable | 3959 | 59 | 139379 | 2323 | OR | 0.65 | 0.50 | 0.85 | NR | NR | NR | NR | NA |  |
|  | Progression to severe COVID-19 | Severe COVID-19 was defined as oxygen requirement, intensive care unit admission, or death | 30 | All-cause composite outcome | ≥60 | ≥65 | Included as a covariate variable | Considered as a confounder variable | 3959 | NR | 139379 | NR | OR | 0.86 | 0.48 | 1.55 | NR | NR | NR | NR | NA |  |
| Wong 2022 | All-cause mortality | Defined as patients who died within 28 days of confirmed SARS-CoV-2 infection | 28 | All-cause mortality | ≥18 | ≥18 | Included as a matching variable | Considered as a confounder variable | 5542 | NR | 54672 | NR | HR | 0·34 | 0.22 | 0.52 | NR | NR | NR | NR | NA |  |
|  | All-cause mortality | Defined as patients who died within 28 days of confirmed SARS-CoV-2 infection | 28 | All-cause mortality | ≥18 | ≥18 | Unvaccinated (0 or 1 dose) group | Unvaccinated | 3692 | NR | 36534 | NR | HR | 0.44 | 0.30 | 0.66 | NR | NR | NR | NR | NA |  |
|  | All-cause mortality | Defined as patients who died within 28 days of confirmed SARS-CoV-2 infection | 28 | All-cause mortality | >60 | ≥65 | Included as a matching variable | Considered as a confounder variable | 4758 | NR | 46601 | NR | HR | 0.48 | 0.32 | 0.74 | NR | NR | NR | NR | NA |  |
|  | Hospital admission due to COVID-19 | Defined as patients admitted to hospital as a result of COVID-19 within 28 days of confirmed SARS-CoV-2 infection (no diagnosis codes were described) | 28 | COVID-19 related hospitalisation | ≥18 | ≥18 | Included as a matching variable | Considered as a confounder variable | 5542 | NR | 54672 | NR | HR | 0.76 | 0.67 | 0.86 | NR | NR | NR | NR | NA |  |
|  | Hospital admission due to COVID-19 | Defined as patients admitted to hospital as a result of COVID-19 within 28 days of confirmed SARS-CoV-2 infection (no diagnosis codes were described) | 28 | COVID-19 related hospitalisation | ≥18 | ≥18 | Primary series completed or boostered group | Vaccinated | 1850 | NR | 18138 | NR | HR | 0.71 | 0.51 | 1.01 | NR | NR | NR | NR | NA |  |
|  | Hospital admission due to COVID-19 | Defined as patients admitted to hospital as a result of COVID-19 within 28 days of confirmed SARS-CoV-2 infection (no diagnosis codes were described) | 28 | COVID-19 related hospitalisation | ≥18 | ≥18 | Unvaccinated (0 or 1 dose) group | Unvaccinated | 3692 | NR | 36534 | NR | HR | 0.76 | 0.66 | 0.87 | NR | NR | NR | NR | NA |  |
|  | Hospital admission due to COVID-19 | Defined as patients admitted to hospital as a result of COVID-19 within 28 days of confirmed SARS-CoV-2 infection (no diagnosis codes were described) | 28 | COVID-19 related hospitalisation | ≤60 | 18-64 | Included as a matching variable | Considered as a confounder variable | 784 | NR | 8071 | NR | HR | 0.50 | 0.31 | 0.81 | NR | NR | NR | NR | NA |  |
|  | Hospital admission due to COVID-19 | Defined as patients admitted to hospital as a result of COVID-19 within 28 days of confirmed SARS-CoV-2 infection (no diagnosis codes were described) | 28 | COVID-19 related hospitalisation | >60 | ≥65 | Included as a matching variable | Considered as a confounder variable | 4758 | NR | 46601 | NR | HR | 0.80 | 0.69 | 0.91 | NR | NR | NR | NR | NA |  |
|  | In-hospital disease progression | Defined as composite of in-hospital mortality, invasive mechanical ventilation, or intensive care unit admission | 28 | All-cause composite outcome | ≥18 | ≥18 | Included as a matching variable | Considered as a confounder variable | 5542 | NR | 54672 | NR | HR | 0.57 | 0.38 | 0.87 | NR | NR | NR | NR | NA |  |
|  | In-hospital disease progression | Defined as composite of in-hospital mortality, invasive mechanical ventilation, or intensive care unit admission | 28 | All-cause composite outcome | ≥18 | ≥18 | Primary series completed or boostered group | Vaccinated | 1850 | NR | 18138 | NR | HR | 0.58 | 0.14 | 2.41 | NR | NR | NR | NR | NA |  |
|  | In-hospital disease progression | Defined as composite of in-hospital mortality, invasive mechanical ventilation, or intensive care unit admission | 28 | All-cause composite outcome | ≥18 | ≥18 | Unvaccinated (0 or 1 dose) group | Unvaccinated | 3692 | NR | 36534 | NR | HR | 0.73 | 0.49 | 1.09 | NR | NR | NR | NR | NA |  |
|  | In-hospital disease progression | Defined as composite of in-hospital mortality, invasive mechanical ventilation, or intensive care unit admission | 28 | All-cause composite outcome | >60 | ≥65 | Included as a matching variable | Considered as a confounder variable | 5542 | NR | 54672 | NR | HR | 0.71 | 0.47 | 1.06 | NR | NR | NR | NR | NA | Limited number of events did not permit estimation of the effect in age ≤60 years subgroup |
| Wu 2023 | All-cause hospitalisation and death | All-cause hospitalisation or all-cause death within follow-up time | 10-30 | NA | ≥18 | NA | Unvaccinated (0 dose) group | NA | NR | NR | NR | NR | HR | 0.46 | 0.36 | 0.59 | NR | NR | NR | NR | NA | Not pooled since judged to be at critical risk of bias |
|  | All-cause hospitalisation and death | All-cause hospitalisation or all-cause death within follow-up time | 10-30 | NA | ≥18 | NA | First booster or more group (≥3 doses) | NA | NR | NR | NR | NR | HR | 0.79 | 0.56 | 1.12 | NR | NR | NR | NR | NA |  |
|  | All-cause hospitalisation | All-cause hospitalisation within follow-up time | 10-30 | NA | ≥18 | NA | Unvaccinated (0 dose) group | NA | NR | NR | NR | NR | HR | 0.59 | 0.45 | 0.77 | NR | NR | NR | NR | NA |  |
|  | All-cause hospitalisation | All-cause hospitalisation within follow-up time | 10-30 | NA | ≥18 | NA | First booster or more group (≥3 doses) | NA | NR | NR | NR | NR | HR | 0.79 | 0.56 | 1.12 | NR | NR | NR | NR | NA |  |
|  | All-cause death | All-cause mortality within follow-up time | 10-30 | NA | ≥18 | NA | Unvaccinated (0 dose) group | NA | NR | NR | NR | NR | HR | 0.08 | 0.03 | 0.24 | NR | NR | NR | NR | NA |  |
|  | All-cause hospitalisation and death | All-cause hospitalisation or all-cause death within follow-up time | 10-30 | NA | ≥18 | NA | Primary series completed or boostered group | NA | 458 | 240 | 458 | 338 | HR | 0.62 | 0.52 | 0.73 | NR | NR | NR | NR | NA |  |
| Xie 2023 | Admission to hospital or death | Hospital admission based on information from the inpatient database and death based on patient vital status data | 30 | All-cause composite outcome | ≥18 | ≥18 | Unvaccinated (0 dose) group | Unvaccinated | 5338 | NR | 71425 | NR | RR | 0.60 | 0.50 | 0.71 | 1.830 | 1.290 | 2.490 | 4.65 | No | Conducted five target trial emulations of nirmatrelvir/ritonavir among patients with different baseline vaccination and prior infection statuses. Relative and absolute effect estimates for the unvaccinated (0 dose trial) and vaccinated (≥ 3 doses booster trial) study populations were extracted and were only pooled in the analysis by vaccination status. |
|  | Admission to hospital or death | Hospital admission based on information from the inpatient database and death based on patient vital status data | 30 | All-cause composite outcome | ≥18 | ≥18 | Received first booster or more group | Vaccinated | 18197 | NR | 76708 | NR | RR | 0.64 | 0.58 | 0.71 | 1.050 | 0.850 | 1.270 | 2.94 | No |  |
|  | Admission to hospital or death | Hospital admission based on information from the inpatient database and death based on patient vital status data | 30 | All-cause composite outcome | ≤65 | 18-64 | Included as a covariate variable | Considered as a confounder variable | NR | NR | NR | NR | RR | 0.63 | 0.54 | 0.72 | 0.920 | 0.690 | 1.210 | 2.50 | No |  |
|  | Admission to hospital or death | Hospital admission based on information from the inpatient database and death based on patient vital status data | 30 | All-cause composite outcome | >65 | ≥65 | Included as a covariate variable | Considered as a confounder variable | NR | NR | NR | NR | RR | 0.61 | 0.56 | 0.66 | 1.550 | 1.280 | 1.790 | 3.94 | No |  |
| **Molnupiravir** | | | | | | | | | | | | | | | | |  |  |  |  |  |  |
| Bajema 2023 | Any hospitalisation or all-cause mortality | Any hospitalisation or all-cause mortality through day 30 after the index date | 30 | All-cause composite outcome | ≥18 | ≥18 | Included as a matching variable | Considered as a confounder variable | 3504 | 153 | 3504 | 187 | RR | 0.82 | 0.68 | 0.98 | 0.971 | 0.137 | 1.804 | 5.34 | No |  |
|  | All-cause hospitalisation | Any hospitalisation through day 30 after the index date | 30 | All-cause hospitalisation | ≥18 | ≥18 | Included as a matching variable | Considered as a confounder variable | 3504 | 146 | 3504 | 150 | RR | 0.98 | 0.81 | 1.18 | 0.100 | -0.705 | 0.905 | 4.27 | No |  |
|  | All-cause mortality | All-cause mortality through day 30 after the index date | 30 | All-cause mortality | ≥18 | ≥18 | Included as a matching variable | Considered as a confounder variable | 3504 | 11 | 3504 | 48 | RR | 0.23 | 0.13 | 0.43 | 1.042 | 0.735 | 1.349 | 1.36 | No |  |
|  | Any hospitalisation or all-cause mortality | Any hospitalisation or all-cause mortality through day 30 after the index date | 30 | All-cause composite outcome | <65 | 18-64 | Included as a matching variable | Considered as a confounder variable | 1213 | 5 | 1198 | 18 | RR | 0.33 | 0.04 | 2.92 | NR | NR | NR | 1.15 | No |  |
|  | Any hospitalisation or all-cause mortality | Any hospitalisation or all-cause mortality through day 30 after the index date | 30 | All-cause composite outcome | ≥65 | ≥65 | Included as a matching variable | Considered as a confounder variable | 2291 | 111 | 2306 | 132 | RR | 0.85 | 0.71 | 1.02 | NR | NR | NR | 5.72 | No |  |
|  | Any hospitalisation or all-cause mortality | Any hospitalisation or all-cause mortality through day 30 after the index date | 30 | All-cause composite outcome | ≥18 | ≥18 | Unvaccinated (0 dose) group | Unvaccinated | 503 | 24 | 533 | 44 | RR | 0.59 | 0.37 | 0.93 | 3.432 | 0.650 | 6.215 | 8.28 | Yes |  |
|  | Any hospitalisation or all-cause mortality | Any hospitalisation or all-cause mortality through day 30 after the index date | 30 | All-cause composite outcome | ≥18 | ≥18 | Primary series completed or received boosters | Vaccinated | 2881 | 116 | 2825 | 126 | RR | 0.90 | 0.73 | 1.13 | 0.423 | -0.481 | 1.328 | 4.45 | No |  |
| Butt 2023 | All-cause hospitalisation or death | Hospitalization or death within 30 days of COVID-19 diagnosis | 30 | All-cause composite outcome | ≥18 | ≥18 | Included as a matching variable | Considered as a confounder variable | 1459 | 48 | 1459 | 44 | NR | NR | NR | NR | -0.274 | -1.490 | 0.940 | 3.02 | No |  |
|  | All-cause hospitalisation or death | Hospitalization or death within 30 days of COVID-19 diagnosis | 30 | All-cause composite outcome | ≥18 | ≥18 | Unvaccinated/partially vaccinated | Unvaccinated | 207 | 10 | 207 | 9 | NR | NR | NR | NR | -0.480 | -4.390 | 3.420 | 4.35 | No |  |
|  | All-cause hospitalisation or death | Hospitalization or death within 30 days of COVID-19 diagnosis | 30 | All-cause composite outcome | ≥18 | ≥18 | Received first booster or more group | Vaccinated | 931 | 31 | 931 | 24 | NR | NR | NR | NR | -0.752 | -2.230 | 0.720 | 2.58 | No |  |
|  | All-cause hospitalisation or death | Hospitalization or death within 30 days of COVID-19 diagnosis | 30 | All-cause composite outcome | ≤60 | 18 to 64 | Included as a matching variable | Considered as a confounder variable | 344 | 3 | 344 | 4 | NR | NR | NR | NR | 0.290 | 1.800 | -1.220 | 0.87 | No |  |
|  | All-cause hospitalisation or death | Hospitalization or death within 30 days of COVID-19 diagnosis | 30 | All-cause composite outcome | >60 | ≥65 | Included as a matching variable | Considered as a confounder variable | 1115 | 44 | 1115 | 41 | NR | NR | NR | NR | 0.270 | 1.790 | -1.250 | 3.68 | No |  |
| Evans 2023 | All-cause admission to hospital or death | Any cause hospitalisation or death (if death occurred without prior admission) within 28 days of a positive COVID-19 test | 28 | All-cause composite outcome | ≥18 | ≥18 | Included as a covariate variable | Considered as a confounder variable | 359 | 14 | 4973 | 544 | HR | 0.49 | 0.29 | 0.83 | NR | NR | NR | NR | NA |  |
| Gmizic 2023 | All-cause hospitalisation | Incidence of hospitalization for any cause which was defined as 24 hours of acute care in a hospital or any similar facility | 25 | NA | ≥18 | NA | Included as a covariate variable | NA | 165 | 40 | 155 | 144 | HR | 0.02 | 0.01 | 0.09 | NR | NR | NR | NR | NA | Not pooled since judged to be at critical risk of bias |
| Kwok 2023 | COVID-19 related hospitalization | Defined by admission to an acute medical ward for management of COVID-19 infection (for more than 24 h) within 14 days of confirmed COVID-19 infection | 14 | NA | ≥18 | NA | Enrolled only unvaccinated patients | NA | 578 | 44 | 2387 | 228 | RR | 0.78 | 0.55 | 1.11 | NR | NR | NR | NR | NA | Not pooled since judged to be at critical risk of bias |
|  | COVID-19 related mortality | Defined as inpatient death during COVID-19 related hospitalization within 14 days of confirmed COVID-19 infection | 14 | NA | ≥18 | NA | Enrolled only unvaccinated patients | NA | NR | 15 | NR | 120 | RR | 0.42 | 0.23 | 0.77 | NR | NR | NR | NR | NA |  |
| Lui 2023 | All-cause mortality or all-cause hospitalisation | All-cause mortality or all-cause hospitalisation at any time during follow-up | 30 | NA | ≥18 | NA | Included as a matching variable | Considered as a confounder variable | 921 | NR | 921 | NR | HR | 0.71 | 0.64 | 0.79 | NR | NR | NR | NR | NA | Not pooled because it is a subgroup analysis of patients with type 2 diabetes mellitus from a larger study (Wong 2023) |
|  | All-cause hospitalisation | All-cause hospitalisation at any time during follow-up | 30 | NA | ≥18 | NA | Included as a matching variable | Considered as a confounder variable | 921 | NR | 921 | NR | HR | 0.71 | 0.64 | 0.79 | NR | NR | NR | NR | NA |  |
|  | All-cause mortality | All-cause mortality at any time during follow-up | 30 | NA | ≥18 | NA | Included as a matching variable | Considered as a confounder variable | 921 | NR | 921 | NR | HR | 0.48 | 0.33 | 0.70 | NR | NR | NR | NR | NA |  |
|  | All-cause mortality or all-cause hospitalisation | All-cause mortality or all-cause hospitalisation at any time during follow-up | 30 | NA | ≥18 | NA | Unvaccinated (0 or 1 dose) group | Unvaccinated | 553 | 365 | 553 | 406 | HR | 0.74 | 0.65 | 0.86 | NR | NR | NR | NR | NA |  |
|  | All-cause mortality or all-cause hospitalisation | All-cause mortality or all-cause hospitalisation at any time during follow-up | 30 | NA | ≥18 | NA | Primary series completed or boosted group | Vaccinated | 361 | 237 | 361 | 297 | HR | 0.69 | 0.58 | 0.81 | NR | NR | NR | NR | NA |  |
| Najjar-Debbiny 2023 | COVID-19-specific mortality | Not defined | 28 | COVID-19 related mortality | ≥18 | ≥18 | Included as a covariate variable | Considered as a confounder variable | 2661 | 22 | 2661 | 27 | HR | 0.81 | 0.46 | 1.43 | NR | NR | NR | NR | NA |  |
|  | COVID-19-specific mortality | Not defined | 28 | COVID-19 related mortality | ≤75 | 18-64 | Included as a covariate variable | Considered as a confounder variable | NR | NR | NR | NR | HR | 2.46 | 1.13 | 5.33 | NR | NR | NR | NR | NA |  |
|  | COVID-19-specific mortality | Not defined | 28 | COVID-19 related mortality | >75 | ≥65 | Included as a covariate variable | Considered as a confounder variable | NR | NR | NR | NR | HR | 0.48 | 0.24 | 0.95 | NR | NR | NR | NR | NA |  |
|  | COVID-19-specific mortality | Not defined | 28 | COVID-19 related mortality | ≥18 | ≥18 | Primary series completed or boostered group | Vaccinated | 2058 | NR | 2058 | NR | HR | 1.78 | 0.79 | 4.03 | NR | NR | NR | NR | NA |  |
|  | COVID-19-specific mortality | Not defined | 28 | COVID-19 related mortality | ≥18 | ≥18 | Unvaccinated (0 or 1 dose) group | Unvaccinated | 603 | NR | 603 | NR | HR | 0.33 | 0.13 | 0.83 | NR | NR | NR | NR | NA |  |
|  | Severe COVID-19 or COVID-19-specific mortality | Severe COVID-19 defined as an oxygen saturation <94% on room air, a ratio of arterial partial pressure of oxygen to fraction of inspired oxygen <300 mm Hg, or a respiratory rate >30 breaths/min. COVID-19 specific mortality was not defined. | 28 | COVID-19 related composite outcome | ≥18 | ≥18 | Included as a covariate variable | Considered as a confounder variable | 2661 | 50 | 2661 | 60 | HR | 0.83 | 0.57 | 1.21 | NR | NR | NR | NR | NA |  |
|  | Severe COVID-19 or COVID-19-specific mortality | Severe COVID-19 defined as an oxygen saturation <94% on room air, a ratio of arterial partial pressure of oxygen to fraction of inspired oxygen <300 mm Hg, or a respiratory rate >30 breaths/min. COVID-19 specific mortality was not defined. | 28 | COVID-19 related composite outcome | ≥18 | ≥18 | Primary series completed or boostered group | Vaccinated | 2058 | NR | 2058 | NR | HR | 1.36 | 0.81 | 2.28 | NR | NR | NR | NR | NA |  |
|  | Severe COVID-19 or COVID-19-specific mortality | Severe COVID-19 defined as an oxygen saturation <94% on room air, a ratio of arterial partial pressure of oxygen to fraction of inspired oxygen <300 mm Hg, or a respiratory rate >30 breaths/min. COVID-19 specific mortality was not defined. | 28 | COVID-19 related composite outcome | ≥18 | ≥18 | Unvaccinated (0 or 1 dose) group | Unvaccinated | 603 | NR | 603 | NR | HR | 0.45 | 0.25 | 0.82 | NR | NR | NR | NR | NA |  |
|  | Severe COVID-19 or COVID-19-specific mortality | Severe COVID-19 defined as an oxygen saturation <94% on room air, a ratio of arterial partial pressure of oxygen to fraction of inspired oxygen <300 mm Hg, or a respiratory rate >30 breaths/min. COVID-19 specific mortality was not defined. | 28 | COVID-19 related composite outcome | ≤75 | 18-64 | Included as a covariate variable | Considered as a confounder variable | NR | NR | NR | NR | HR | 2.46 | 1.13 | 5.33 | NR | NR | NR | NR | NA |  |
|  | Severe COVID-19 or COVID-19-specific mortality | Severe COVID-19 defined as an oxygen saturation <94% on room air, a ratio of arterial partial pressure of oxygen to fraction of inspired oxygen <300 mm Hg, or a respiratory rate >30 breaths/min. COVID-19 specific mortality was not defined. | 28 | COVID-19 related composite outcome | >75 | ≥65 | Included as a covariate variable | Considered as a confounder variable | NR | NR | NR | NR | HR | 0.54 | 0.34 | 0.86 | NR | NR | NR | NR | NA |  |
| Paraskevis 2023 | Hospitalization for COVID-19 | Hospitalization for COVID-19 within 10 days after a positive SARS-CoV-2 test result with no ICU admission or clinical deterioration (intubation) | 10 | NA | ≥65 | NA | Included as a covariate variable | Considered as a confounder variable | 4240 | 149 | 4240 | 357 | OR | 0.40 | 0.32 | 0.48 | NR | NR | NR | NR | NA | Not pooled since judged to be at critical risk of bias |
| Tazare 2023 | COVID-19-related hospitalisation or COVID-19-related death | COVID-19-related hospitalisation (based on primary diagnosis ascertained from SUS) or COVID-19-related death (based on underlying/contributing causes) within 28-days of SARS-CoV-2 infection | 28 | COVID-19 related composite outcome | ≥18 | ≥18 | Included as a covariate variable | Considered as a confounder variable | 3072 | NR | 65574 | NR | HR | 1.09 | 0.93 | 1.27 | -0.700 | -1.700 | 0.200 | 3.25 | No | Estimates were provided for two different time periods, which were dominated by Omicron BA.1 and Omicron BA.2. Given the evolution of Omicron subvariants, we extracted data for the comparison of molnupiravir in the later time period of the study, February 11 to May 21, 2023, when Omicron BA.2 was the dominant circulating variant in England. |
| Van Heer 2023 | Hospitalisation due to any cause | Defined through a VICNISS flag in the case database, TREVI, which involved hospital clinicians reporting all COVID-19 cases admitted to hospital during their infectious period, defined as 7 days following an initial positive COVID-19 PCR or RAT, or assessed as infectious (‘activeCOVID-19’), regardless of the reason for admission | 35 | All-cause hospitalisation | ≥70 | ≥65 | Included as a covariate variable | Considered as a confounder variable | 15673 | 195 | 10637 | 185 | OR | 0.71 | 0.58 | 0.87 | NR | NR | NR | NR | NA |  |
|  | COVID-19 associated mortality | Defined as per the Victorian DH surveillance definition—COVID-19 listed as a primary or contributing cause of death on the medical death certificate, or a death within 35 days of diagnosis, excluding trauma/accidents and suicide | 35 | COVID-19 related mortality | ≥70 | ≥65 | Included as a covariate variable | Considered as a confounder variable | 19962 | 346 | 13721 | 462 | OR | 0.45 | 0.38 | 0.54 | NR | NR | NR | NR | NA |  |
| Wong 2022 | All-cause mortality | Defined as patients who died within 28 days of confirmed SARS-CoV-2 infection | 28 | All-cause mortality | ≥18 | ≥18 | Included as a matching variable | Considered as a confounder variable | 4983 | NR | 49234 | NR | HR | 0·76 | 0.61 | 0.95 | NR | NR | NR | NR | NA |  |
|  | Hospital admission due to COVID-19 | Defined as patients admitted to hospital as a result of COVID-19 within 28 days of confirmed SARS-CoV-2 infection (no diagnosis codes were described) | 28 | COVID-19 related hospitalisation | ≥18 | ≥18 | Included as a matching variable | Considered as a confounder variable | 4983 | NR | 49234 | NR | HR | 0·98 | 0.89 | 1.05 | NR | NR | NR | NR | NA |  |
|  | In-hospital disease progression | Defined as composite of in-hospital mortality, invasive mechanical ventilation, or intensive care unit admission | 28 | All-cause composite outcome | ≥18 | ≥18 | Included as a matching variable | Considered as a confounder variable | 4983 | NR | 49234 | NR | HR | 0·57 | 0.43 | 0.76 | NR | NR | NR | NR | NA |  |
|  | All-cause mortality | Defined as patients who died within 28 days of confirmed SARS-CoV-2 infection | 28 | All-cause mortality | ≥18 | ≥18 | Primary series completed or boostered group | Vaccinated | 800 | NR | 6115 | NR | HR | 0.66 | 0.16 | 2.77 | NR | NR | NR | NR | NA |  |
|  | All-cause mortality | Defined as patients who died within 28 days of confirmed SARS-CoV-2 infection | 28 | All-cause mortality | ≥18 | ≥18 | Unvaccinated (0 or 1 dose) group | Unvaccinated | 4183 | NR | 43119 | NR | HR | 0.85 | 0.70 | 1.05 | NR | NR | NR | NR | NA |  |
|  | All-cause mortality | Defined as patients who died within 28 days of confirmed SARS-CoV-2 infection | 28 | All-cause mortality | ≤60 | 18-64 | Included as a matching variable | Considered as a confounder variable | 565 | NR | 3819 | NR | HR | 2.31 | 0.77 | 6.90 | NR | NR | NR | NR | NA |  |
|  | All-cause mortality | Defined as patients who died within 28 days of confirmed SARS-CoV-2 infection | 28 | All-cause mortality | >60 | ≥65 | Included as a matching variable | Considered as a confounder variable | 4418 | NR | 45415 | NR | HR | 0.75 | 0.60 | 0.93 | NR | NR | NR | NR | NA |  |
|  | Hospital admission due to COVID-19 | Defined as patients admitted to hospital as a result of COVID-19 within 28 days of confirmed SARS-CoV-2 infection (no diagnosis codes were described) | 28 | COVID-19 related hospitalisation | ≥18 | ≥18 | Primary series completed or boostered group | Vaccinated | 800 | NR | 6115 | NR | HR | 0.66 | 0.42 | 1.02 | NR | NR | NR | NR | NA |  |
|  | Hospital admission due to COVID-19 | Defined as patients admitted to hospital as a result of COVID-19 within 28 days of confirmed SARS-CoV-2 infection (no diagnosis codes were described) | 28 | COVID-19 related hospitalisation | ≥18 | ≥18 | Unvaccinated/partially vaccinated | Unvaccinated | 4183 | NR | 43119 | NR | HR | 1.01 | 0.93 | 1.10 | NR | NR | NR | NR | NA |  |
|  | Hospital admission due to COVID-19 | Defined as patients admitted to hospital as a result of COVID-19 within 28 days of confirmed SARS-CoV-2 infection (no diagnosis codes were described) | 28 | COVID-19 related hospitalisation | ≤60 | 18-64 | Included as a matching variable | Considered as a confounder variable | 565 | NR | 3819 | NR | HR | 1.15 | 0.84 | 1.59 | NR | NR | NR | NR | NA |  |
|  | Hospital admission due to COVID-19 | Defined as patients admitted to hospital as a result of COVID-19 within 28 days of confirmed SARS-CoV-2 infection (no diagnosis codes were described) | 28 | COVID-19 related hospitalisation | >60 | ≥65 | Included as a matching variable | Considered as a confounder variable | 4418 | NR | 45415 | NR | HR | 0.89 | 0.81 | 0.97 | NR | NR | NR | NR | NA |  |
|  | In-hospital disease progression | Defined as composite of in-hospital mortality, invasive mechanical ventilation, or intensive care unit admission | 28 | All-cause composite outcome | ≥18 | ≥18 | Primary series completed or received boosters | Vaccinated | 800 | NR | 6115 | NR | HR | 0.58 | 0.14 | 2.43 | NR | NR | NR | NR | NA |  |
|  | In-hospital disease progression | Defined as composite of in-hospital mortality, invasive mechanical ventilation, or intensive care unit admission | 28 | All-cause composite outcome | ≥18 | ≥18 | Unvaccinated (0 or 1 dose) group | Unvaccinated | 4183 | NR | 43119 | NR | HR | 0.67 | 0.51 | 0.87 | NR | NR | NR | NR | NA |  |
|  | In-hospital disease progression | Defined as composite of in-hospital mortality, invasive mechanical ventilation, or intensive care unit admission | 28 | All-cause composite outcome | ≤60 | 18-64 | Included as a matching variable | Considered as a confounder variable | 565 | NR | 3819 | NR | HR | 1.18 | 0.35 | 3.98 | NR | NR | NR | NR | NA |  |
|  | In-hospital disease progression | Defined as composite of in-hospital mortality, invasive mechanical ventilation, or intensive care unit admission | 28 | All-cause composite outcome | >60 | ≥65 | Included as a matching variable | Considered as a confounder variable | 4418 | NR | 45415 | NR | HR | 0.55 | 0.42 | 0.73 | NR | NR | NR | NR | NA |  |
| Xie 2023 | All-cause hospital admission or death | Hospital admission based on information from the inpatient database and death based on patient vital status data | 30 | All-cause composite outcome | ≥18 | ≥18 | Included as a covariate variable | Considered as a confounder variable | 7818 | 262 | 78180 | 2850 | RR | 0.72 | 0.64 | 0.79 | 1.100 | 0.800 | 1.400 | 3.80 | No |  |
|  | Hospital admission | Hospital admission based on information from the inpatient database | 30 | All-cause hospitalisation | ≥18 | ≥18 | Included as a covariate variable | Considered as a confounder variable | 7818 | 252 | 78180 | 2325 | RR | 0.80 | 0.71 | 0.90 | 0.600 | 0.300 | 0.900 | 3.10 | No |  |
|  | Death | Death based on patient vital status data | 30 | All-cause mortality | ≥18 | ≥18 | Included as a covariate variable | Considered as a confounder variable | 7818 | 11 | 78180 | 569 | RR | 0.35 | 0.24 | 0.49 | 0.500 | 0.400 | 0.600 | 0.80 | No |  |
|  | All-cause hospital admission or death | Hospital admission based on information from the inpatient database and death based on patient vital status data | 30 | All-cause composite outcome | ≥18 | ≥18 | Unvaccinated (0 or 1 dose) group | Unvaccinated | 1107 | 362 | 13698 | 2850 | RR | 0.83 | 0.70 | 0.97 | 0.900 | 0.200 | 1.900 | 5.70 | No |  |
|  | All-cause hospital admission or death | Hospital admission based on information from the inpatient database and death based on patient vital status data | 30 | All-cause composite outcome | ≥18 | ≥18 | Received first booster or more group | Vaccinated | 4631 | NR | 20287 | NR | RR | 0.71 | 0.58 | 0.83 | 1.000 | 0.500 | 1.400 | 3.30 | No |  |
|  | All-cause hospital admission or death | Hospital admission based on information from the inpatient database and death based on patient vital status data | 30 | All-cause composite outcome | ≤65 | 18 to 64 | Included as a covariate variable | Considered as a confounder variable | NR | NR | NR | NR | RR | 0.74 | 0.57 | 0.93 | 0.900 | 0.200 | 1.500 | 3.30 | No |  |
|  | All-cause hospital admission or death | Hospital admission based on information from the inpatient database and death based on patient vital status data | 30 | All-cause composite outcome | >65 | ≥65 | Included as a covariate variable | Considered as a confounder variable | NR | NR | NR | NR | RR | 0.75 | 0.51 | 0.85 | 1.100 | 0.700 | 1.600 | 4.40 | No |  |
| Abbreviations: COVID-19: coronavirus disease 2019; HR, hazard ratio; ICU, intensive care unit; NA, not applicable; NR, not reported; OR, odds ratio; RD, risk difference; RR, risk ratio. Negative RD estimates indicate a higher risk of the outcome with the oral antiviral drug (intervention) | | | | | | | | | | | | | | | | | | | | | | |

**Table S3.** List of studies and reasons for exclusion after full text review

| **Wrong study design** | | | |
| --- | --- | --- | --- |
| **#** | **Citation** | | **Rationale** |
| 1 | Belden KA, Yeager S, Schulte J, et al. "Saving lives with nirmatrelvir/ritonavir one transplant patient at a time". *Transpl Infect Dis* 2023;25(2):e14037. doi: 10.1111/tid.14037 | | Single arm study examining changes in tacrolimus concentration |
| 2 | Bernal AJ, Gomes da Silva MM, Musungaie DB, et al. Molnupiravir for oral treatment of COVID-19 in nonhospitalized patients. *N Engl J Med* 2022;386(6):509-20. doi: 10.1056/NEJMoa2116044 | | Randomized controlled trial |
| 3 | Bolkun L, Pula B, Kolkowska-Lesniak A, et al. Molnupiravir is effective in patients with haematological malignancies. *Int J Cancer* 2023;153(6):1251-56. doi: 10.1002/ijc.34442 | | Descriptive study |
| 4 | Cheng SL, Wang PH, Chang CY, et al. The benefits of molnupiravir treatment in healthcare facilities patients with COVID-19. *Drug Des Devel Ther* 2023;17:87-92. doi: 10.2147/DDDT.S392708 | | Single arm study of molnupiravir |
| 5 | Chew LS, Lim XJ, Chang CT, et al. Effectiveness of nirmatrelvir/ritonavir (Paxlovid®) in preventing hospitalisation and death among COVID-19 patients: a prospective cohort study. *Med J Malaysia* 2023;78(5):602-08. | | Descriptive study of nirmatrelvir/ritonavir compared to historical controls |
| 6 | Czarnecka K, Czarnecka P, Tronina O, et al. Molnupiravir outpatient treatment for adults with COVID-19 in a real-world setting-a single center experience. *J Clin Med* 2022;11(21) doi: 10.3390/jcm11216464 | | Descriptive study |
| 7 | Del Borgo C, Garattini S, Bortignon C, et al. Effectiveness, tolerability and prescribing choice of antiviral molecules molnupiravir, remdesivir and nirmatrelvir/r: a real-world comparison in the first ten months of use. *Viruses* 2023;15(4) doi: 10.3390/v15041025 | | No control group, only includes antiviral interventions |
| 8 | Drysdale M, Tibble H, Patel V, et al. Characteristics and outcomes of patients with COVID-19 at high risk of disease progression receiving sotrovimab, oral antivirals or no treatment in Scotland (preprint). *medRxiv* 2023:2023.06.09.23291195. doi: 10.1101/2023.06.09.23291195 | | Descriptive study |
| 9 | Ebell MH. Nirmatrelvir/Ritonavir reduces risk of hospitalization in at-risk outpatients. *Am Fam Physician* 2022;105(5):Online. | | Review of a clinical trial |
| 10 | Ebell MH. Nirmatrelvir/ritonavir reduces hospitalization, mortality in patients 65 years and older with COVID-19; effect on younger patients unclear. *Am Fam Physician* 2023;107(3):316. | | Review of a clinical trial |
| 11 | Hammond J, Leister-Tebbe H, Gardner A, et al. Oral nirmatrelvir for high-risk, nonhospitalized adults with COVID-19. *N Engl J Med* 2022;386(15):1397-408. doi: 10.1056/NEJMoa2118542 | | Randomized controlled trial |
| 12 | Imai M, Ito M, Kiso M, et al. Efficacy of antiviral agents against Omicron subvariants BQ.1.1 and XBB. *N Engl J Med* 2023;388(1):89-91. doi: 10.1056/NEJMc2214302 | | In vitro study |
| 13 | Kane AM, Keenan EM, Lee K, et al. Nirmatrelvir-ritonavir treatment of COVID-19 in a high-risk patient population: A retrospective observational study. *J Am Coll Clin Pharm* 2023;6(1):29-33. doi: 10.1002/jac5.1729 | | Single arm study of nirmatrelvir/ritonavir |
| 14 | Kauer V, Totschnig D, Waldenberger F, et al. Efficacy of sotrovimab (SOT), molnupiravir (MOL), and nirmatrelvir/ritponavir (N/R) and tolerability of molnupiravir in outpatients at high risk for severe COVID-19. *Viruses* 2023;15(5) doi: 10.3390/v15051181 | | Descriptive study |
| 15 | Kimata M, Watanabe A, Yanagida Y, et al. Safety and effectiveness of molnupiravir (LAGEVRIO((R))) capsules in Japanese patients with COVID-19: interim report of post-marketing surveillance in Japan. *Infect Dis Ther* 2023;12(4):1119-36. doi: 10.1007/s40121-023-00782-5 | | Single arm registry study and survey |
| 16 | Lin CY, Cassidy AG, Li L, et al. Nirmatrelvir-ritonavir (Paxlovid) for mild coronavirus disease 2019 (COVID-19) in pregnancy and lactation. *Obstet Gynecol* 2023;141(5):957-60. doi: 10.1097/AOG.0000000000005152 | | Cross-sectional study |
| 17 | Liu J, Pan X, Zhang S, et al. Efficacy and safety of Paxlovid in severe adult patients with SARS-Cov-2 infection: a multicenter randomized controlled study. *Lancet Reg Health West Pac* 2023;33:100694. doi: 10.1016/j.lanwpc.2023.100694 | | Randomized controlled trial |
| 18 | Martin-Blondel G, Marcelin AG, Soulie C, et al. Time to negative PCR conversion amongst high-risk patients with mild-to-moderate Omicron BA.1 and BA.2 COVID-19 treated with sotrovimab or nirmatrelvir. *Clin Microbiol Infect* 2023;29(4):543.e5-43.e9. doi: 10.1016/j.cmi.2022.12.016 | | Single arm study of molnupiravir or sotrovimab |
| 19 | Mutoh Y, Umemura T, Nishikawa T, et al. Real-world experience of the comparative effectiveness and safety of molnupiravir and nirmatrelvir/ritonavir in high-risk patients with COVID-19 in a community setting. *Viruses* 2023;15(3) doi: 10.3390/v15030811 | | Descriptive study |
| 20 | Orbak Z, Laloglu F, Akat H. Effectiveness of lopinavir/ritonavir on COVID-19-related pneumonia in a child with COVID-19-associated Kawasaki disease. *Cardiol Young* 2021;31(3):507-10. doi: 10.1017/S1047951120004291 | | Case report |
| 21 | Poznanski P, Augustyniak-Bartosik H, Magiera-Zak A, et al. Molnupiravir when used alone seems to be safe and effective as outpatient COVID-19 therapy for hemodialyzed patients and kidney transplant recipients. *Viruses* 2022;14(10) doi: 10.3390/v14102224 | | Single arm study of molnupiravir |
| 22 | Prajapati G, Das A, Sun Y, et al. Hospitalization among patients treated with molnupiravir: a retrospective study of administrative data. *Clin Ther* 2023;45(10):957-64. doi: 10.1016/j.clinthera.2023.07.018 | | Descriptive study |
| 23 | Razonable RR, O’Horo JC, Hanson SN, et al. Comparable outcomes for bebtelovimab and ritonavir-boosted nirmatrelvir treatment in high-risk patients with coronavirus disease-2019 during Severe Acute Respiratory Syndrome Coronavirus 2 BA.2 Omicron epoch. *J Infect Dis* 2022;226(10):1683-87. doi: 10.1093/infdis/jiac346 | | Single arm study of nirmatrelvir/ritonavir |
| 24 | Sacks HS. In nonhospitalized, unvaccinated adults with COVID-19, molnupiravir reduced hospitalization or death at 29 d. *Ann Intern Med* 2022;175(4):JC40. doi: 10.7326/J22-0017 | | Review of a clinical trial |
| 25 | Sinha S, N K, Suram VK, et al. Efficacy and safety of molnupiravir in mild COVID-19 patients in India. *Cureus* 2022;14(11):e31508. doi: 10.7759/cureus.31508 | | Randomized controlled trial |
| 26 | Streinu-Cercel A, Miron VD, Oana AA, et al. Real-world use of molnupiravir in the treatment of outpatients with SARS-CoV-2 infection-a patient profile based on the experience of a tertiary infectious disease center. *Pharmaceuticals (Basel)* 2022;15(9) doi: 10.3390/ph15091065 | | Single arm study of molnupiravir |
| 27 | Tiseo G, Barbieri C, Galfo V, et al. Efficacy and safety of nirmatrelvir/ritonavir, molnupiravir, and remdesivir in a real-world cohort of outpatients with COVID-19 at high risk of progression: the PISA outpatient clinic experience. *Infect Dis Ther* 2023;12(1):257-71. doi: 10.1007/s40121-022-00729-2 | | Cohort study of molnupiravir and nirmatrelvir/ritonavir without any untreated comparator group |
| 28 | Vassilopoulos A, Mylonakis E. In patients with COVID-19 at risk for severe disease, nirmatrelvir + ritonavir reduced hospitalization or death. *Ann Intern Med* 2022;175(6):JC63. doi: 10.7326/J22-0038 | | Review of a clinical trial |
| 29 | Vena A, Traman L, Bavastro M, et al. Early clinical experience with molnupiravir for mild to moderate breakthrough COVID-19 among fully vaccinated patients at risk for disease progression. *Vaccines (Basel)* 2022;10(7) doi: 10.3390/vaccines10071141 | | Single arm study of molnupiravir |
| **Wrong outcomes** | | | |
| **#** | **Citation** | | **Rationale** |
| 1 | Chuang MH, Wu JY, Liu TH, et al. Efficacy of nirmatrelvir and ritonavir for post-acute COVID-19 sequelae beyond 3 months of SARS-CoV-2 infection. *J Med Virol* 2023;95(4):e28750. doi: 10.1002/jmv.28750 | | Outcome was post-COVID-19 condition |
| 2 | Durstenfeld MS, Peluso MJ, Lin F, et al. Association of nirmatrelvir/ritonavir treatment with long COVID symptoms in an online cohort of non-hospitalized individuals experiencing breakthrough SARS-CoV-2 infection in the Omicron era (preprint). *medRxiv* 2023:2023.03.02.23286730. doi: 10.1101/2023.03.02.23286730 | | Outcome was long COVID-19 symptoms |
| 3 | Hafez W, Saleh H, Al Baha Z, et al. Antiviral used among non-severe COVID-19 cases in relation to time till viral clearance: a retrospective cohort study. *Antibiotics (Basel)* 2022;11(4) doi: 10.3390/antibiotics11040498 | | Outcome was time to viral clearance |
| 4 | Lim S, Tignanelli CJ, Hoertel N, et al. Prevalence of medical contraindications to nirmatrelvir/ritonavir in a cohort of hospitalized and nonhospitalized patients with COVID-19. *Open Forum Infect Dis* 2022;9(8):ofac389. doi: 10.1093/ofid/ofac389 | | Outcome is prevalence of contraindication to nirmatrelvir/ritonavir |
| 5 | Patel R, Dani S, Khadke S, et al. Incidence of symptoms associated with post-acute sequelae of SARS-CoV-2 infection in non-hospitalized vaccinated patients receiving nirmatrelvir-ritonavir (preprint). *medRxiv* 2023:2023.04.05.23288196. doi: 10.1101/2023.04.05.23288196 | | Outcome is presence of symptoms associated with post-acute sequelae of SARS-CoV-2 infection |
| 6 | Wang Y, Zhao D, Chen X, et al. The effect of nirmatrelvir-ritonavir on viral clearance and length of hospital stay in patients infected with SARS-CoV-2 Omicron variants. *Influenza Other Respi Viruses* 2023;17(2):e13095. doi: 10.1111/irv.13095 | | Outcome is viral clearance and length of hospital stay |
| 7 | Weng C, Xie R, Han G, et al. Safety and efficacy of Paxlovid against Omicron variants of coronavirus disease 2019 in elderly patients. *Infect Dis Ther* 2023;12(2):649-62. doi: 10.1007/s40121-023-00760-x | | Outcome is viral clearance and length of hospital stay |
| 8 | Wong CKH, Lau KTK, Au ICH, et al. Viral burden rebound in hospitalised patients with COVID-19 receiving oral antivirals in Hong Kong: a population-wide retrospective cohort study. *Lancet Infect Dis* 2023;23(6):683-95. doi: 10.1016/S1473-3099(22)00873-8 | | Outcome is viral burden rebound |
| 9 | Wong GLH, Yip TCF, Lai MSM, et al. Incidence of viral rebound after treatment with nirmatrelvir-ritonavir and molnupiravir. *JAMA Netw Open* 2022;5(12):e2245086. doi: 10.1001/jamanetworkopen.2022.45086 | | Outcome is viral burden rebound |
| **Wrong population** | | | |
| **#** | **Citation** | | **Rationale** |
| 1 | Deng G, Li D, Sun Y, et al. Real-world effectiveness of azvudine versus nirmatrelvir-ritonavir in hospitalized patients with COVID-19: a retrospective cohort study. *J Med Virol* 2023;95(4):e28756. doi: 10.1002/jmv.28756 | | Hospitalised patients |
| 2 | Kim JM, Yoo MG, Bae SJ, et al. Effectiveness of Paxlovid, an oral antiviral drug, against the Omicron BA.5 variant in Korea: severe progression and death between July and November 2022. *J Korean Med Sci* 2023;38(27):e211. doi: 10.3346/jkms.2023.38.e211 | | Unclear if patients are hospitalised and non-hospitalised |
| 3 | Kim M-K, Lee K-S, Ham SY, et al. Real-world effectiveness of nirmatrelvir-ritonavir and its acceptability in high-risk COVID-19 patients. *J Korean Med Sci* 2023;38(35):e272. doi: 10.3346/jkms.2023.38.e272 | | Hospitalised patients |
| 4 | Lee E, Park S, Choi JP, et al. Short-term effectiveness of oral nirmatrelvir/ritonavir against the SARS-CoV-2 Omicron variant and culture-positive viral shedding. *J Korean Med Sci* 2023;38(8):e59. doi: 10.3346/jkms.2023.38.e59 | | Hospitalised patients |
| 5 | Li H, Gao M, You H, et al. Association of nirmatrelvir/ritonavir treatment on upper respiratory Severe Acute Respiratory Syndrome Coronavirus 2 Reverse Transcription-Polymerase Chain Reaction (SARS-Cov-2 RT-PCR) negative conversion rates among high-risk patients with coronavirus disease 2019 (COVID-19). *Clin Infect Dis* 2023;76(3):e148-e54. doi: 10.1093/cid/ciac600 | | Hospitalised patients |
| 6 | Mitsushima S, Horiguchi H, Taniguchi K. Effectiveness of drugs for COVID-19 inpatients in Japanese medical claim data as average treatment effects with inverse probability weighted regression adjustment: retrospective observational study (preprint). *medRxiv* 2023:2023.05.12.23289913. doi: 10.1101/2023.05.12.23289913 | | Hospitalised patients |
| 7 | Park H, Park YJ, Lee HY, et al. The effectiveness of Paxlovid treatment in long-term care facilities in South Korea during the outbreak of the Omicron variant of SARS-CoV-2. *Osong Public Health Res Perspect* 2022;13(6):443-47. doi: 10.24171/j.phrp.2022.0262 | | Long-term care patients and workers |
| 8 | Qi T, Jin Y, Wang H, et al. Nirmatrelvir-ritonavir therapy and COVID-19 vaccination improve clinical outcomes of SARS-CoV-2 Omicron variant infection. *J Med Virol* 2023;95(2):e28497. doi: 10.1002/jmv.28497 | | Hospitalised patients |
| 9 | Suzuki Y, Shibata Y, Minemura H, et al. Real-world clinical outcomes of treatment with molnupiravir for patients with mild-to-moderate coronavirus disease 2019 during the Omicron variant pandemic. *Clin Exp Med* 2022:1-9. doi: 10.1007/s10238-022-00949-3 | | Hospitalised patients |
| 10 | Wan EYF, Yan VKC, Mok AHY, et al. Effectiveness of molnupiravir and nirmatrelvir–ritonavir in hospitalized patients with COVID-19: a target trial emulation study. *Ann Intern Med* 2023;176(4):505-14. doi: 10.7326/M22-3057 | | Hospitalised patients |
| 11 | Wong CKH, Au ICH, Lau KTK, et al. Real-world effectiveness of early molnupiravir or nirmatrelvir-ritonavir in hospitalised patients with COVID-19 without supplemental oxygen requirement on admission during Hong Kong's omicron BA.2 wave: a retrospective cohort study. *Lancet Infect Dis* 2022;22(12):1681-93. doi: 10.1016/S1473-3099(22)00507-2 | | Hospitalised patients |
| 12 | Yan J, Cai H, Wang J, et al. Nirmatrelvir/ritonavir for patients with SARS-CoV-2 infection and impaired kidney function during the Omicron surge. *Front Pharmacol* 2023;14:1147980. doi: 10.3389/fphar.2023.1147980 | | Hospitalised patients |
| 13 | Yii Y-C, Shih H-M, Chen C-L, et al. Impact of pre-hospitalization use of oral antiviral agents on reducing critical illness and mortality for patients with COVID-19 pneumonia. *Int J Antimicrob Agents* 2023:107020. doi: 10.1016/j.ijantimicag.2023.107020 | | Hospitalised patients |
| **Vaccination status not available or not reported** | | | |
| **#** | **Citation** | | **Rationale** |
| 1 | Arbel R, Wolff Sagy Y, Hoshen M, et al. Nirmatrelvir use and severe COVID-19 outcomes during the Omicron surge. *N Engl J Med* 2022;387(9):790-98. doi: 10.1056/NEJMoa2204919 | | Report only SARS-CoV-2 immunity status which integrates prior infection and vaccination |
| 2 | Arbel R, Sagy YW, Battat E, et al. Molnupiravir use and severe COVID-19 outcomes during the Omicron surge (preprint version 1). *Research Square* 2022 29 Sept 2022. https://www.researchsquare.com/article/rs-2115769/v1. | | Report only SARS-CoV-2 immunity status which integrates prior infection and vaccination |
| 3 | Flisiak R, Zarebska-Michaluk D, Rogalska M, et al. Real-world experience with molnupiravir during the period of SARS-CoV-2 Omicron variant dominance. *Pharmacol Rep* 2022;74(6):1279-85. doi: 10.1007/s43440-022-00408-6 | | Vaccination status not available |
| 4 | Hansen K, Makkar SR, Sahner D, et al. Paxlovid (nirmatrelvir/ritonavir) effectiveness against hospitalization and death in N3C: A target trial emulation study (preprint). medRxiv 2023 doi: 10.1101/2023.05.26.23290602 | | Unclear if vaccination status was available and included in regression models for all patients included in study |
| 5 | Ma BH, Yip TC, Lui GC, et al. Clinical outcomes following treatment for COVID-19 with nirmatrelvir/ritonavir and molnupiravir among patients living in nursing homes. *JAMA Netw Open* 2023;6(4):e2310887. doi: 10.1001/jamanetworkopen.2023.10887 | | Individual-level vaccination status not available |
| 6 | Tadmor T, Alapi H, Rokach L. Effectiveness of nirmatrelvir plus ritonavir treatment for patients with chronic lymphocytic leukemia during the Omicron surge. *Blood* 2023;141(18):2239-44. doi: 10.1182/blood.2022019017 | | Vaccination status not reported |
| 7 | Wai AKC, Chan CY, Cheung AWL, et al. Association of molnupiravir and nirmatrelvir-ritonavir with preventable mortality, hospital admissions and related avoidable healthcare system cost among high-risk patients with mild to moderate COVID-19: benefits from COVID-19 antiviral drugs. *Lancet Reg Health West Pac* 2023;30:100602. doi: 10.1016/j.lanwpc.2022.100602 | | Vaccination status not available |
| 8 | Yip TC, Lui GC, Lai MS, et al. Impact of the use of oral antiviral agents on the risk of hospitalization in community coronavirus disease 2019 Patients (COVID-19). *Clin Infect Dis* 2023;76(3):e26-e33. doi: 10.1093/cid/ciac687 | | Individual-level vaccination status not available |
| **Wrong outcome follow-up time** | | | |
| **#** | **Citation** | | **Rationale** |
| 1 | Hsu WH, Tsai YW, Wu JY, et al. Post-acute hospitalization and mortality of nirmatrelvir plus ritonavir for COVID-19 survivors. *J Infect* 2023;86(4):e107-e10. doi: 10.1016/j.jinf.2023.02.007 | | Primary outcome assessed between one and six months after the diagnosis of COVID-19 |
| 2 | Lin D-Y, Abi Fadel F, Huang S, et al. Nirmatrelvir or molnupiravir use and severe outcomes from Omicron infections. *JAMA Netw Open* 2023;6(9):e2335077. doi: 10.1001/jamanetworkopen.2023.35077 | | Primary outcome assessed up to 90 days after the diagnosis of COVID-19 |
| **Wrong intervention** | | | |
| **#** | **Citation** | | **Rationale** |
| 1 | Gentry CA, Nguyen P, Thind SK, et al. Characteristics and outcomes of US Veterans at least 65 years of age at high risk of severe SARS-CoV-2 infection with or without receipt of oral antiviral agents. *J Infect* 2023;86(3):248-55. doi: 10.1016/j.jinf.2023.01.018 | | Nirmatrelvir/ritonavir and molnupiravir were group into a single intervention group “patients who received an oral antiviral” |
| 2 | Gentry CA, Nguyen PN, Thind SK, et al. Characteristics and outcomes of US Veterans with immunocompromised conditions at high risk of severe SARS-CoV-2 infection with or without receipt of oral antiviral agents. *Clin Infect Dis* 2023 doi: 10.1093/cid/ciad504 | | Nirmatrelvir/ritonavir and molnupiravir were group into a single intervention group “patients who received an oral antiviral” |
| 3 | Jain J, Chandak D, Basak S. Clinical outcomes in COVID-19 patients treated with antivirals: a retrospective analysis. *J Assoc Physicians India* 2022;70(4):11-12. | | Did not assess nirmatrelvir/ritonavir or molnupiravir |
| **Wrong comparator** | | | |
| **#** | **Citation** | | **Rationale** |
| 1 | Gentile I, Scotto R, Schiano Moriello N, et al. Nirmatrelvir/ritonavir and molnupiravir in the treatment of mild/moderate COVID-19: results of a real-life study. *Vaccines (Basel)* 2022;10(10) doi: 10.3390/vaccines10101731 | | Compared nirmatrelvir/ritonavir and molnupiravir |
| 2 | Rinaldi M, Campoli C, Gallo M, et al. Comparison between available early antiviral treatments in outpatients with SARS-CoV-2 infection: a real-life study. *BMC Infect Dis* 2023;23(1):646. doi: 10.1186/s12879-023-08538-9 | | Compared nirmatrelvir/ritonavir, molnupiravir and remdesivir |
| 3 | Torti C, Olimpieri PP, Bonfanti P, et al. Real-life comparison of mortality in patients with SARS-CoV-2 infection at risk for clinical progression treated with molnupiravir or nirmatrelvir plus ritonavir during the Omicron era in Italy: a nationwide, cohort study. *Lancet Reg Health Eur* 2023;31:100684. doi: 10.1016/j.lanepe.2023.100684 | | Compared nirmatrelvir/ritonavir and molnupiravir |
| 4 | Zheng B, Tazare J, Nab L, et al. Comparative effectiveness of nirmatrelvir/ritonavir versus sotrovimab and molnupiravir for preventing severe COVID-19 outcomes in non-hospitalised high-risk patients during Omicron waves: observational cohort study using the OpenSAFELY platform. *Lancet Reg Health Eur* 2023;34:100741. doi: 10.1016/j.lanepe.2023.100741 | | Compared nirmatrelvir/ritonavir to sotrovimab and molnupiravir |
| 5 | Zheng B, Green ACA, Tazare J, et al. Comparative effectiveness of sotrovimab and molnupiravir for prevention of severe covid-19 outcomes in patients in the community: observational cohort study with the OpenSAFELY platform. *BMJ* 2022;379:e071932. doi: 10.1136/bmj-2022-071932 | | Compared sotrovimab and molnupiravir |
| **Intervention initiation window not defined** | | | |
| **#** | **Citation** | | **Rationale** |
| 1 | Al-Obaidi MM, Gungor AB, Murugapandian S, et al. The impact of nirmatrelvir-ritonavir in reducing hospitalizations among high-risk patients with SARS-CoV-2 during the Omicron predominant era. *Am J Med* 2023;136(6):577-84. doi: 10.1016/j.amjmed.2023.02.022 | | Did not define number of days after positive test for SARS-CoV-2 to nirmatrelvir/ritonavir initiation |
| 2 | Kaboré JL, Laffont B, Diop M, et al. Real-world effectiveness of nirmatrelvir/ritonavir on Covid-19-associated hospitalization prevention: a population-based cohort study in the province of Quebec, Canada. *Clin Infect Dis* 2023(a4j, 9203213) doi: 10.1093/cid/ciad287 | | Did not define number of days after positive test for SARS-CoV-2 to nirmatrelvir/ritonavir initiation |
| **Limited reporting of methods or ineligible publication type** | | | |
| **#** | **Citation** | **Rationale** | |
| 1 | Henderson H, Wohl DA, Fischer W, et al. COVID-19 hospitalization risk after SARS-CoV-2 vaccination and outpatient treatment. *Top Antivir Med* 2023;31(2):71-71. | Conference abstract | |
| 2 | Liu TH, Hsu WH, Tsai YW, et al. Clinical effectiveness of nirmatrelvir plus ritonavir in the treatment of COVID-19 for patients with dementia. *J Am Med Dir Assoc* 2023;24(8):1159-62. doi: 10.1016/j.jamda.2023.05.034 | Research letter with limited description of study methods | |

**Table S4**. Characteristics of the observational studies included in the systematic review of oral antiviral drug effectiveness for outpatients with COVID-19

| **Study overview and setting** | | | | | | | | | | **Interventions and comparators** | | | **Baseline characteristics** | | | | | | | | | | **Methodological considerations** | |  |
| --- | --- | --- | --- | --- | --- | --- | --- | --- | --- | --- | --- | --- | --- | --- | --- | --- | --- | --- | --- | --- | --- | --- | --- | --- | --- |
| **Study** | **Published article** | **Funding** | **Design** | **Region** | **Data source** | **Study enrolment period** | **Dominant SARS-CoV-2 variant** | **Eligibility criteria** | **Risk factors for progression to severe COVID-19** | **Intervention** | **Comparator** | **Initiation window** | **Participants (no.)** | | **Unvaccinated (%)** | | **Age (mean [SD] or median [IQR])** | | **Male (%)** | | **Female (%)** | | **Control of confounding and selection bias** | **Handling of COVID-19 vaccination status** | **Handling of prior SARS-CoV-2 infection** |
|  |  |  |  |  |  |  |  |  |  |  |  |  | **Int** | **Comp** | **Int** | **Comp** | **Int** | **Comp** | **Int** | **Comp** | **Int** | **Comp** |  |  |  |
| Aggarwal 2023 | Yes | US National Institutes of Health | Retrospective cohort | USA | Electronic health records of University of Colorado Health | March 26 to August 25, 2022 | Omicron (BA.2, BA2.12.1, BA.4 and BA.5) | • Age ≥ 18 years at the time of a positive SARS-CoV-2 test result (SARS-CoV-2 polymerase chain reaction or antigen test) or a prescription order for nirmatrelvir/ritonavir (if test result was unavailable) during the study period • Alive and not hospitalized on the test-positive date or date of nirmatrelvir/ritonavir order • Not treated with other outpatient COVID-19 treatments (molnupiravir, bebtelovimab, sotrovimab, tixagevimab/cilgavimab, remdesivir) within 10 days of the test-positive date • Persons were excluded if the test-positive date was > 10 days prior to date of nirmatrelvir/ritonavir order | Not required for cohort entry. Assessed in sensitivity analysis that included individuals with EUA qualifying conditions in the electronic health record data • Immunocompromised • Diabetes mellitus • Cardiovascular disease • Pulmonary disease • Renal disease • Hypertension | Nirmatrelvir/ritonavir | No SARS-CoV-2 treatment | Within 10 days after a SARS-CoV-2 test-positive date | 7168 | 9361 | 20.4 | 21.7 | NR (32% ≥ 65 years) | NR (21% ≥ 65 years) | 41.4 | 41.7 | 58.6 | 58.3 | Propensity score matching and regression adjustment for variables with a standardized mean difference > 0.1 | Number of doses (0, 1, 2, ≥ 3) prior to SARS-CoV-2 test-positive date were included as a covariate in the propensity score model. Stratified analyses by three level vaccination status (0, 1-2, ≥ 3). | NR |
| Bajema 2023 | Yes | US Department of Veterans Affairs | Target trial emulation | USA | Electronic health records from the Veterans Health Administration COVID-19 Shared Data Resource | January 1 to July 31, 2022 | Omicron (B.1.1.529 and BA1.1) | • Age ≥ 18 years at the time of first positive SARS-CoV-2 test result (nucleic acid amplification or antigen test in a respiratory specimen) during the study period • VHA enrolees with a VHA primary care encounter in the 18 months preceding the test-positive date • Alive and not hospitalized within 7 days before through the day following the test-positive date • Not treated with other outpatient COVID-19 treatments (nirmatrelvir-ritonavir, molnupiravir, bebtelovimab, sotrovimab, casirivimab/imdevimab, bamlanivimab/etesevimab, remdesivir) on or prior to the antiviral treatment date • Nirmatrelvir trial: excluded persons with moderate or severe liver disease, advanced renal impairment or a prescription for a contraindicated medication in the 90 days prior to test-positive date  • Molnupiravir trial: excluded pregnant persons • Having ≥ 1 risk factors for progression to severe COVID-19 by the FDA EUA/CDC criteria | Required for cohort entry: • Age ≥ 65 years • Chronic medical conditions (e.g., cardiovascular disease, cancer, chronic liver disease, diabetes) • Pregnancy • Overweight or obese • Immunosuppressive medications or cancer therapies • Current or former tobacco use • Substance or alcohol dependence | Nirmatrelvir/ritonavir | No SARS-CoV-2 treatment | Within 5 days of SARS-CoV-2 test-positive date | 9607 | 9607 | 17.2 | 17.9 | 66 (53–74) | 66 (54–74) | 85.8 | 86.4 | 14.2 | 13.6 | Exact and propensity score matching | Vaccination status (unvaccinated, partial, primary > 4 months, primary 0-4 months, booster > 4 months, booster 0-4 months, other) was included as a covariate in the propensity score model. Vaccination status was defined as follows: • Unvaccinated: did not receive any COVID-19 vaccine or received a vaccine dose other than Janssen < 14 days prior to the first positive test date • Partial: in non-immunocompromised patients, receipt of a single mRNA dose (Pfizer-BioNTech or Moderna) or a single Novavax dose alone or in combination with another vaccine other than Janssen < 14 days prior to the index date or a Janssen (Johnson & Johnson) dose < 14 days before the test date. In immunocompromised patients, receipt of 2 doses of an mRNA vaccine, a single dose of Janssen, or a single dose of Novavax. It was also indicated by receipt of 3 doses of an mRNA vaccine, a single dose of Janssen followed by a single dose of an mRNA vaccine, or 2 doses of Novavax < 7 days before the test date. • Primary: in non-immunocompromised patients, receipt of 2 doses of any mRNA or Novavax vaccine or a single dose of Janssen ≥ 14 days before the test date. In immunocompromised patients, receipt of 3 doses of an mRNA vaccine, a single dose of Janssen followed by a single dose of an mRNA vaccine, or 2 doses of Novavax ≥ 7 days before the test date. • Booster: any primary regimen (appropriate for immune status) followed by an additional dose(s) of mRNA, Janssen, or Novavax vaccine ≥ 7 days before the test date.  • Other: any vaccination not captured in the other vaccination status definitions. | All patients had a first positive laboratory-confirmed SARS-CoV-2 infection. |
|  |  |  |  |  |  |  |  |  |  | Molnupiravir | No SARS-CoV-2 treatment |  | 3504 | 3504 | 14.4 | 15.2 | 70.0 (60.0–75.0) | 70.0 (60.0–75.0) | 91.2 | 91.2 | 8.8 | 8.8 |  |  |  |
| Bhatia 2023 | Preprint | National COVID Cohort Collaborative (N3C) IDeA CTR Collaboration | Target trial emulation | USA | Electronic health record data from the National Institute of Health’s National COVID Cohort Collaborative | December 23, 2021 to December 31, 2022 | Omicron | • Age ≥ 18 years at the time of the COVID-19 index date (either COVID-19 diagnosis or a positive SARS-CoV-2 test result) during the study period • Having ≥1 risk factor for progression to severe COVID-19 as per the CDC criteria • Not hospitalized on or before the COVID-19 index date or  date of treatment or one day before with nirmatrelvir • Received nirmatrelvir on or between five days after the COVID-19 index date | Required for cohort entry: • Age ≥ 50 years • Underlying medical conditions associated with a higher risk of severe COVID-19 | Nirmatrelvir/ritonavir | Never treated with nirmatrelvir/ritonavir | On or within 5 days after the COVID-19 index date | 104510 | 306132 | NR | NR | NR (47% ≥ 65 years) | NR (42% ≥ 65 years) | 38.8 | 38.1 | 61.2 | 61.9 | Coarsened exact matching and regression adjustment | Modified cohort of patients from sites with reliable information on COVID-19 vaccination status (unvaccinated, vaccinated). Same as primary analysis with adjustment for the independent effect of vaccination status and the interaction effect of vaccination and treatment. Unvaccinated was defined as having received 0 doses at index date and vaccinated was defined as ≥ 2 doses at least 14 days prior to index date. | NR |
| Butt 2023 (molnupiravir) | Yes | None | Retrospective cohort | USA | Electronic health records from the Veterans Health Administration COVID-19 Shared Data Resource | January 1 to August 31, 2022 | NR | • Enrolled in VA care with at least 2 visits in the previous 2 years • First confirmed SARS-CoV-2 infection during the study enrolment period • No record of hospitalisation within the 60 days prior or 3 days after the diagnosis of COVID-19 • Excluded if received nirmatrelvir/ritonavir during the study period or molnupiravir > 3 days after the index date. | Not required for cohort entry. • Age ≥ 65 years • Obesity • Diabetes mellitus • Cancer • Chronic lung disease | Molnupiravir | Not treated with molnupiravir | Within 3 days of the COVID-19 diagnosis date | 1459 | 1459 | 14.2 | 14.2 | 69.4 (60.7–75.3) | 69.6 (60.6–75.1) | 91.8 | 91.8 | 8.2 | 8.2 | Exact matching on age, race, sex, body mass index, Charlson Comorbidity Index, VA facility, and vaccination status | Vaccination status (unvaccinated or primary series incomplete, primary series completed, primary series plus booster) prior to COVID-19 infection were included as a matching variable. Stratified analyses by four levels of vaccination status (unvaccinated or primary series incomplete, primary series completed, primary series plus booster, primary +/- booster, last dose > 3 months ago). | All patients had no prior known SARS-CoV-2 infection |
| Butt 2023 (nirmatrelvir) | Yes | National Institute of Allergy and Infectious Diseases at the National Institutes of Health, VA Pittsburgh Healthcare System and Veterans Health Foundation of Pittsburgh | Retrospective cohort | USA | Electronic health records from the Veterans Health Administration COVID-19 Shared Data Resource | January 1, 2022 to February 25, 2023 | NR | • Enrolled in VA care with at least 2 visits in the previous 2 years • First confirmed SARS-CoV-2 infection during the study enrolment period • Having ≥1 risk factor for progression to severe COVID-19  • No record of hospitalisation within the 90 days prior to the diagnosis of COVID-19 • Excluded if received nirmatrelvir/ritonavir > 3 days after the index date or received molnupiravir, monoclonal antibodies for SARS-CoV-2, or remdesivir • Excluded if missing body mass index, missing test date, had severe disease on presentation, outcome on the index test date or before prescription date for nirmatrelvir/ritonavir | Required for cohort entry: • Age ≥ 65 years • Obesity • Diabetes mellitus • Cancer diagnosis • Chronic lung disease • Cardiovascular disease • Chronic kidney disease | Nirmatrelvir/ritonavir | No SARS-CoV-2 treatment | Within 3 days of the COVID-19 diagnosis date | 7615 | 7615 | 15.3 | 15.1 | 66.4 (56.3–74.5) | 66.8 (56.7–74.7) | 85.2 | 85.5 | 14.8 | 14.5 | Estimated the propensity score and performed two analytical approaches, inverse probability of treatment weighting and matching on the propensity score. | Vaccination status (unvaccinated or primary series incomplete, primary series completed, primary series plus booster) prior to COVID-19 infection were included as a matching variable. Stratified analyses by four levels of vaccination status (unvaccinated or primary series incomplete, primary series completed (no booster), primary series complete (plus booster), primary series plus booster dose > 3 months ago). | All patients had no prior known SARS-CoV-2 infection |
| Cegolon 2023 | Yes | None | Retrospective cohort | Italy | Clinical records of patients referred to outpatient infectious disease service of Trieste, Italy | February 1, 2022 to May 31, 2022 | Omicron (BA.1 and BA.2) | • Patients referred to the clinic were considered at high-risk of progressing to severe COVID-19 based on their comorbidities or being immunocompromised | Required for cohort entry. Not explicitly listed in the paper, but some baseline characteristics reported included: • Age ≥ 70 years • Immunocompromised | Nirmatrelvir/ritonavir | Refused antiviral or no treatment because of late referral (>5 days after symptom onset) | Within 5 days of symptom onset | 102 | 111 | 20.2 | 21.7 | 66.2 (15.4) | 70.9 (14.5) | 48.0 | 50.4 | 52.0 | 49.6 | Multivariable logistic regression | Number of doses of COVID-19 vaccine (0–4) was included as a covariate in the regression model | NR |
|  |  |  |  |  |  |  |  |  |  | Molnupiravir | Refused antiviral or no treatment because of late referral (>5 days after symptom onset) |  | 116 | 111 | 20.9 | 21.7 | 66.2 (18.0) | 70.9 (14.5) | 53.5 | 50.4 | 46.5 | 49.6 |  |  |  |
| Dormuth 2023 | Yes | BC Ministry of Health | Retrospective cohort | Canada | Administrative and public health databases (e.g., BC PharmaNet, Medical Services Plan, Discharge Abstract Database) | February 1, 2022 to February 3, 2023 | Omicron | • Age ≥ 18 years at the time of the time of index SARS-CoV-2 test-positive date (polymerase chain reaction) or a prescription for nirmatrelvir/ritonavir during the study period• Met the criteria for one of four mutually exclusive clinically vulnerable groups who were at high risk of complications from COVID-19• Had 730 days of continuous enrolment in the BC Medical Services Plan• Excluded patients with missing age, severe kidney or liver disease, prior use of remdesivir, hospital admission or separation within 30 days prior to cohort entry date, a diagnosis code for pregnancy 730 days prior to cohort entry date | Required for cohort entry.Cohort 1: Severely immunosuppressed individuals • Solid organ transplant• Treated for malignant hematologic conditions• Bone marrow, stem cell transplant or transplant-related immunosuppressant use• Anti-CD20 agents or B-cell depleting agents• Severe primary immunodeficienciesCohort 2: Moderately immunosuppressed individuals• Treatment for cancer including solid tumours• Significantly immunosuppressing drugs• Advanced untreated HIV infection or treated HIV• Moderate primary immunodeficiencies • Renal conditionsGroup 3: Individuals with high-risk conditions• Severe respiratory disorders• Rare blood disorders• Splenectomy (anatomical or functional asplenia)• Diabetes treated with insulin• Haematological and other cancers not captured in cohorts 1 or 2 • Significant developmental disabilities• Pregnant with a serious heart disease• Neurological or other conditions causing significant muscle weakness around lungsCohort 4: Expanded Eligibility• Unvaccinated and age ≥ 50 years• Unvaccinated and ≥ 3 comorbidities• Age ≥ 50 years, 1-2 vaccine doses or previous infection, and ≥ 3 comorbidities• Age ≥ 70 years, 1-2 vaccine doses or previous infection, and ≥ 1 comorbidities• Age ≥ 70 years regardless of vaccination and ≥ 3 comorbidities• Indigenous individuals who meet at least one of the following: unvaccinated without previous infection, ≥ 50 years with 1-2 vaccine doses or previous infection alone, or ≥ 70 years regardless of vaccine status or previous infection | Nirmatrelvir/ritonavir | Not treated with nirmatrelvir/ritonavir or remdesivir (and had a positive SARS-CoV-2 test) | Within 5 days after SARS-CoV-2 positive test (for intervention group if test is missing imputed as 3 days) | 1050 | 1050 | 5.5 | 4.5 | 73.0 (59.0–83.0) | 73.0 (59.0–83.0) | 47.6 | 47.6 | 52.4 | 52.4 | Matching on age, sex, high-dimensional propensity score, year and calendar month of cohort entry | Likely included in the propensity score model | Likely included in the propensity score model |
| Dryden-Peterson 2023 | Yes | National Institutes of Health | Target trial emulation | USA | Electronic health records from Mass General Brigham, an integrated health system in Massachusetts and southern New Hampshire | January 1 to July 17, 2022 | Omicron (BA.1.1, BA.2, BA.2.12.1, and BA.5) | • Age ≥ 50 years at the time of a positive SARS-CoV-2 test result (SARS-CoV-2 polymerase chain reaction or antigen test) and resided in Massachusetts or New Hampshire  • New diagnosis of COVID-19, defined as no positive molecular test result in the preceding 90 days, as an outpatient • Excluded persons with renal impairment (estimated glomerular filtration rate < 30mL/min in the prior year), a prescription for a contraindicated medication or no recorded weight in past two years • Alive and not hospitalized at the end of the second calendar day after the date of the positive SARS-CoV-2 test result • Not treated with other COVID-19 treatments (sotrovimab, bebtelovimab, molnupiravir, or remdesivir) within 1 calendar day of the test-positive date | Not required for cohort entry. • Age ≥ 65 years • Obesity • Immunocompromised • Diabetes mellitus • Solid tumour • Rheumatologic or inflammatory bowel disease • Pulmonary disease | Nirmatrelvir/ritonavir | No SARS-CoV-2 treatment | Up to 1 calendar day after SARS-CoV-2 positive test | 11797 | 32248 | 7 | 8 | NR (46% ≥ 65 years) | NR (44% ≥ 65 years) | 41.0 | 39.0 | 59.0 | 61.0 | Inverse probability weights estimated using a logistic regression model | Vaccination status (unvaccinated, partially vaccinated, vaccinated, vaccinated and ≥ 1 booster dose) and time since last vaccine dose (< 20 weeks, > 20 weeks) were included as covariates in the propensity score model | NR |
| Evans 2023 | Yes | Health and Care Research Wales on behalf of the Welsh Government | Retrospective cohort | Wales | Electronic health record data from the Secure Anonymised Information Linkage Databank | December 16, 2021 to April 22, 2022 | Omicron (BA.1 and BA.2) | • Age ≥ 18 years at the time of the SARS-CoV-2 test-positive date (polymerase chain reaction or lateral flow device) during the study period • Included in one or more of the ten cohorts considered to be at higher risk from COVID-19 in accordance with the UK clinical access policy and eligible for treatment with sotrovimab molnupiravir, or nirmatrelvir/ritonavir • Not hospitalized on the COVID-19 test-positive date • Received treatment on or between seven days after the COVID-19 test-positive date • Excluded if missing key demographic information, treated with casirivimab/imdevimab, or treated and no record of a positive COVID-19 test in the data sources | Required for cohort entry: • Chromosomal disorders affecting the immune system, including Down syndrome • Certain types of cancer and treatment • Sickle cell disease • Certain conditions affecting their blood • Chronic kidney disease (stages 4 or 5) • Severe liver disease • Organ transplant • Certain autoimmune or inflammatory conditions (e.g., rheumatoid arthritis, inflammatory bowel disease) • Human immunodeficiency virus or acquired immunodeficiency syndrome • Inherited or acquired states affecting the immune system | Nirmatrelvir/ritonavir | Not treated with nirmatrelvir/ritonavir, molnupiravir or sotrovimab | Within 7 days of testing positive for SARS-CoV-2 | 602 | 4973 | 1.7 | 4.4 | 50 (14) | 57 (18) | 35.2 | 46.4 | 64.8 | 53.6 | Cox proportional hazards regression with time-dependent covariate for treatment | Number of vaccine doses (unvaccinated, 1-3 doses, ≥ 4 doses) was included as a covariate in the regression model | NR |
|  |  |  |  |  |  |  |  |  |  | Molnupiravir | Not treated with nirmatrelvir/ritonavir, molnupiravir or sotrovimab |  | 359 | 4973 | 3.1 | 4.4 | 56 (16) | 57 (18) | 39.0 | 46.4 | 61.0 | 53.6 |  |  |  |
| Faust 2023 | Yes | None | Retrospective cohort | Multinational | Electronic health records from TriNetX Analytics Network database—Research Network, a multicentre federated health research network | December 1, 2021 to July 30, 2022 | Omicron | • Age 18–50 years who were vaccinated and subsequently tested positive for SARS-CoV-2 or were diagnosed with COVID-19 at least one month after vaccination • On the index event could not have an inpatient encounter and a record of treatment with an anti-SARS-CoV-2 monoclonal antibody, convalescent plasma, or molnupiravir | Not required for cohort entry • Hypertension • Diabetes mellitus • Chronic lower respiratory disease • Cancer • BMI ≥ 30 kg/m2 • Cardiovascular disease | Nirmatrelvir/ritonavir | Not treated with nirmatrelvir/ritonavir, anti-SARS-CoV-2 monoclonal antibody, convalescent plasma, or molnupiravir | Within 5 days of testing positive for SARS-CoV-2 or being diagnosed with COVID-19 | 2547 | 2547 | 0 | 0 | 37.6 (8.3) | 37.7 (8.5) | 32.3 | 33.4 | 67.7 | 66.6 | Propensity score matching | All study participants were vaccinated | NR |
| Ganatra 2023 | Yes | None | Retrospective cohort | Multinational | Electronic health records from TriNetX Analytics Network database—Research Network, a multicentre federated health research network | December 1, 2021 to April 18, 2022 | Omicron | • Age ≥ 18 years who were vaccinated and subsequently tested positive for SARS-CoV-2 or were diagnosed with COVID-19 at least one month after vaccination • On the index event could not have an inpatient encounter and a record of treatment with an anti-SARS-CoV-2 monoclonal antibody, convalescent plasma, or molnupiravir | Not required for cohort entry. • Hypertension • Diabetes mellitus • Chronic lower respiratory disease • Malignancy • Chronic kidney disease • BMI ≥ 30 kg/m^2^ • Chronic heart conditions | Nirmatrelvir/ritonavir | Not treated with nirmatrelvir/ritonavir, anti-SARS-CoV-2 monoclonal antibody, convalescent plasma, or molnupiravir | Within 5 days of testing positive for SARS-CoV-2 or being diagnosed with COVID-19 | 1130 | 1130 | 0 | 0 | 57.5 (16.3) | 57.7 (16.3) | 37.0 | 35.9 | 63.0 | 64.1 | Propensity score matching | All study participants were vaccinated | NR |
| Gmizic 2023 | Preprint | None | Retrospective cohort | Serbia | Electronic health records from the Clinic for Infectious and Tropical Diseases, University Clinical Center in Belgrade, Serbia | December 15, 2021 to February 15, 2022 | Omicron | • Age ≥ 18 years and non-hospitalized with mild or moderate COVID-19 (positive test for real-time reverse transcriptase polymerase chain reaction or an antigen test) • At least one risk factor for the development of severe illness from COVID-19 | Required for cohort entry: • Age > 60 years • Active cancer • Chronic kidney disease • Chronic obstructive pulmonary disease • Obesity • Serious heart conditions (i.e., heart failure, coronary artery disease, or cardiomyopathies) • Diabetes mellitus | Molnupiravir | Not treated with molnupiravir | Within 5 days of symptom onset | 165 | 155 | 27.3 | 49.0 | 64 (13) | 66 (16) | 48.5 | 51.0 | 51.5 | 49.0 | Multivariable Cox proportional hazards regression | Number of vaccine doses and vaccine type were included as a covariate in the regression model | NR |
| Kwok 2023 | Yes | Department of Microbiology, The University of Hong Kong | Retrospective cohort | Hong Kong | Electronic health records from the Hospital Authority | February 26 to August 26, 2022 | Omicron | • Age ≥ 18 years, who were not vaccinated against COVID-19 and had chronic respiratory diseases, including asthma, COPD and bronchiectasis, and treated in an outpatient setting • Patients who did not have a vaccination record or who had past COVID-19 before the study start date were excluded | Not required for cohort entry. • Diabetes mellitus • Being obese with body mass index more than 30 kg/m^2^ • ≥ 60 years • Immunocompromised state or having underlying chronic illnesses | Molnupiravir | Not treated with other antiviral treatment against COVID-19 | Within 5 days of symptom onset | 578 | 2387 | 100 | 100 | 84 (75–90) | 82 (73–89) | 66.8 | 73.8 | 33.2 | 26.2 | Multivariable log-binomial regression | All study participants were unvaccinated | Study participants with prior SARS-CoV-2 infection were excluded |
|  |  |  |  |  |  |  |  |  |  | Nirmatrelvir/ritonavir | Not treated with other antiviral treatment against COVID-19 |  | 302 | 2387 | 100 | 100 | 79 (71–87) | 82 (73–89) | 63.9 | 73.8 | 36.1 | 26.2 |  |  |  |
| Lewnard 2023 | Yes | US CDC and National Institutes of Health | Retrospective cohort | USA | Electronic health records from Kaiser Permanente Southern California | April 8 to October 7, 2022 | Omicron (BA.2, BA.4, BA.5) | • Age ≥ 12 years at the time of the positive SARS-CoV-2 polymerase chain reaction test result (index test)* • No positive test result within the preceding 90 days of the index test • Not hospitalised at the time of their index test or within the preceding 7 days • Were continuously enrolled in Kaiser Permanente Southern California health plans for at least 1 year before their index test | Not required for cohort entry. | Nirmatrelvir/ritonavir | Not treated with nirmatrelvir/ritonavir | Within 5 days of onset of symptoms or at any time after testing positive for SARS-CoV-2 (irrespective of the presence or timing of symptoms) | 7274 | 126152 | 5.4 | 13.3 | NR (54% ≥ 60 years) | NR (25% ≥ 60 years) | 42.3 | 44.7 | 57.7 | 55.3 | Matching and multivariable Cox proportional hazards regression | Number of vaccine doses (0, 1, 2, 3, 4) included as a strata (match) in the regression model | Previous SARS-CoV-2 infection (any documented infection, no documented infection) included as a covariate in the regression model |
| Liu 2023 | Yes | None | Retrospective cohort | Multinational | Electronic health records from TriNetX Analytics Network database—Research Network, a multicentre federated health research network | March 1, 2020 to January 1, 2023 | NR | • Age ≥ 18 years with a diagnosis of substance use disorder who had a positive test result for SARS-CoV-2 or a diagnosis of COVID-19 • At least 2 medical encounters with a health care organization during the cohort enrolment period • On the index event could not have an inpatient encounter and a record of treatment with an anti-SARS-CoV-2 monoclonal antibody, convalescent plasma, or molnupiravir | Not required for cohort entry | Nirmatrelvir/ritonavir | Not treated with nirmatrelvir/ritonavir, anti-SARS-CoV-2 monoclonal antibody, convalescent plasma, or molnupiravir | Within 5 days of testing positive for SARS-CoV-2 or being diagnosed with COVID-19 | 10601 | 10601 | NR | NR | 54.0 (15.4) | 54.4 (16.0) | 42.0 | 42.0 | 58.0 | 58.0 | Propensity score matching | Not included as a covariate in the propensity score model but assessed in stratified analyses based on number of COVID-19 vaccine doses (<2 doses, ≥ 2 doses) | NR |
| Low 2023 | Yes | Ministry of Health Malaysia Research Grant for Communicable Diseases | Retrospective cohort | Malaysia | MySejahtera eCOVID system from 647 public health clinics and hospitalisation databases from the Ministry of Health | July 14 to November 14, 2022 | Omicron (BA.4, BA.5, and XBB) | • Age ≥ 18 years who were symptomatic and tested positive for SARS-CoV-2 in a public health clinic during the study period • Not pregnant, no diagnosis of stage 4 or 5 chronic kidney disease or kidney transplant • Complete data on disease category and day of illness • Excluded if >5 days after symptom onset or had COVID-19 pneumonia requiring supplemental oxygen, critical illness or planned hospital admission | Not required for cohort entry: • Age ≥ 60 years • Diabetes mellitus • Hypertension • Respiratory disease | Nirmatrelvir/ritonavir | Not treated with nirmatrelvir/ritonavir | Within 5 days of symptom onset | 10483 | 10483 | 1.8 | 1.6 | 48.5 (16.6) | 47.2 (16.5) | 50.3 | 42.6 | 49.7 | 57.4 | Propensity score matching | Vaccination status (partially vaccinated, fully vaccinated/booster) was included as a covariate in the propensity score model | NR |
| Lui 2023 | Yes | Health and Medical Research Fund, Health Bureau, Government of Hong Kong Special Administrative Region, China | Retrospective cohort | Hong Kong | Electronic health records from the Hospital Authority and Department of Health | February 26 to October 23, 2022 | Omicron | • Age ≥ 18 years with confirmed SARS-CoV-2 infection (polymerase chain reaction or positive rapid antigen test) and type 2 diabetes mellitus • Not hospitalized, not in a residential care home for the elderly and alive on the earliest of date of symptom onset or COVID-19 confirmation (index date) • No severe renal disease, no liver disease, no missing demographics (age or sex) or no prescription record for a medication contraindicated for use with nirmatrelvir/ritonavir • Initiated intervention within 5 days of index date and no documented outpatient use ever of the other oral antiviral drug | Not required for cohort entry. • Aged > 65 years • Underlying chronic illnesses (as measured by the Charlson comorbidity index) • Not fully vaccinated | Molnupiravir | Not treated with oral antiviral drugs in the outpatient setting | Within 5 days of symptom onset or the date of confirmed SARS-CoV-2 infection | 921 | 921 | 58.0 | 56.2 | 76.7 (10.8) | 76.6 (11.7) | 52.9 | 52.3 | 47.1 | 47.7 | Propensity score matching | Vaccinated status (not fully vaccinated, fully vaccinated (not booster), fully vaccinated and boosted) included as a covariate in the propensity score model. Fully vaccinated but not boosted was defined as at least two doses of BNT162b2 (Fosun–BioNTech) or three doses of CoronaVac (Sinovac). Fully vaccinated and boosted was defined as having at least 3 doses of mRNA vaccine, BNT162b2 (Fosun–BioNTech), or 4 doses of inactivated vaccine, CoronaVac (Sinovac). Stratified estimates reported by vaccination status (fully vaccinated, not-fully vaccinated/unvaccinated). | NR |
|  |  |  |  |  |  |  |  |  |  | Nirmatrelvir/ritonavir | Not treated with oral antiviral drugs in the outpatient setting | Within 5 days of symptom onset or the date of confirmed SARS-CoV-2 infection | 793 | 793 | 41.5 | 41.9 | 71.7 (11.5) | 71.9 (11.6) | 50.6 | 49.8 | 49.4 | 50.2 |  |  |  |
| Najjar-Debbiny 2023(molnupiravir) | Yes | None | Retrospective cohort | Israel | Electronic health records from Clalit Health Services and the Israeli Ministry of Health COVID-19 database | January 1 to February 28, 2022 | Omicron | • Age ≥ 18 years with first ever positive test for SARS-CoV-2 (polymerase chain reaction or antigen tests) and who were not hospitalized on the test-positive date• At least 1 comorbidity associated with high risk for severe COVID-19• Excluded patients with contraindications to molnupiravir (i.e., estimated glomerular filtration rate < 30 mL/min/1.73 m^2^, dialysis, pregnancy, platelet count < 100,000/mm^3^, neutrophil count < 500/mm^3^, chronic pancreatitis or acute pancreatitis in the prior 3 months)• Excluded patients treated with nirmatrelvir/ritonavir and patients who received molnupiravir more than 5 days after the SARS-CoV-2 positive test date | At least one required for cohort entry:• Age ≥ 60 years• Body mass index (BMI) ≥ 30 kg/m^2^• Diabetes• Hypertension• Cardiovascular disease• Chronic liver disease• Chronic lung disease• Chronic kidney disease• Neurological disease• Immunosuppression• Malignancy | Molnupiravir | Not treated with other COVID-19 treatments (monoclonal antibodies or nirmatrelvir/ritonavir) | Within 5 days of testing positive for SARS-CoV-2 | 2661 | 2661 | 22.7* | 22.7* | 73.1 (11.7) | 73.1 (11.6) | 50.4 | 49.8 | 49.6 | 50.2 | Exact matching on date of positive test and propensity score matching | Vaccination status (nonadequate*, adequate) included as a variable in the propensity score model. Nonadequate vaccination was defined as only 1 dose of vaccination or ≥ 2 doses but > 180 days passed since the last vaccine dose. For the second vaccine dose and subsequent doses given more than 180 days apart, a patient was considered to be adequately vaccinated if they received the last dose in the prior 8-180 days. If the gap between the last two doses was less than 180 days, a patient was considered adequately vaccinated starting from the date of the last vaccine dose up to 180 days after. | NR |
| Najjar-Debbiny 2023 (nirmatrelvir) | Yes | None | Retrospective cohort | Israel | Electronic health records from Clalit Health Services and the Israeli Ministry of Health COVID-19 database | January 1 to February 28, 2022 | Omicron | • Age ≥ 18 years with first ever positive test for SARS-CoV-2 (polymerase chain reaction or antigen tests) and who were not hospitalized on the test-positive date • At least 1 comorbidity associated with high risk for severe COVID-19 • Excluded patients with contraindications to nirmatrelvir/ritonavir (i.e., a prescription for a contraindicated medication, estimated glomerular filtration rate < 30 mL/min/1.73 m^2^, dialysis, weight < 40 kg or pregnancy) • Excluded patients treated with molnupiravir and patients who received nirmatrelvir/ritonavir > 5 days after the SARS-CoV-2 positive test date | At least one required for cohort entry: • Age ≥ 60 years • Body mass index (BMI) ≥30 kg/m^2^ • Diabetes • Hypertension • Cardiovascular disease • Chronic liver disease • Chronic lung disease • Chronic kidney disease • Neurological disease • Immunosuppression • Malignancy | Nirmatrelvir/ritonavir | Not treated with other COVID-19 treatments (monoclonal antibodies or molnupiravir) | Within 5 days of testing positive for SARS-CoV-2 | 4737 | 175614 | 22.2* | 25.0* | 68.5 (12.5) | 53.9 (16.8) | 42.1 | 41.0 | 57.9 | 59.0 | Cox proportional hazards regression with time-dependent covariate for treatment | Vaccination status (nonadequate*, adequate) included as a baseline covariate in the regression model. Nonadequate vaccination was defined as only 1 dose of vaccination or ≥ 2 doses but > 180 days passed since the last vaccine dose. For the second vaccine dose and subsequent doses given more than 180 days apart, a patient was considered to be adequately vaccinated if they received the last dose in the prior 8-180 days. If the gap between the last two doses was less than 180 days, a patient was considered adequately vaccinated starting from the date of the last vaccine dose up to 180 days after. | NR |
| Paraskevis 2023 | Yes | None | Retrospective cohort | Greece | COVID-19 national registry and SARS-CoV-2 surveillance data maintained by the National Public Health Organization | February 2 to July 20, 2022 | Omicron (BA.1, and BA.2) | • Age ≥ 65 years with SARS-CoV-2 infection (positive nucleic acid amplification or rapid antigen test) and who were not hospitalized | Not required for cohort entry: • Immunocompromised secondary to a disease or medication • Haemodialysis • Cystic fibrosis • Age ≥ 60 years with at least one chronic comorbidity | Molnupiravir | Not treated with other antiviral treatment against SARS-CoV-2 | Within 5 days of symptom onset or positive SARS-CoV-2 test date | 4240 | 4240 | 12.6 | 20.3 | NR (100% ≥ 65 years) | NR (100% ≥ 65 years) | 50.2 | 50.2 | 49.8 | 49.8 | Multivariable logistic regression | Vaccination status (non-vaccinated, vaccinated [2, 3, or 4 doses ≤ 6 months before index infection], vaccinated [2, 3, or 4 doses > 6 months before index infection]) were included as a covariate in the regression model | Previous SARS-CoV-2 infection (yes, no) included as a covariate in the regression model |
|  |  |  |  |  |  |  |  |  |  | Nirmatrelvir/ritonavir | Not treated with other antiviral treatment against SARS-CoV-2 |  | 13861 | 13861 | 9.5 | 15 | NR (100% ≥ 65 years) | NR (100% ≥ 65 years) | 48.1 | 46.6 | 51.9 | 53.4 |  |  |  |
| Petrakis 2023 | Yes | None | Retrospective cohort | Greece | Electronic health records from the Clinic of Infectious Diseases of the University General Hospital of Alexandroupolis | March 1, 2022 to March 1, 2023 | Omicron (BA.2, BA2.12.1, BA.4, and BA.5) | • Age ≥18 years and had confirmed SARS-CoV-2 infection as outpatient • Experienced symptom onset no more than 5 days before drug administration • At least 1 comorbidity associated with high risk for severe COVID-19 • Excluded patients with anticipated hospitalisation within 48 hours, severe drug interactions with concomitant medications or those being treated with alternative antiviral agents | At least one required for cohort entry: • Immunosuppression secondary to disease or medication • Malignancies • Chronic renal disease, • Chronic respiratory disease • Cystic fibrosis • Age ≥ 75 • Age > 65 plus one chronic comorbidity • Age < 65 plus 2 chronic comorbidities | Nirmatrelvir/ritonavir | Not treated with other antiviral treatment against SARS-CoV-2 | Within 5 days of symptom onset | 200 | 200 | 10.0 | 7.0 | 75.2 (13.1) | 76.9 (14.0) | 59.0 | 60.5 | 41.0 | 39.5 | Exact matching and multivariable logistic regression | Vaccination status (unvaccinated, incomplete vaccination [1 dose, 2 doses, 1st booster dose], complete vaccination [2 or more booster doses]) was included as a covariate in the regression model | Previous SARS-CoV-2 infection (yes, no) included as a covariate in the regression model |
| Schwartz 2023 | Yes | Public Health Ontario | Retrospective cohort | Canada | Insurance claims and public health databases (e.g., Ontario Drug Benefit (ODB), COVAXON, Canadian Institute for Health Information (CIHI) and Ontario Health Insurance Plan (OHIP) databases) | April 4 to August 31, 2022 | Omicron | • Age 18-110 years with a positive polymerase chain reaction test for SARS-CoV-2 during the study period and were a resident of Ontario • Excluded patients dispensed nirmatrelvir/ritonavir before the test-positive date, with invalid identifiers such as date of birth or death before the test date, date of hospital admission or death on or before the test-positive date | Not required for cohort entry. • Age 70 and older • Diabetes • Chronic heart disease • Hypertension • Chronic respiratory disease • Dementia • Chronic kidney disease • Advanced liver disease • Immunocompromised | Nirmatrelvir/ritonavir | Not treated with nirmatrelvir/ritonavir | Within 5 days of positive SARS-CoV-2 test date | 8876 | 168669 | 5.3 | 6.2 | 74.3 (16.3) | 52.4 (21.0) | 40.7 | 36.6 | 59.3 | 63.4 | Inverse probability of treatment weighting based on the estimated propensity score using a logistic regression model. Index date was randomly assigned (imputed) for comparator group. | Number of doses (0, 1, 2, ≥ 3) and time since last vaccine dose (14-89, 90-179, 180-269, ≥ 270 days) were included as covariates in the propensity score model | Previous SARS-CoV-2 infection (yes, no) included as a covariate in the propensity score model |
| Shah 2023 | Yes | None | Retrospective cohort | USA | Electronic health records in Cosmos (US health systems covered by Epic software) | April 1 to August 31, 2022 | Omicron | • Age ≥ 50 years (or ≥ 18 years and older with a documented underlying health condition) with a diagnosis of COVID-19 or a positive SARS-CoV-2 test result (nucleic acid amplification or antigen test in a respiratory specimen) during the study period • An outpatient encounter associated with the COVID-19 diagnosis • At least one previous face-to-face encounter in Cosmos during the 3 years preceding the COVID-19 diagnosis • Excluded patients who were pregnant, had a contraindicated medication in the preceding 6 months, had severe hepatic or renal impairment, received nirmatrelvir/ritonavir 90 days before or 6-30 days after the COVID-19 diagnosis date or the patient received other SARS-CoV-2 specific treatments (within 30 days of diagnosis) | Required for cohort entry: • Age ≥ 50 years • Age ≥ 18 years and older with a documented underlying health condition as per the CDC guidelines Other health conditions reported: • Immunocompromised • Obesity • Smoking • Diabetes mellitus | Nirmatrelvir/ritonavir | Not treated with other antiviral treatment against SARS-CoV-2 | Within 5 days of COVID-19 diagnosis or positive SARS-CoV-2 test date | 198927 | 500921 | 15.4 | 28.3 | NR (38% ≥ 65 years) | NR (27% ≥ 65 years) | 38.2 | 36.8 | 61.8 | 63.2 | Cox proportional hazards regression with time-dependent covariate for treatment | Vaccination status was included as a covariate in the regression model (unvaccinated, 2 mRNA doses, ≥ 3 mRNA doses, other). Stratified estimates by vaccination status. Vaccination status categories were defined as 1) unvaccinated if no COVID-19 vaccine had been received; 2) 2 mRNA dose-recipients if ≥ 14 days had elapsed after the second dose and no subsequent doses had been received or < 7 days since receipt of third dose; 3) ≥ 3 mRNA dose-recipients if ≥ 7 days had elapsed since receipt of the third dose; and 4) other recipient if any Janssen (Johnson & Johnson) vaccine, other vaccine, or 1 mRNA vaccine dose had been received any time before COVID-19 diagnosis. | Previous infection was included as a covariate in the regression model and was defined as a COVID-19 diagnosis code or positive COVID-19 nucleic acid amplification test result or antigen test result > 90 days before the current diagnosis during the study period |
| Tazare 2023 | Preprint | UK Research and Innovation and Wellcome Trust | Target trial emulation | England | OpenSAFELY platform (English primary care GP records [TPP SystemOne]) National Immunisation Management System (NIMS), NHS Digital’s Hospital Episode Statistics (HES), Second Generation Surveillance System (SGSS), and the “COVID-19 therapeutics dataset” from NHS England | February 11 to May 21, 2023 | Omicron (BA.2) | • Age 18-110 years who tested positive (polymerase chain reaction or lateral flow test) for SARS-CoV-2 during the study period and were registered at a practice using TPP software • Not hospitalised on the day of the positive test • No evidence of SARS-CoV-2 infection in the 90 days before the positive test date • Have no treatment history of antiviral or neutralising monoclonal antibody therapy prior to the positive test date • Not be treated with sotrovimab and molnupiravir on the same day or treatment with (remdesivir or nirmatrelvir/ritonavir) • Excluded patients with missing sex and demographics (including the Sustainability and Transformation Partnership region[NHS administrative region] or index of multiple deprivation) | At least one required for cohort entry: • Diagnosed with Down syndrome • Active or recently treated solid cancer • Haematological disease or stem cell transplant • Renal disease • Liver disease • Immune-mediated inflammatory disorders, immune deficiencies, HIV/AIDS or solid organ transplant • Rare neurological conditions | Molnupiravir | Not treated with other antiviral treatments against SARS-CoV-2 | Within 5 days of positive test result | 1242 | 35028 | 3.9 | 5.9 | NR (55% ≥ 60 years) | NR (56% ≥ 60 years) | 44.4 | 45.3 | 55.1 | 54.7 | Weighted Cox proportional hazards regression and clone-censor-weight approach with inverse probability of censoring weights estimated using pooled logistic regression. | Vaccination status (unvaccinated, unvaccinated (declined), 1 dose, 2 doses, ≥ 3 doses) and most recent vaccine type (Pfizer, AstraZeneca, Moderna) were included as baseline covariates in the censoring models. | Part of the eligibility criteria, not considered in the statistical analysis. |
| Van Heer 2023 | Yes | None | Retrospective cohort | Australia | Administrative and public health databases from the Victorian Department of Health (e.g., Transmission Response Epidemiology Victoria Information System, National Medical Stockpile) and Australian Government (e.g., Australian Immunisation Registry, Pharmaceutical Benefits Scheme) | July 11 to October 31, 2022 | Omicron (BA.4 and BA.5) | • Age ≥ 70 years with confirmed SARS-CoV-2 infection (polymerase chain reaction or positive rapid antigen test) that was reported to the Victorian Department of Health • Record of 1 to 4 doses of COVID-19 vaccine at least 14 days prior to index COVID-19 diagnosis • Excluded if record of receiving remdesivir, tixagevimab/cilgavimab, missing sex, hospitalised within 2 days of COVID-19 diagnosis, and residing in aged care (hospitalisation analysis only) | Not required for cohort entry | Nirmatrelvir/ritonavir | Not treated with other COVID-19 treatments (remdesivir or molnupiravir) | Not restricted, but 97% received treatment within 3 days of COVID-19 diagnosis | 5250 | 13721 | 3.8 (1-2 doses) | 8.3 (1-2 doses) | 76.0 (72.0–81.0) | 77.0 (73.0–84.0) | 43.5 | 47.7 | 56.5 | 52.3 | Multivariable logistic regression | Number of vaccine doses (1-2 doses, 3 doses, 4 doses) was included as a covariate in the regression model | NR |
|  |  |  |  |  |  |  |  |  |  | Molnupiravir | Not treated with other COVID-19 treatments (remdesivir or nirmatrelvir/ritonavir) |  | 19962 | 13721 | 4.2 (1–2 doses) | 8.3 (1–2 doses) | 78.0 (74–85) | 77.0 (73.0–84.0) | 43.7 | 47.7 | 56.3 | 52.3 |  |  |  |
| Wee 2023 | Yes | None | Retrospective cohort | Singapore | Singapore Ministry of Health administrative and health insurance claims databases | March 18 to December 31, 2022 | Omicron (BA.2, BA.4, BA.5, and XBB) | • Age ≥ 60 years with confirmed SARS-CoV-2 infection (polymerase chain reaction or positive rapid antigen test) who were Singapore citizens/permanent residents and presented to primary care clinics • Alive and not hospitalised on the date of the first positive test • Without severe liver disease or end-stage renal disease, not treated with molnupiravir, and missing sociodemographic data | Not required for cohort entry. • Aged > 60 years • Immunocompromised • Chronic comorbidities | Nirmatrelvir/ritonavir | Not treated with oral antiviral drugs in the outpatient setting | Not restricted, but nearly all received treatment on the index date of COVID-19 diagnosis as medications are dispensed in the clinic | 3959 | 139379 | 1.1 | 1.1 | NR (20% ≥ 80 years) | NR (11% ≥ 80 years) | 43.2 | 44.4 | 56.8 | 55.6 | Multivariable logistic regression. In a sensitivity analysis, inverse probability of treatment weighting based on the estimated propensity score using a logistic regression model of all study patients | Vaccination status (unvaccinated, partially vaccinated (1 dose of mRNA vaccine), fully vaccinated (2 doses of mRNA vaccine), boosted (3 doses of mRNA vaccine dose 6–9 months after the second dose), doubly boosted (4 doses of mRNA vaccine with the last dose received 5 months after the first booster) was included as a covariate in the regression model. | Reinfection status included as a covariate in the regression model |
| Wong 2022 | Yes | Health and Medical Research Fund, Health Bureau, Government of Hong Kong Special Administrative Region, China | Retrospective cohort | Hong Kong | Electronic health records from the Hospital Authority and Department of Health | February 26 to June 26, 2022 | Omicron (BA.2) | • Age ≥ 18 years with confirmed SARS-CoV-2 infection (polymerase chain reaction or positive rapid antigen test) • Not hospitalized, not in a residential care home for the elderly and alive on the earliest of date of symptom onset or COVID-19 confirmation (index date) • No severe renal disease, no liver disease, no missing demographics (age or sex) or no prescription record for a medication contraindicated for use with nirmatrelvir/ritonavir • Initiated intervention within 5 days of index date and no documented outpatient use ever of the other oral antiviral drug | Not required for cohort entry. • Aged > 60 years • Underlying chronic illnesses (as measured by the Charlson comorbidity index) • Not fully vaccinated | Molnupiravir | Not treated with oral antiviral drugs in the outpatient setting | Within 5 days of symptom onset or the date of confirmed SARS-CoV-2 infection | 4983 | 49234 | 83.9 | 87.6 | NR (89% > 60 years) | NR (92% > 60 years) | 47.5 | 47.8 | 52.5 | 52.2 | Propensity score matching and Cox proportional hazards regression | Vaccination status (unvaccinated/partially vaccinated, fully vaccinated) included as a covariate in the propensity score model. Fully vaccinated defined as at least two doses of BNT162b2 (Fosun–BioNTech) or three doses of CoronaVac (Sinovac). Stratified estimates reported by vaccination status. | NR |
|  |  |  |  |  |  |  |  |  |  | Nirmatrelvir/ritonavir | Not treated with oral antiviral drugs in the outpatient setting | Within 5 days of symptom onset or the date of confirmed SARS-CoV-2 infection | 5542 | 54672 | 66.6 | 66.8 | NR (86% > 60 years) | NR (85% > 60 years) | 46.3 | 46.6 | 53.7 | 53.4 |  |  |  |
| Wu 2023 | Yes | None | Retrospective cohort | Multinational | Electronic health records from TriNetX Analytics Network database—Research Network, a multicentre federated health research network | January 1, 2020 to December 31, 2022 | NR | • Age ≥ 18 years with a diagnosis of diabetes mellitus who had a positive test result for SARS-CoV-2 or a diagnosis of COVID-19• At least 2 medical encounters with a health care organization during the cohort enrolment period• On the index event could not have an inpatient encounter and a record of treatment with an anti-SARS-CoV-2 monoclonal antibody, convalescent plasma, or molnupiravir• Excluded patients who were hospitalized within 5 days of the positive test result for SARS-CoV-2 or diagnosis of COVID-19 date | Not required for cohort entry | Nirmatrelvir/ritonavir | Not treated with nirmatrelvir/ritonavir, anti-SARS-CoV-2 monoclonal antibody, convalescent plasma, or molnupiravir | Within 5 days of testing positive for SARS-CoV-2 or being diagnosed with COVID-19 | 13822 | 13822 | NR | NR | 61.5 (14.4) | 61.7 (14.9) | 42.7 | 42.5 | 57.3 | 57.5 | Propensity score matching | Not included as a covariate in the propensity score model but assessed in stratified analyses based on number of COVID-19 vaccine doses (unvaccinated, 2 doses, ≥ 3 doses) |  |
| Xie 2023 (molnupiravir) | Yes | US Department of Veterans Affairs | Target trial emulation | USA | Electronic health records from the VHA Corporate Data Warehouse and the COVID-19 Shared Data Resource | January 5 to September 30, 2022 | Omicron (BA.1, BA.2 or BA.5) | • Age ≥ 18 years with a SARS-CoV-2 positive test result and at least one risk factor for developing severe COVID-19 disease • Not hospitalized on the date of positive SARS-CoV-2 test result and no end stage kidney disease or eGFR<30 mL/min/1.73m^2^  • Did not receive other COVID-19 treatments (SARS-CoV-2 antibody or other antiviral drugs) | At least one required for cohort entry: • Age > 60 years • Body mass index > 30 kg/m^2^ • Chronic lung disease • Cancer • Cardiovascular disease • Chronic kidney disease • Diabetes | Molnupiravir | Not treated with other COVID-19 treatments | Within 5 days of SARS-CoV-2 test-positive date | 7818 | 78180 | 14.2 | 17.5 | 69.2 (12.0) | 67.1 (12.7) | 91.2 | 89.4 | 8.8 | 10.6 | Propensity score matching and clone-censor-weight approach with the cumulative inverse probability of censoring weight estimated using a logistic regression model. | Included as a covariate in the propensity score model (not vaccinated, one or two doses, a booster dose). Separately emulated the trial among target populations according to vaccination: 1) not vaccinated, 2) received one or two doses of COVID-19 vaccine, 3) received a booster dose. | Included a history of SARS-CoV-2 infection as a covariate in the propensity score model. Separately emulated the trial among target populations according to history of SARS-CoV-2 infection: 1) had a history of SARS-CoV-2 infection or 2) no history of SARS-CoV-2 infection. |
| Xie 2023 (nirmatrelvir) | Yes | US Department of Veterans Affairs | Target trial emulation | USA | Electronic health records from the VHA Corporate Data Warehouse and the COVID-19 Shared Data Resource | January 3 to November 30, 2022 | Omicron (BA.1, BA.2 or BA.5) | • Age ≥ 18 years with a SARS-CoV-2 positive test result and at least one risk factor for developing severe COVID-19 disease • Not hospitalized on the date of positive SARS-CoV-2 test result, no liver disease, no kidney disease, or no medication contraindicated for use with nirmatrelvir/ritonavir • Did not receive other COVID-19 treatments • Assessed the intervention in five target populations: 1) not vaccinated, 2) received one or two doses of COVID-19 vaccine, 3) received a booster dose, 4) had a primary SARS-CoV-2 infection or 5) had been reinfected with SARS-CoV-2 | At least one required for cohort entry: • Age > 60 years • Body mass index > 25 kg/m^2^ • Current smoker • Chronic lung disease • Cardiovascular disease • Kidney disease • Immune dysfunction • Hypertension • Diabetes • Cancer | Nirmatrelvir/ritonavir | Not treated with other COVID-19 treatments | Within 5 days of SARS-CoV-2 test-positive date | 31524 | 224764 | NA | NA | 65.7 (13.4) | 61.0 (15.1) | 87.8 | 85.3 | 12.2 | 14.7 | Inverse probability of treatment weighting based on the estimated propensity score using a logistic regression model of all study patients. Clone-censor-weight approach with the cumulative inverse probability of censoring weight estimated using a logistic regression model. | Separate target trials were emulated by vaccination status. Results of two target trials were used in our analysis. Unvaccinated defined as no COVID-19 vaccination before the test-positive date and vaccinated defined as having received a COVID-19 vaccine booster (≥ 3 doses) before the test-positive date. | Included a history of SARS-CoV-2 infection as a covariate in the propensity score model. In a sensitivity analysis, excluded patients with a history of SARS-CoV-2 infection. |
| ***Lewnard 2023** included a limited number of individuals 12-19 years of age (nirmatrelvir/ritonavir=11 and control=11,054) from a total population of 133,426 so the majority (92%) were adults.  Abbreviations: BC, British Columbia; Comp, comparator; COVID-19, coronavirus disease 2019; CDC, US Centers for Disease Control and Prevention; EUA, emergency use authorisation; FDA, Food and Drug Administration; ICU, intensive care unit; Int., intervention; IQR, interquartile range; mRNA, messenger ribonucleic acid; NA, not applicable; NHS, National Health Service; NR, not reported; SARS-CoV-2, severe acute respiratory syndrome coronavirus 2; SD, standard deviation; VA; Veterans Affairs; VHA, Veterans Health Administration. | | | | | | | | | | | | | | | | | | | | | | | | | |

**Table S5.** Reviewers’ risk of bias judgments for each study domain and rationale for the overall risk of bias judgment

| **Study** | **D1 Confounding** | **D2 Selection of participants** | **D3 Classification of interventions** | **D4 Deviations from intended intervention** | **D5 Missing data** | **D6 Measurement of outcomes** | **D7 Selection of the reported result** | **Overall** | **Rationale** |
| --- | --- | --- | --- | --- | --- | --- | --- | --- | --- |
| Aggarwal 2023 | Serious | Moderate | Low | Low | Moderate | Low | Moderate | Serious | Judged to be at serious risk of bias for domain 1 as the study did not measure the confounding domain of concomitant prescription of interacting drugs. |
| Bajema 2023 | Moderate | Low | Low | Low | Low | Low | Moderate | Moderate | Judged to be at moderate risk of bias on two domains and low risk on the remaining domains. |
| Bhatia 2023 | Serious | Moderate | Moderate | No information | No information | Moderate | Moderate | Serious | Judged to be at serious risk of bias for domain 1 as the study did not measure or control for confounding domains such as vaccination, obesity, interacting drugs, or other COVID-19 treatments. |
| Butt 2023  (molnupirivir) | Serious | Serious | Low | No information | Moderate | Low | Moderate | Serious | Judged to be at serious risk of bias for domain 1 as the analysis used exact matching on a limited number of confounding domains. This does not adequately control for all key confounding domains. At serious risk on domain 2 since there is potentially significant immortal time bias (misclassification of follow-up time) for those assigned to molnupiravir within three days of diagnosis, as patients could be unexposed between days 0 to 2. |
| Butt 2023  (nirmatrelvir/ritonavir) | Serious | Serious | Low | No information | Serious | Low | Moderate | Serious | Judged to be at serious risk of bias for domain 1 as the analysis did not control for key confounding domains (i.e., timing of oral antiviral drug initiation and potentially interacting drugs). At serious risk on domain 2 since there is potentially significant immortal time bias (misclassification of follow-up time) for those assigned to nirmatrelvir/ritonavir within three days of diagnosis, as patients could be unexposed between days 0 to 2. At serious risk on domain 5 because there was a large amount of missing data for testing dates and body mass index (an important confounder) which was addressed inappropriately by excluding 11,203 patients. |
| Cegolon 2023 | Serious | Serious | Moderate | No information | Moderate | Moderate | Moderate | Serious | Judged to be at serious risk of bias for domain 1 as the analysis did not control for important confounding domain of potentially interacting drugs. At serious risk on domain 2 since the time to presentation at clinic partly defined inclusion in the control group and is strongly associated with receiving pharmacological intervention (only given if presented within 5 days) and outcome of hospitalization. |
| Dormuth 2023 | Moderate | Low | Low | No information | Low | Low | Moderate | Moderate | Judged to be at moderate risk of bias on two domains and low risk or no information on the remaining domains. |
| Dryden-Peterson 2023 | Moderate | Moderate | Low | Low | No information | Low | Moderate | Moderate | Judged to be at moderate risk of bias on three domains and low risk or no information on the remaining domains. |
| Evans 2023 | Serious | Low | Low | No information | Low | Low | Moderate | Serious | Judged to be at serious risk of bias for domain 1 as the study did not measure the confounding domains of concomitant prescription of interacting drugs or disease severity (time-varying confounder). |
| Faust 2023 | Serious | Serious | No information | No information | No information | Moderate | Moderate | Serious | Judged to be at serious risk of bias for domain 1 as vaccination status in terms of number of doses does not appear to be valid in the data source. It is also unclear which variables were included in the propensity score model (not described). At serious risk on domain 2 since the intervention and control groups were identified with separate queries in the data, thus all individuals receiving the intervention have potential for missing follow-up time between the diagnosis of COVID-19 and treatment initiation (immortal time). |
| Ganatra 2023 | Serious | Serious | No information | No information | No information | Moderate | Moderate | Serious | Judged to be at serious risk of bias on domain 1 as vaccination status in terms of number of doses does not appear to be valid in the data source. It is also unclear which variables were included in the propensity score model (not described). Did not account for a key confounding domain of concomitant prescriptions for interacting drugs. At serious risk on domain 2, because the intervention group and control group were identified with separate queries in the data, thus all individuals receiving the intervention have the potential for missing follow-up time between the diagnosis of COVID-19 (immortal time). |
| Gmizic 2023 | Critical | Serious | No information | No information | No information | Moderate | Moderate | Critical | Judged to be at critical risk of bias for domain 1 as the method of confounding domain selection is not appropriate (univariable statistical significance) and did not control for sex in the regression model, and potentially included post-intervention variables (pneumonia, laboratory tests) which makes the study too problematic to interpret. |
| Kwok 2023 | Critical | Moderate | Moderate | Low | Serious | Low | Moderate | Critical | Judged to be at critical risk of bias for domain 1 as important confounding domains were not adjusted for (e.g., timing of oral antiviral initiation, interacting drugs, comorbidities such as immunosuppressants) which makes the study too problematic to interpret. |
| Lewnard 2023 | Serious | Moderate | Low | Serious | Moderate | Low | Moderate | Serious | Judged to be at serious risk of bias on domain 1 as important confounding domains were not adjusted for (e.g., interacting drugs) nor potential time varying confounding (used time-varying exposure model) and domain 4 because of large imbalances in patients censored in intervention group before end of follow-up and switching treatments. |
| Liu 2023 | Serious | Critical | No information | No information | No information | Moderate | Serious | Critical | Judged to be at critical risk on domain 2 since a large amount of follow-up time is missing from both groups as patients with the outcome in first five days were excluded and this is expected to be the highest risk period (rate ratio is not constant). Outcomes were only included if they occurred between day 10 to 30 after the index date. At serious risk on domain 1 as the analysis did not control for important confounding domains such as vaccination, interacting drugs, or timing from COVID-19 diagnosis to treatment initiation. Serious risk on domain 7 because there is a discrepancy in supplementary material for patient cohort, described as asthma not patients with substance use disorder. |
| Low 2023 | Serious | Low | Low | No information | Moderate | Low | Moderate | Serious | Judged to be at serious risk of bias for domain 1 as the study did not control for at least one important domain (e.g., potentially interacting drugs). |
| Lui 2023 | Serious | Moderate | Low | No information | Moderate | Low | Moderate | Serious | Judged to be at serious risk of bias for domain 1 as the study did not control for at least one important domain such as antidiabetic medication, a marker of diabetes severity. It also seems some codes were recorded at any time based on supplementary material and the analysis did not control for time from COVID-19 diagnosis to treatment initiation. |
| Najjar-Debbiny 2023 (molnupirivir) | Moderate | Low | Low | No information | Low | Low | Moderate | Moderate | Judged to be at moderate risk of bias on two domains and low risk or no information on the remaining domains. |
| Najjar-Debbiny 2023 (nirmatrelvir/ritonavir) | Serious | Low | Low | No information | Low | Low | Moderate | Serious | Judged to be at serious risk of bias for domain 1 as the study modelled the intervention as time dependent exposure but no other time dependent confounders were adjusted in model (e.g., disease severity, vital signs (e.g. oxygen saturation) and the method of baseline confounding domain selection is not appropriate (backwards selection based on statistical significance) |
| Paraskevis 2023 | Critical | Serious | Low | No information | Critical | Low | Serious | Critical | Judged to be at critical risk of bias for domain 1 as the most confounding domains were not adjusted for in the regression models and adjusted for adherence (post-intervention variable). Judged to be at critical risk of bias for domain 5 as the baseline comorbidity data were not available for the untreated controls which makes the study too problematic to interpret. |
| Petrakis 2023 | Critical | Serious | Moderate | No information | No information | Low | Serious | Critical | Judged to be at critical risk of bias for domain 1 as the study did not control for important confounding domains (e.g., COVID-19 outcome severity risk factors, timing of oral antiviral initiation, interacting drugs). Judged to be at serious risk of bias for domain 2 and domain 7 which makes the study too problematic to interpret. |
| Schwartz 2023 | Moderate | Moderate | Low | No information | Moderate | Low | Moderate | Moderate | Judged to be at moderate risk of bias on three domains and low risk or no information on the remaining domains. |
| Shah 2023 | Serious | Moderate | Low | No information | No information | Low | Moderate | Serious | Judged to be at serious risk of bias for domain 1 as the study modelled the intervention as time dependent exposure but no other time dependent confounders were adjusted in model (e.g., disease severity, vital signs (e.g. oxygen saturation). Time-varying confounding likely present since those with more severe disease would either seek treatment and switch to treated group or be hospitalized early, and thus be ineligible for treatment as an outpatient. The validity and reliability of health conditions in the dataset were not clearly reported. |
| Tazare 2023 | Moderate | Low | Low | Low | Low | Low | Low | Moderate | Judged to be at moderate risk of bias on one domain and low risk on the remaining domains. |
| Van Heer 2023 | Serious | Serious | Moderate | No information | Moderate | Low | Moderate | Serious | Judged to be at serious risk of bias for domain 1 as the study did not control for at least one important domain such as comorbidities or known risk factors for being prescribed oral antiviral and outcome and used a proxy measure of recent hospitalisations. Serious risk on domain 2 as the person time between COVID-19 diagnosis and treatment initiation is immortal (potentially misclassified) and the rate ratio of outcomes is likely highest in the first 5 days after diagnosis. |
| Wee 2023 | Serious | Low | Low | No information | Moderate | Low | Moderate | Serious | Judged to be at serious risk of bias for domain 1 as At least one important confounding domain (e.g., interacting drugs) was not measured or controlled. |
| Wong 2022 | Serious | Moderate | Low | No information | Moderate | Low | Moderate | Serious | Judged to be at serious risk of bias for domain 1 as the study did not control for vaccination status, severe COVID-19 risk factors, or time to treatment initiation in the propensity score model. |
| Wu 2023 | Serious | Critical | Moderate | No information | No information | Moderate | Serious | Critical | Judged to be at critical risk on domain 2 since a large amount of follow-up time is missing from both groups as patients with the outcome in first five days were excluded and this is expected to be the highest risk period (rate ratio is not constant). Outcomes were only included if they occurred between day 10 to 30 after the index date. At serious risk on domain 1 as the analysis did not control for important confounding domains such as vaccination, interacting drugs, diabetes drugs, or timing from COVID-19 diagnosis to treatment initiation. Serious risk on domain 7 because there is no detailed code list and the report Seems to be a subgroup study of the larger database performed from the same authors of Liu 2023. |
| Xie 2023  (molnupirivir) | Moderate | Moderate | Low | No information | Low | Low | Moderate | Moderate | Judged to be at moderate risk of bias on three domains and low risk or no information on the remaining domains. |
| Xie 2023  (nirmatrelvir/ritonavir) | Moderate | Moderate | Low | No information | Low | Low | Moderate | Moderate | Judged to be at moderate risk of bias on three domains and low risk or no information on the remaining domains. |

**Figure S1.** Risk of bias of the included studies based on the Risk Of Bias In Non-randomized Studies – of Interventions (ROBINS-I) assessment framework for cohort studies. **A** presents a summary of the risk of bias for all the included studies while **B** presents the risk of bias judgement for each risk of bias domain and overall for each study.
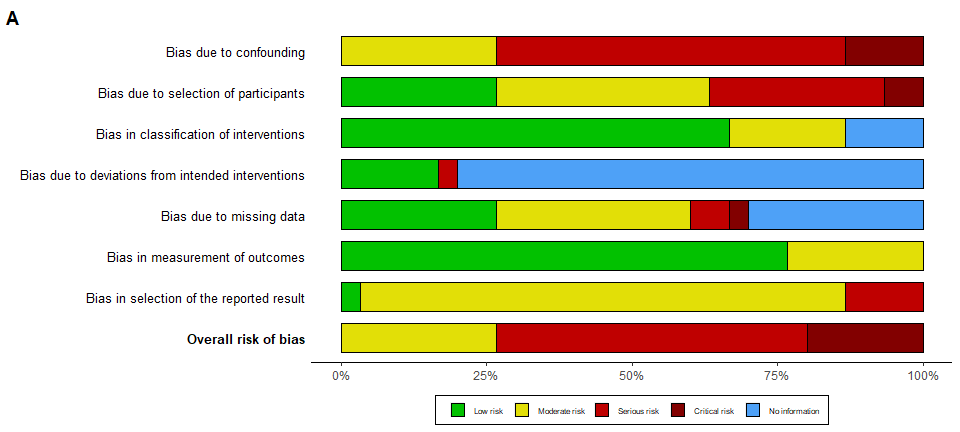


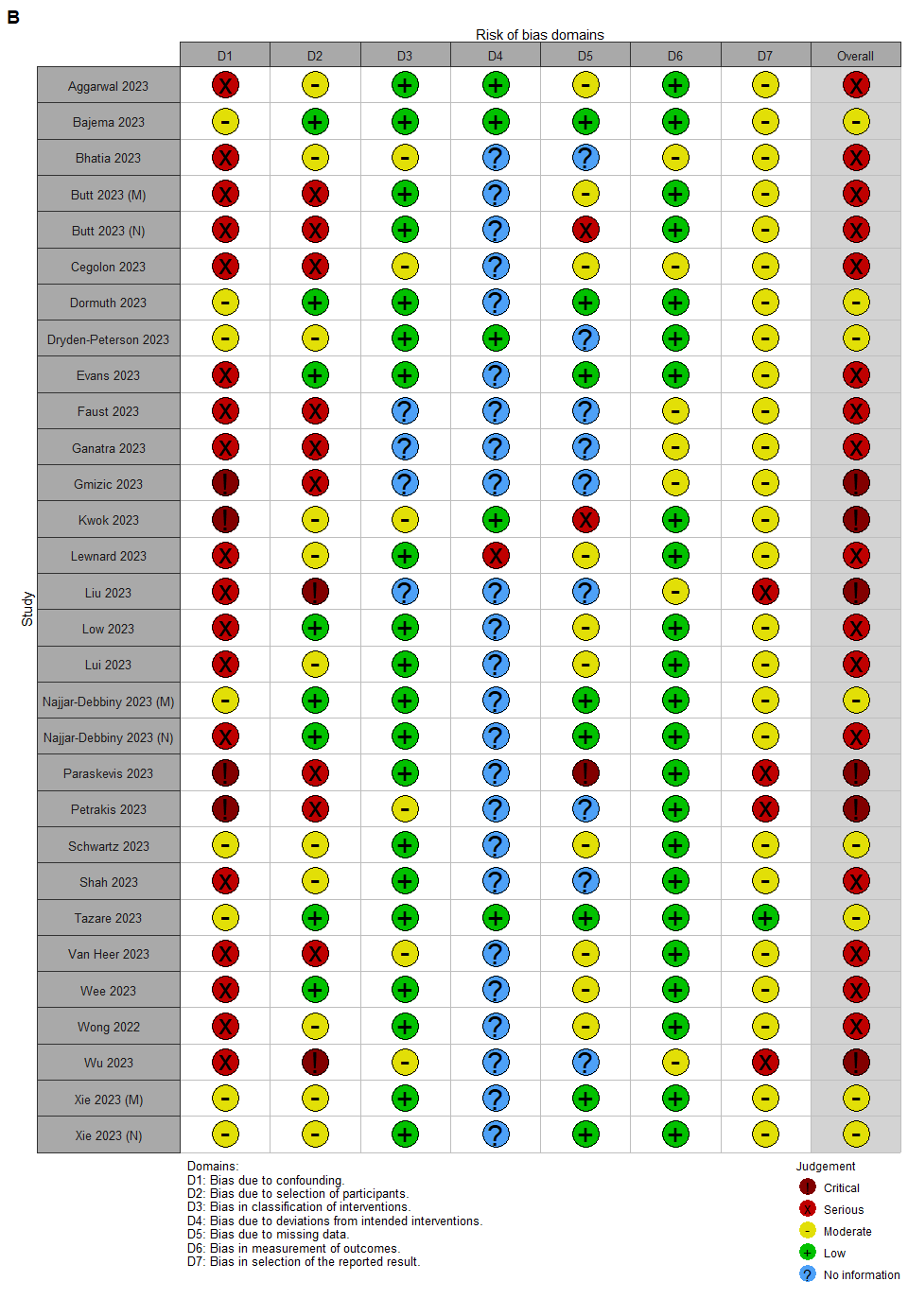


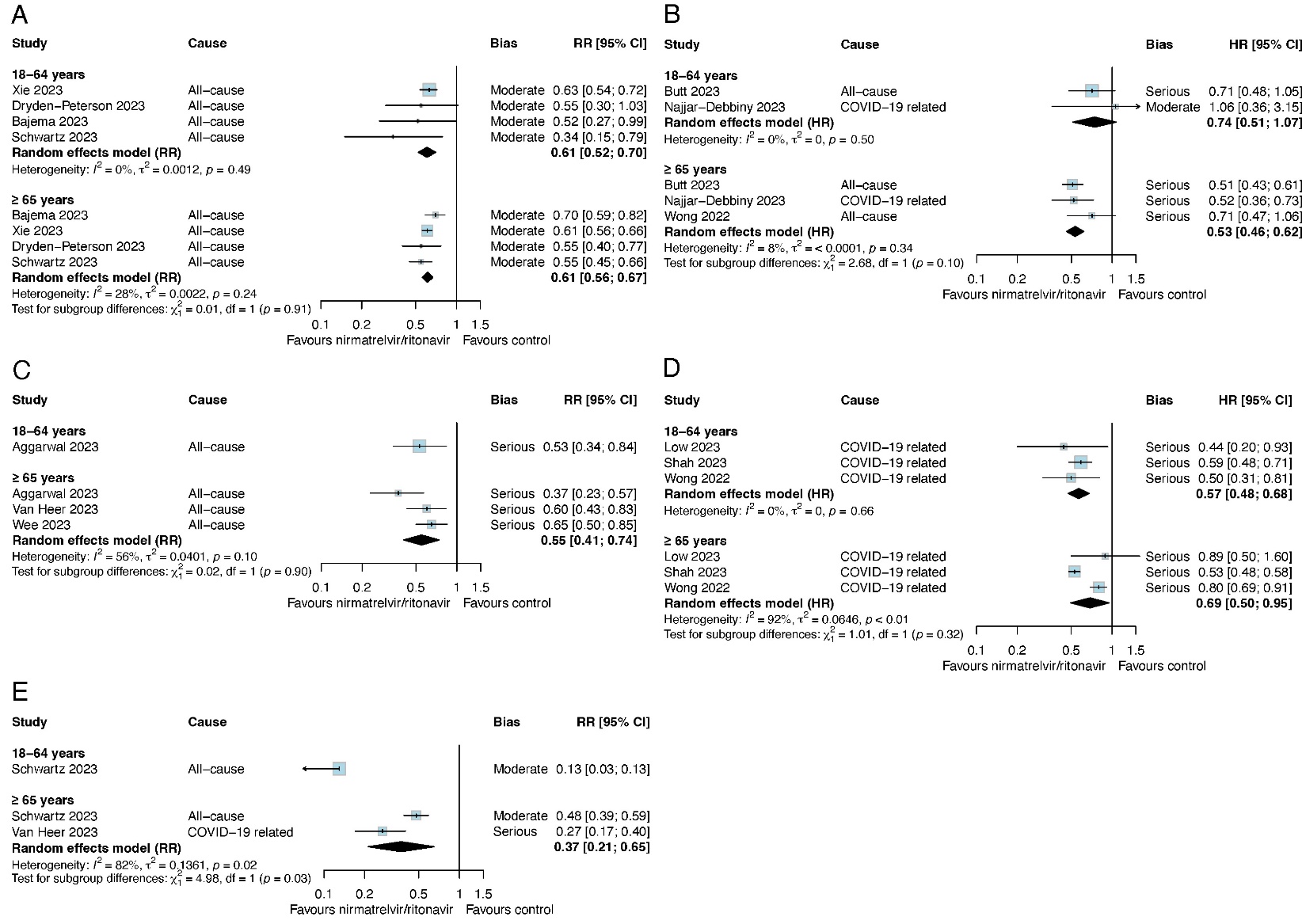

**Figure S2.** Forest plots showing the relative effectiveness of nirmatrelvir/ritonavir in adults stratified by age group (18-64 and ≥ 65 years) against the (A) composite outcome (risk ratio), (B) composite outcome (hazard ratio), (C) hospitalisation (risk ratio), (D) hospitalisation (hazard ratio) and (E) mortality (risk ratio). The size of the data markers (blue squares) reflects the weight of each study and its contribution to the pooled estimate.
**Abbreviations:** CI, confidence interval; HR, hazard ratio; RR, risk ratio.

**
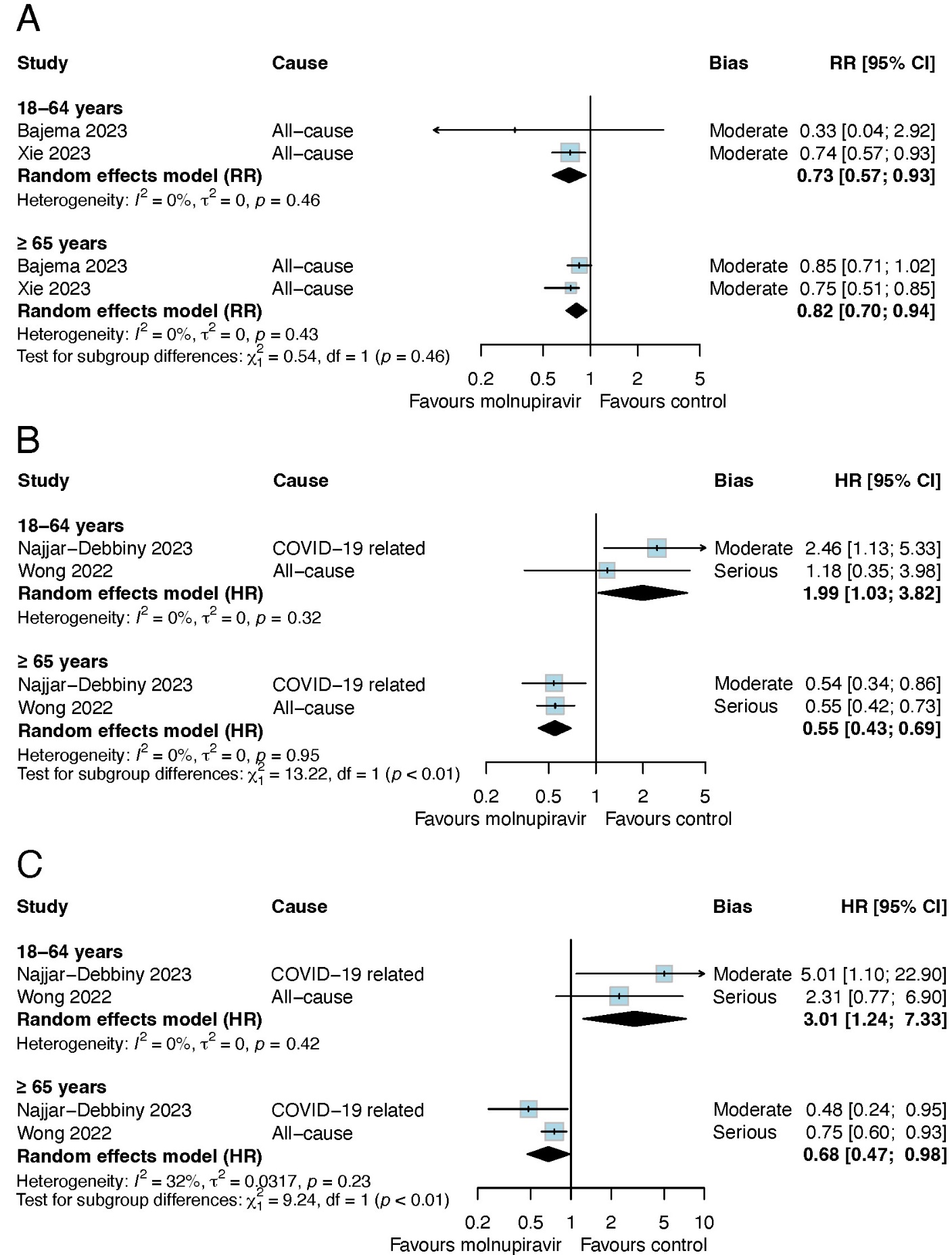
Figure S3.** Forest plots showing the relative effectiveness of molnupiravir in adults stratified by age group (18-64 and ≥ 65 years) against the (A) composite outcome (risk ratio), (B) composite outcome (hazard ratio), and (C) mortality (hazard ratio). There were an insufficient number of studies for hospitalisation to perform meta-analysis. The size of the data markers (blue squares) reflects the weight of each study and its contribution to the pooled estimate.
**Abbreviations:** CI, confidence interval; HR, hazard ratio; RR, risk ratio.

**Table S6.** Sensitivity analyses of the relative effectiveness of nirmatrelvir/ritonavir and molnupiravir in adult outpatients with COVID-19 pooled by relative effect measure as reported in the original studies (hazard ratio, odds ratio, or risk ratio). Analyses were performed in the overall study population that accounted for vaccination status (1), vaccinated patients (2) and unvaccinated patients (3). Pooled estimates are those of the effect measure of interest. Abbreviations: HR, hazard ratio; RR, risk ratio; OR, odds ratio.

| **Antiviral** | **Outcome** | **Effect measure** | **Number of studies** | **Pooled estimate [95% CI]** | **I^2^ (%)** |
| --- | --- | --- | --- | --- | --- |
| 1. Overall cohort accounting for vaccination status | | | | | |
| **Nirmatrelvir/ritonavir** | Composite outcome | | | | |
|  |  | HR | 5 | 0.54 [0.47; 0.61] | 0.0 |
|  |  | OR | 2 | 0.63 [0.43; 0.92] | 47.0 |
|  |  | RR | 4 | 0.62 [0.58; 0.66] | 0.0 |
|  | Hospitalisation |  |  |  |  |
|  |  | HR | 3 | 0.62 [0.46; 0.84] | 94.6 |
|  |  | OR | 5 | 0.47 [0.34; 0.65] | 75.9 |
|  |  | RR | 2 | 0.69 [0.58; 0.82] | 20.1 |
|  | Mortality |  |  |  |  |
|  |  | HR | 1 | 0.34 [0.22; 0.52] | -- |
|  |  | OR | 3 | 0.33 [0.19; 0.59] | 75.9 |
|  |  | RR | 2 | 0.25 [0.15; 0.40] | 0.0 |
| **Molnupiravir** | Composite outcome | | | | |
|  |  | HR | 4 | 0.74 [0.51; 1.06] | 85.7 |
|  |  | OR | 0 | -- | -- |
|  |  | RR | 2 | 0.75 [0.67; 0.85] | 31.5 |
|  | Hospitalisation |  |  |  |  |
|  |  | HR | 1 | 0.98 [0.90; 1.06] | -- |
|  |  | OR | 1 | 0.71 [0.58; 0.87] | -- |
|  |  | RR | 2 | 0.87 [0.72; 1.06] | 68.7 |
|  | Mortality |  |  |  |  |
|  |  | HR | 2 | 0.77 [0.62; 0.94] | 0.0 |
|  |  | OR | 1 | 0.45 [0.38; 0.54] | -- |
|  |  | RR | 2 | 0.30 [0.21; 0.45] | 28.4 |
| 2. Vaccinated | | | | | |
| **Nirmatrelvir/ritonavir** | Composite outcome | | | | |
|  |  | HR | 3 | 0.55 [0.46; 0.66] | 0.0 |
|  |  | OR | 2 | 0.58 [0.48; 0.69] | 24.7 |
|  |  | RR | 3 | 0.65 [0.59; 0.70] | 0.0 |
|  | Hospitalisation |  |  |  |  |
|  |  | HR | 3 | 0.59 [0.45; 0.75] | 60.4 |
|  |  | OR | 2 | 0.46 [0.31; 0.68] | 0.0 |
|  |  | RR | 0 | -- | -- |
|  | Mortality |  |  |  |  |
|  |  | HR | 0 | -- | -- |
|  |  | OR | 1 | 0.54 [0.43; 0.67] | -- |
|  |  | RR | 0 | -- | -- |
| **Molnupiravir** | Composite outcome | | | | |
|  |  | HR | 2 | 1.16 [0.61; 2.22] | 17.4 |
|  |  | OR | 0 | -- | -- |
|  |  | RR | 2 | 0.79 [0.63; 1.00] | 63.0 |
|  | Hospitalisation |  |  |  |  |
|  |  | HR | 1 | 0.66 [0.42; 1.03] | -- |
|  |  | OR | 0 | -- | -- |
|  |  | RR | 0 | -- | -- |
|  | Mortality |  |  |  |  |
|  |  | HR | 2 | 1.30 [0.52; 3.21] | 28.7 |
|  |  | OR | 0 | -- | -- |
|  |  | RR | 0 | -- | -- |
| 3. Unvaccinated | | | | | |
| **Nirmatrelvir/ritonavir** | Composite outcome | | | | |
|  |  | HR | 4 | 0.59 [0.52; 0.69] | 0.0 |
|  |  | OR | 1 | 0.44 [0.23; 0.84] | -- |
|  |  | RR | 2 | 0.45 [0.09; 2.10] | 90.3 |
|  | Hospitalisation |  |  |  |  |
|  |  | HR | 2 | 0.62 [0.41; 0.93] | 93.5 |
| **Nirmatrelvir/ritonavir** |  | OR | 1 | 0.46 [0.27; 0.78] | -- |
|  |  | RR | 0 | -- | -- |
|  | Mortality |  |  |  |  |
|  |  | HR | 1 | 0.44 [0.30; 0.65] | -- |
|  |  | OR | 1 | 0.34 [0.16; 0.73] | -- |
|  |  | RR | 0 | -- | -- |
| **Molnupiravir** | Composite outcome | | | | |
|  |  | HR | 2 | 0.60 [0.43; 0.85] | 30.3 |
|  |  | OR | 0 | -- | -- |
|  |  | RR | 2 | 0.75 [0.55; 1.02] | 46.6 |
|  | Hospitalisation |  |  |  |  |
|  |  | HR | 1 | 1.01 [0.93; 1.10] | -- |
|  |  | OR | 0 | -- | -- |
|  |  | RR | 0 | -- | -- |
|  | Mortality |  |  |  |  |
|  |  | HR | 2 | 0.59 [0.24; 1.46] | 73.8 |
|  |  | OR | 0 | -- | -- |
|  |  | RR | 0 | -- | -- |
